# Identification of PPREs and PPRE associated genes in the human genome: insights into related kinases and disease implications

**DOI:** 10.3389/fimmu.2024.1457648

**Published:** 2024-10-02

**Authors:** Pritha Saha, Priti Talwar

**Affiliations:** Apoptosis and Cell Survival Research Laboratory, School of Biosciences and Technology, Vellore Institute of Technology, Vellore, India

**Keywords:** peroxisome proliferator activated receptor response element, kinase, network analysis, protein protein interaction, UCSC genome browser, genome workbench

## Abstract

**Introduction:**

“Peroxisome Proliferator-Activated Receptors” (PPARs) belong to the class of transcription factors (TF) identified as Nuclear Receptors (NR). Upon activation by peroxisome proliferators (PPs), PPARs modulate a diverse range of genes, consequently regulating intra-cellular lipid metabolism, glucose uptake, apoptosis, and cell proliferation. Subsequent to the heterodimerization of Retinoid X Receptors (RXR) with PPARs induced by the binding of activators to PPARs, facilitates the binding of the resulting complex to Peroxisome Proliferator-Activated Receptors Response Elements (PPRE), with a consensus sequence 5’AGGTCANAGGTCA-3’, and regulate the transcription of the targeted genes.

**Methods:**

A comprehensive screening of PPRE within the whole human genome was performed using the Genome Workbench and UCSC Genome Browser to find the associated genes. Subsequently, the kinase subset was isolated from the extracted list of PPRE-related genes. Functional enrichment of the kinases was performed using FunRich, ToppGene, and ShinyGO. Network analysis and enrichment studies were then further performed using NDEx to elucidate these identified kinases' connections and significance. Additionally, the disease association of the PPRE kinases was analyzed using DisGeNET data in R studio and the COSMIC dataset.

**Results:**

A comprehensive analysis of 1002 PPRE sequences within the human genome (T2T), yielded the identification of 660 associated genes, including 29 kinases. The engagement of these kinases in various biological pathways, such as apoptosis, platelet activation, and cytokine pathways, revealed from the functional enrichment analysis, illuminates the multifaceted role of PPAR in the regulation of cellular homeostasis and biological processes. Network analysis reveals the kinases interact with approximately 5.56% of the Human Integrated Protein-Protein Interaction rEference (HIPPIE) network. Disease association analysis using DisGeNET and COSMIC datasets revealed the significant roles of these kinases in cellular processes and disease modulation.

**Discussion:**

This study elucidates the regulatory role of PPAR-associated genes and their association with numerous biological pathways. The involvement of the kinases with disease-related pathways highlights new potential for the development of therapeutic strategies designed for disease management and intervention.

## 1 Introduction

**Figure 1:**
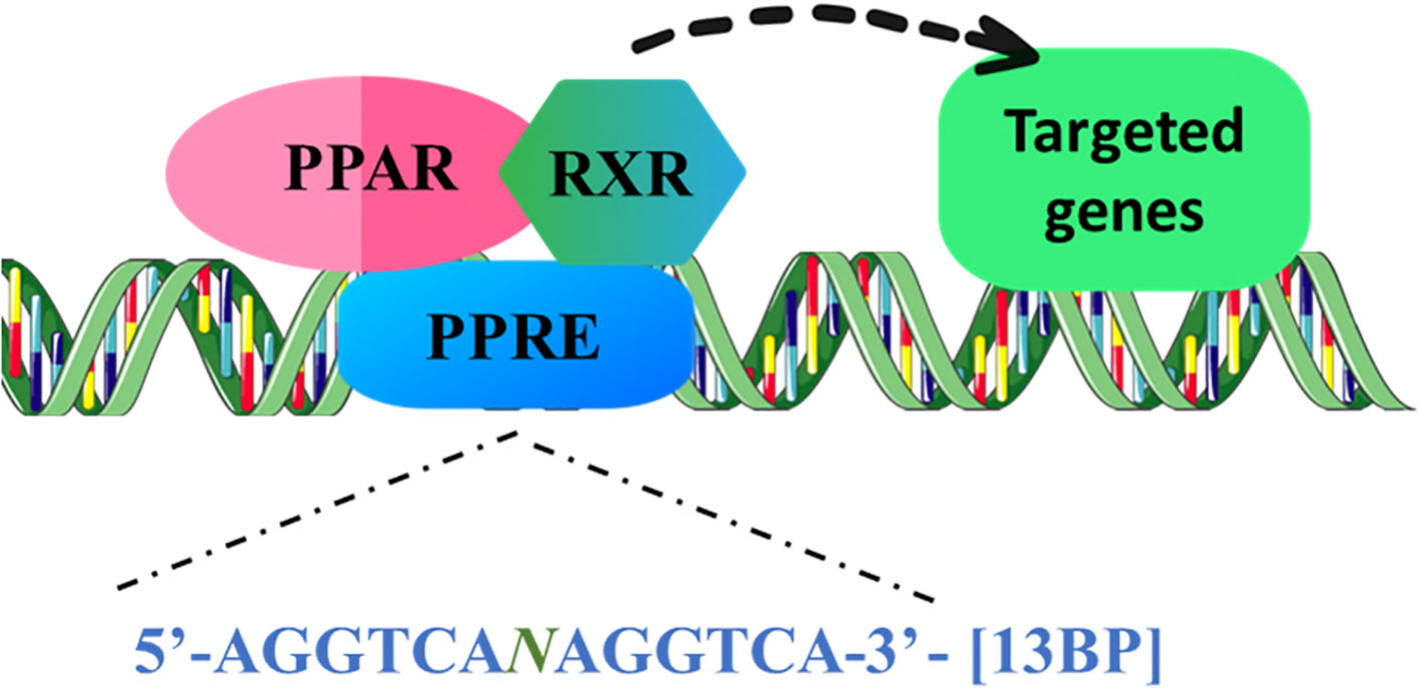
PPARs are ligand-induced transcription factors of nuclear hormone receptor superfamily. PPAR binds to the 13bp conserved sequence, PPRE as a conjugate with the RXR, and can recruit coactivator or corepressor proteins to mediate the gene expression of lipid metabolism, glucose homeostasis, inflammation, and apoptosis regulators. (PPARs, “Peroxisome proliferator-activated receptors”; PPRE, PPAR response element; RXR, “Retinoid X Receptor”).

“Peroxisome Proliferator-Activated Receptors” (PPARs) allied to the class of transcription factors (TF) identified as Nuclear Receptors (NR), which can be categorized into three separate subfamilies: α, β/δ, and γ. PPARs exhibit differential responses to distinct ligands, resulting in the modulation of gene expression and regulation of multiple cellular events. Upon stimulation by hypolipidemic substances such as peroxisome proliferators (PPs), it has been shown that PPARs play a crucial role in regulating a wide array of genes. This regulation includes modulation of intracellular lipid metabolism, glucose uptake, apoptosis, and cell proliferation (1). PPAR structure includes four domains, starting from the N-terminal A/B domain, C and D domain, and E/F domain. Ligand-independent activation of PPAR via Activation Function 1 (AF-1) is regulated by a poorly conserved region, the A/B domain. C domain comprises a DNA-binding domain (DBD) that adheres itself to the target genes’ promoter region’s “PPAR response element” (PPRE). A flexible link across the DBD to the E/F domain is facilitated by the hinge region in the D domain. The C-terminal i.e., the E/F domain facilitates the binding of a ligand (2). Subsequent heterodimerization of Retinoid X Receptors (RXR) with PPARs upon binding of the activators to the PPARs, promotes tethering of the complex to PPRE, with a consensus sequence 5’AGGTCANAGGTCA-3’ (**Figure 1**), and regulate the transcription of the targeted genes (3–5). Upon ligand binding, PPARs experience a structural transformation that results in the release of histone deacetylase (HDAC) co-repressors, thereby allowing PPARs to form a heterodimer with RXR. Subsequently, RNA polymerase II along with co-activators that possess histone acetyltransferase (HAT) activity are attracted to this complex, which interacts with response elements in target genes, facilitating chromatin remodeling and ultimately enhancing transcription (6).

PPARs play a critical role in modulating numerous essential biological processes, encompassing inflammation, cellular survival, and differentiation. Fatty acids, fibrates, and leukotriene B4, potential activators of PPAR- α conversely triggers transcription of genes involved in ω - and β-oxidation of fatty acids. PPARα exhibits significant expression levels in tissues characterized by high metabolic activity, including the liver, heart, skeletal muscle, intestinal mucosa, and brown adipose tissue. This receptor plays a crucial role in the regulation of lipid homeostasis through fatty acid metabolism, and its activation is associated with a reduction in lipid concentrations (7). PPARα boosts the capacity of cells, particularly in the liver, to mobilize and break down fatty acids, which is vital during periods of starvation when fatty acid oxidation serves as a source of energy. When fatty acid concentrations rise, PPARα gets activated, facilitating the absorption and oxidation of these fatty acids. This mechanism predominantly takes place in the liver and aids in preventing steatosis during fasting. The influx of fatty acids also enhances the transcription of genes regulated by PPARα, triggering various oxidation pathways, which include microsomal omega-oxidation, alongside mitochondrial and peroxisomal beta-oxidation (6, 7).

An essential function of PPARβ/δ is to control lipid and glucose balance. PPARβ/δ is expressed extensively across nearly all tissue types; nonetheless, it is especially prevalent in the liver, intestine, kidney, colon, abdominal adipose tissue, cutaneous epithelial tissues, and skeletal muscle, all of which play critical roles in lipid metabolism. It is involved in the catabolism of fatty acids, predominantly within skeletal and cardiac muscle tissues, and it modulates the concentrations of cholesterol in the bloodstream as well as the levels of glucose. Additionally, through the trans-repression of NF-κB-dependent signaling pathways, PPARβ/δ inhibits hepatic inflammation triggered by genetic, dietary, and chemical stimuli. This results in a decrease in the expression of pro-inflammatory cytokines like tumor necrosis factor (TNF), interleukin (IL)-1β, and IL-6 (6, 7). Both PPARα and PPARβ/δ are able to bind to the p65 subunit of the nuclear factor-κB (NF-κB) complex, which prevents the NF-κB from controlling genes linked to pro-inflammatory reactions (6).

PPARγ is predominantly found in adipocytes, where it is essential for lipid synthesis, maintaining energy balance, and the process of adipogenesis. It can also be located in the spleen, and large intestine, as well as in both white and brown adipose tissues. This receptor is vital for ensuring insulin sensitivity, managing lipoprotein metabolism, and controlling fat storage, and it plays a significant role in the formation of the placenta and the heart. PPARγ agonists specifically target white adipose tissue to promote adipogenesis and lipid accumulation, which consequently reduces serum lipid concentrations. They also enhance the production of adipokines such as resistin and adiponectin, thereby improving insulin sensitivity. Furthermore, PPARγ may facilitate the anti-inflammatory properties of polyunsaturated fatty acids (PUFAs), as n3 PUFA has been demonstrated to encourage hepatic regulatory T (Treg) cells by increasing PPARγ and transforming growth factor beta (TGF-β), thereby influencing liver inflammation (6–8).

Antiproliferative effects, antagonism of angiotensin II actions, and antioxidant mechanisms that inhibit the generation of reactive oxygen species (ROS) and activation of inflammatory mediators in the vasculature and cardiac tissue have all been observed with the use of PPAR-γ agonists, such as thiazolidinediones. These medications have shown the ability to restore endothelial dysfunction and decrease blood pressure in various models of hypertension. Their impact on the cardiovascular system includes anti-inflammatory and anti-fibrotic properties (1, 9).

The involvement of PPAR has been linked with multiple diseases like cancer, neurodegenerative diseases, diabetes, and pulmonary fibrosis. An elevated level of expression of PPAR-α is observed in cells like hepatocytes, cardiomyocytes, enterocytes, and the proximal tubule cells of the kidney due to their active fatty acid oxidation efficiency (10). The expression of PPAR-α in peripheral tissues plays a vital role in essential metabolic pathways associated with the pathophysiology of common diseases such as diabetes, hypertension, atherosclerosis, inflammation, cancer, and neurodegeneration. PPARα agonists are highly significant in managing dyslipidemia and metabolic syndromes by reducing plasma triglyceride levels (8, 8).

Although the precise role of PPAR-β/δ remains elusive, it is widely distributed throughout the body and could potentially influence lipid and fatty acid metabolism, particularly within the cardiac tissue (2). PPAR-γ is primarily involved in the process of adipogenesis, insulin sensitivity, and maintenance of pulmonary homeostasis. Its widespread presence is evident in various cell types such as adipocytes, pulmonary epithelial cells, fibroblasts, myocardium, smooth muscle cells, and inflammatory macrophages. Moreover, it demonstrates anti-fibrotic characteristics. Studies have shown that reducing PPARγ levels in isolated human or murine lung fibroblasts can intensify pro-fibrotic traits (9). Modifications in this ligand-responsive transcriptional regulator have been linked to various metabolic pathologies, including atherosclerosis, obesity, metabolic syndrome, dyslipidemias, type 2 diabetes mellitus, and neoplasia (8).

The cellular response to PPAR exhibits a state in which signal transduction activities mediated by kinases are of utmost importance in cellular adaptability. Hence, there is a need to comprehensively elucidate the characteristics of every kinase participating in the signaling processes mediated by the PPARs, as well as their potential implications in both physiological well-being and pathological conditions.

The recognition that reversible phosphorylation mechanisms regulate several physiological functions and that some abnormalities in these processes may occur in pathological states aligns with the budding emergence of kinase modulators against degenerative illnesses. Translational suppression enables the temporary alteration of the TF network, which is contingent upon the activity of regulatory factors and precursor proteins (11).

**Figure 2:**
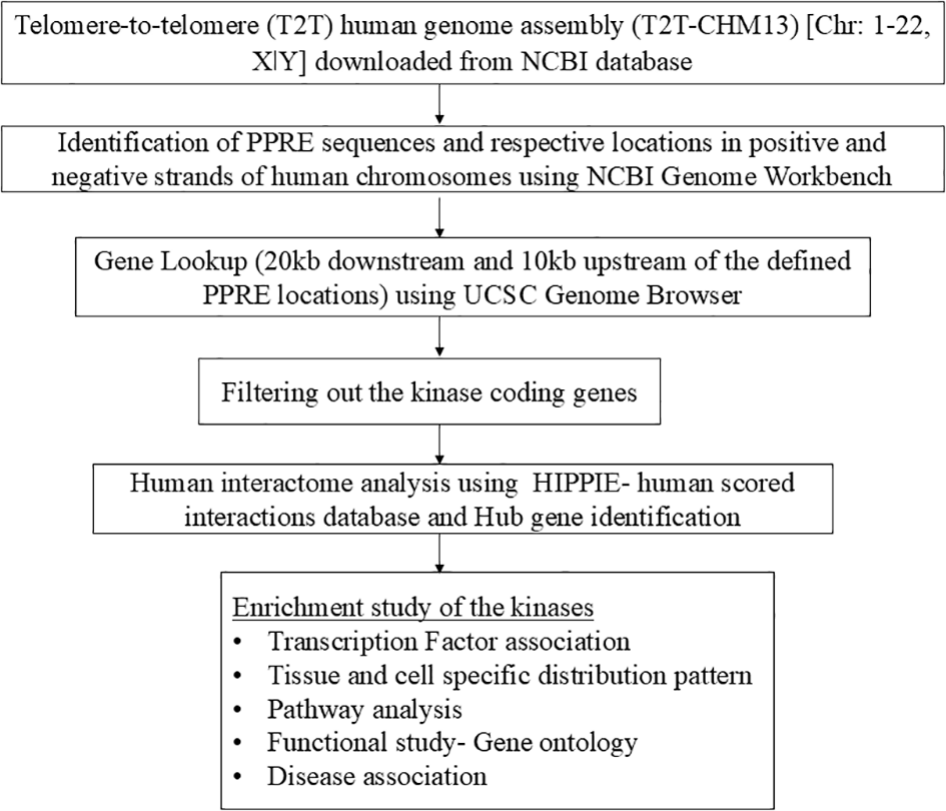
The overall workflow of the study is represented as a flowchart.

This study was designed to comprehensively screen the PPRE sequence present in the whole human genome and identification of PPAR-regulated genes which holds potential for further therapeutic interventions for disease progression, implementing a data-driven approach, and leveraging openly accessible lookup tools. The overall workflow of this study is given as a flowchart in **Figure 2**.

## 2 Materials and methods

### 2.1 Data acquisition

The recent rendition of the telomere-to-telomere (T2T) human genome assembly (T2T-CHM13) released in January, 2022 was acquired as individual chromosomes in FASTA format from the National Center for Biotechnology Information (NCBI) database.

### 2.2 Detection and visualization of PPRE sequence location in the human genome

The “Genome Workbench” (GW) serves as a tool designed for the comprehensive analysis of genomic data spanning various species (12). In this study, we employed GW 3.7.1 to identify the localization of the PPRE sequence across the human genome. The reference sequence dataset of each chromosome was uploaded onto the platform. Subsequently, a search for the PPRE query sequence was conducted on each chromosome to ascertain exact matches, facilitating the visualization and determination of the precise genomic coordinates of the PPRE sequence on both the positive and negative strands.

### 2.3 Identification of the PPRE related genes

The UCSC Genome Browser, a web-based platform overseen by the University of California, Santa Cruz (UCSC), enables interactive exploration and retrieval of genomic data across a diverse range of species. It provides a comprehensive set of annotations, facilitating in-depth analysis and supporting the download of genomic information (13). Within the scope of this investigation, the table browser tool was employed to elucidate the genes, with the PPRE location defined as the lookup regions. We have also looked for the genes 20kb downstream and 10kb upstream of the defined PPRE locations.

### 2.4 Filtration of the kinase coding genes

The process of phosphorylation mediated by protein kinases stands as a fundamental and ubiquitous cellular signaling mechanism in eukaryotic cells (14). The human kinases were retrieved from the UniProt downloadable repositories (15), released on June 2023. The PPRE-associated genes were further compared and matched with the human kinases to filter out the PPRE-associated kinases.

### 2.5 Human interactome analysis using NDEx and Cytoscape

The elucidation of protein interaction networks within human cells necessitates the systematic identification of all constituent elements. Robust and current compendiums of the human protein interactome have emerged from proteome-wide experimental investigations and extensive bioinformatics endeavors. Notably, the NDEx Project serves as an open-source platform facilitating the preservation, exchange, modification, and publication of biological network data (16) In the present study, the NDEx server was utilized to retrieve a comprehensive human protein-protein interactome (PPI), HIPPIE- Human Scored Interactions (version 2.3, released on 29^th^ April 2022) (17). Key genes were run against the human PPI and further, the high confidence interactions (confidence score ≥ 0.7) were visualized using Cytoscape 3.10.1. The cytoHubba plugin within Cytoscape was employed to identify the hub genes within the network based on closeness, degree, and betweenness (18).

### 2.6 Enrichment study of the hits

Modern data analysis algorithms are imperative for the analysis of genes/proteins derived from data-intensive studies. FunRich is a user-friendly, open-access bioinformatics tool that can be used for functional enrichment analysis of proteomics, genomics, lipidomics, and metabolomics datasets and it provides a graphical user interface for analysis (19). Functional enrichment of the hits was performed using the FunRich database (version: 3.1.3). Further transcriptional regulation, involvement in the biological pathways, and distributional analysis were also performed using FunRich. Subsequently, an analysis of Gene Ontology (GO) was performed to delineate the relevant pathways and terms associated with Biological Processes (BP) and Molecular Functions (MF), aiming to gain a deeper understanding of the underlying biological mechanisms at play. This analysis was conducted utilizing ShinyGO version 0.77 (20). Additional validation was done using ToppGene Suite, g:Profiler and DAVID (21–23). DisGeNET encompasses a compilation of genes and variants linked to human diseases (24). Disease association of the hits was achieved by retrieving the DisGeNET dataset for the selected hits. The dataset was filtered out by eliminating phenotypes from disease type and a cut-off score of Gene Disease Association (GDA) > 0.3 was given. A heat map of the gene and corresponding diseases was created using R studio. The COSMIC dataset, also known as the Catalogue of Somatic Mutations in Cancer, serves as a comprehensive repository for investigating the effects of somatic mutations in the context of human cancer (25). The COSMIC dataset was also analyzed using FunRich.

## 3 Results

### 3.1 Whole genome detection of PPRE

Investigation of the genomic distribution of PPRE sequences revealed their ubiquity across autosomes and sex chromosomes. In the latest telomere-to-telomere (T2T) human genome assembly, T2T-CHM13, a total of 1002 PPRE sequences were identified and their respective genomic locations were determined using Genome Workbench. Among these, 490 sequences were positioned on the positive strand, while 512 sequences were located on the negative strand (**Supplementary Tables 1, 2**).

### 3.2 Extraction of PPRE associated genes

The locations of PPRE sequences were used further to identify genes associated with PPRE. We have also looked for the genes 20kb downstream and 10kb upstream of the defined PPRE locations. A total of 311 and 349 genes were discerned on the positive and negative strands, respectively, of the human chromosome (**Supplementary Tables 1, 2**).

### 3.3 Filtration of the kinase coding genes and their interaction with human PPI

**Figure 3:**
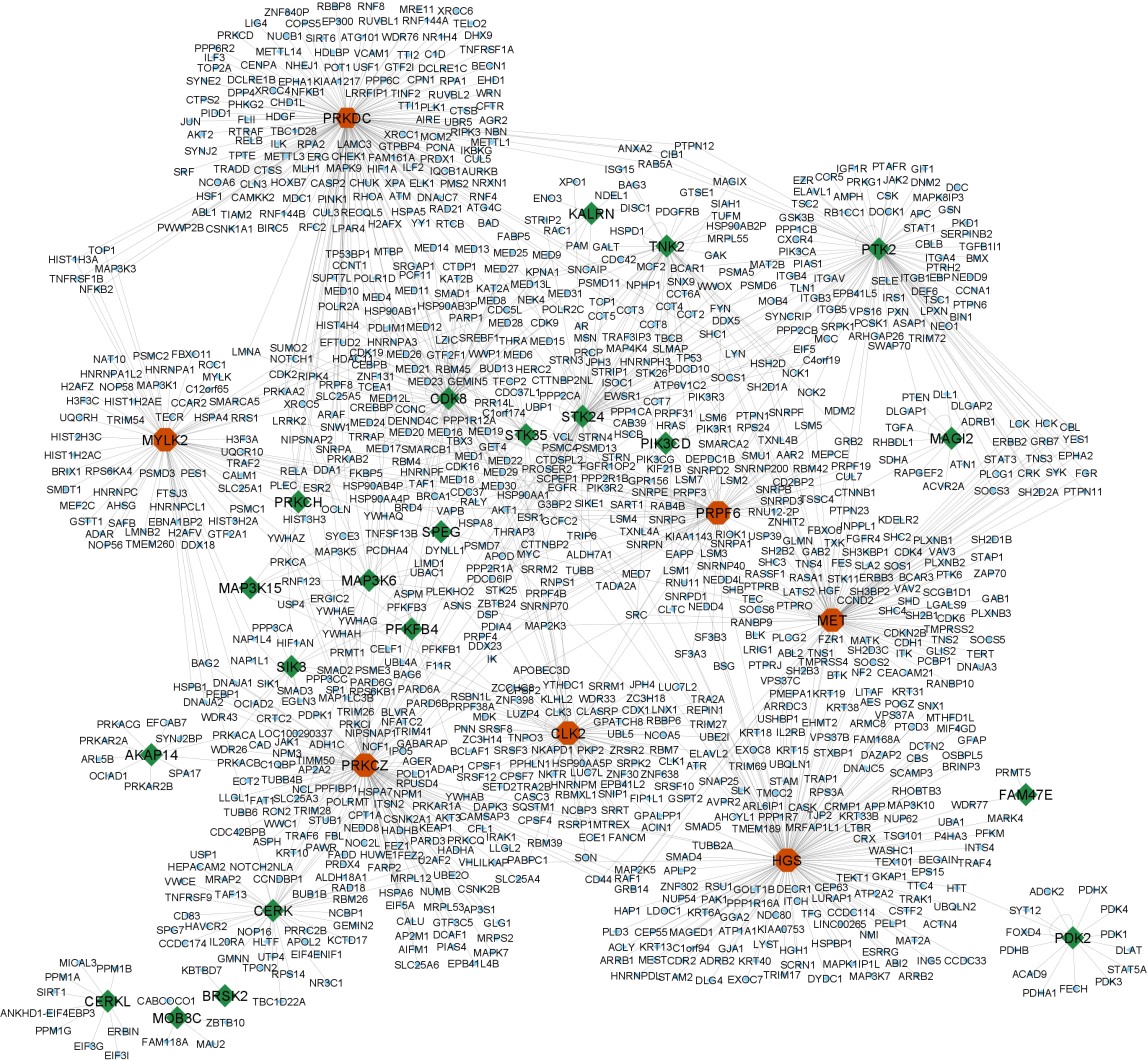
High confidence interactions (confidence score ≥ 0.7) of the PPRE-associated kinases with human PPI (HIPPIE- Human Scored Interactions database) revealed their ability to regulate 5.56% of human PPI. The query kinases are highlighted: green- PPRE-associated kinases; red- hub genes (kinases) within the network.

A subset of 29 kinases was extracted from the list of PPRE-associated genes by cross-referencing them with human kinases, retrieved from the UniProt dataset, released on June 2023 (**Table 1**). These 29 key proteins were subsequently utilized as query entities within the NDEx platform against the human PPI, HIPPIE- Human Scored Interactions, and the high confidence interactions (confidence score ≥ 0.7), all of which are experimentally validated were visualized using Cytoscape 3.10.1 (**Figure 3**) (**Supplementary Table 3**). Notably, the analysis revealed that among the 19485 nodes of human PPI, our query interacts with 1084 nodes (confidence score ≥ 0.7), which infers that around 5.56% of human PPI could be subject to regulation by PPRE-associated kinases. The top 10 hub genes for closeness, degree, and betweenness were identified. A total of 13 hub genes were identified within the network based on closeness, degree, and betweenness. Out of which 7 genes (PRKDC, PRKCZ, HGS, MET, CLK2, PRPF6, MYLK2) were present in all three, closeness, degree, and betweenness (**Figure 3**) (**Supplementary Table 4**, **Supplementary Figure 3**).

**Table 1:** List of kinases associated with PPRE and their number of interacting nodes (confidence score ≥ 0.7) with human PPI, in the HIPPIE- Human Scored Interactions database.

Sl. No	CHR	Gene	Gene Name	Number of interacting nodes (Confidence score ≥ 0.7)
1	chr8	PRKDC	Protein kinase, DNA-activated, catalytic subunit	167
2	chr17	HGS	Hepatocyte growth factor-regulated tyrosine kinase substrate	155
3	chr1	PRKCZ	Protein kinase C zeta	128
4	chr7	MET	RNA guanine-7 methyltransferase	113
5	chr8	PTK2	Protein tyrosine kinase 2	94
6	chr1	CLK2	Telomere maintenance 2	88
7	chr13	CDK8	Cyclin dependent kinase 8	81
8	chr20	PRPF6	Pre-mRNA processing factor 6	77
9	chr20	MYLK2	Myosin light chain kinase 2	77
10	chr13	STK24	Serine/threonine kinase 24	70
11	chr3	TNK2	Tyrosine kinase nonreceptor 2	42
12	chr22	CERK	Ceramide kinase	41
13	chr1	MAP3K6	Mitogen-activated protein kinase kinase kinase 6	21
14	chr20	STK35	Serine/threonine kinase 35	15
15	chr11	SIK3	SIK family kinase 3	14
16	chr17	PDK2	Pyruvate dehydrogenase kinase 2	14
17	chr7	MAGI2	Membrane associated guanylate kinase, WW and PDZ domain containing 2	12
18	chrX	AKAP14	A-kinase anchoring protein 14	10
19	chr2	CERKL	Ceramide kinase like	9
20	chr3	KALRN	Kalirin rhogef kinase	7
21	chr3	PFKFB4	6-phosphofructo-2-kinase/fructose-2,6-biphosphatase 4	7
22	chr1	PIK3CD	Phosphatidylinositol-4,5-bisphosphate 3-kinase catalytic subunit delta	6
23	chr14	PRKCH	Protein kinase C eta	6
24	chrX	MAP3K15	Mitogen-activated protein kinase kinase kinase 15	5
25	chr1	MOB3C	MOB kinase activator 3C	4
26	chr2	SPEG	Striated muscle enriched protein kinase	4
27	chr4	FAM47E	Family with sequence similarity 47 member E	2
28	chr11	BRSK2	BR serine/threonine kinase 2	1
29	chr19	SRRM5	Serine/arginine repetitive matrix 5	0

### 3.4 Cluster analysis of the distribution of the key proteins

**Figure 4:**
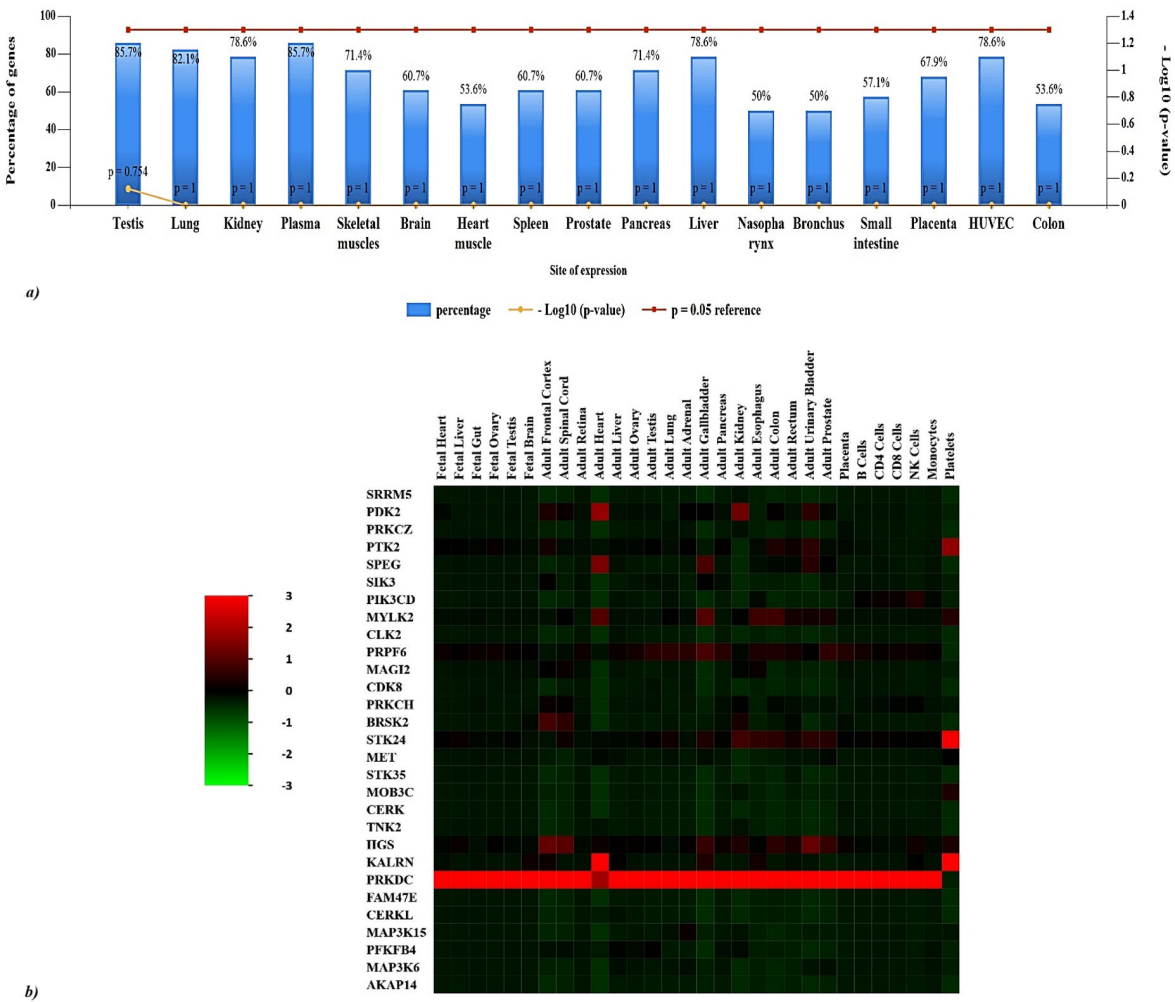
Distributional analysis of key proteins across human tissues. **(A)** 85.7% of genes are expressed in plasma and testis and 82.1% of the genes are predominantly expressed in the lung. The genes are also significantly expressed in liver, human umbilical vein endothelial cells (HUVEC), liver, and kidney. **(B)** Heatmap of gene expression levels across various fetal and adult tissues, as well as specific cell types (Green represents lower expression, red indicates higher expression): PRKDC is highly expressed in all tissues except platelets, and KALRN is significantly expressed in adult heart and platelets. HGS and PRPF6 show relatively higher expression in adult gallbladder and prostate. In the lung, a significant expression of PRPF6 is observed. In contrast, genes like FAM47E, CERKL, SIK3, PFKFB4, MAP3K6, AKAP14 and MOB3C exhibit low expression in most tissues. Noteworthy patterns include MYLK2 being highly expressed in the adult heart, adult gallbladder, esophagus and colon, while PDK2 and STK24 are strongly expressed in the adult kidney, and STK24 additionally in the esophagus, colon, urinary bladder, prostate, and platelets. HGS is also strongly expressed in the adult frontal cortex, spinal cord, gallbladder, kidney, colon, urinary bladder, prostate and PRPF6 is highly expressed in adult testis, lung, adrenal gland, gallbladder, pancreas esophagus, colon, prostate and placenta. Lastly, SPEG is highly expressed in adult heart and gallbladder, and PTK2 is in adult colon, urinary bladder and platelets.

The distribution pattern of the 29 kinases was analyzed with the FunRich database. The illustration (**Figure 4A**) depicts the distribution of gene expression percentages among various tissues such as the testis, lung, kidney, plasma, skeletal muscles, brain, heart muscle, spleen, prostate, pancreas, liver, nasopharynx, bronchus, small intestine, placenta, HUVEC (human umbilical vein endothelial cells), and colon. Among these tissues, the testis exhibits the highest gene expression level at 85.7%, whereas the nasopharynx shows the lowest at 50%. The heatmap displays (**Figure 4B**) the levels of gene expression in different fetal and adult tissues, as well as specific cell types. KALRN is significantly expressed in the adult heart and platelets, while PRKDC is highly expressed in all tissues other than platelets. Adult gallbladder and prostate exhibit comparatively increased expression levels of HGS and PRPF6. There is a noticeable expression of PRPF6 in the lung. Genes such as FAM47E, CERKL, SIK3, PFKFB4, MAP3K6, AKAP14, and MOB3C, on the other hand, have relatively low expression in the majority of tissues. PDK2 and STK24 are significantly expressed in the adult kidney, while STK24 is also present in the esophagus, colon, urinary bladder, prostate, and platelets. Notable patterns include MYLK2 being highly expressed in the adult heart, adult gallbladder, esophagus, and colon. In addition, the adult frontal cortex, spinal cord, kidney, colon, bladder, and prostate all have significant expression of HGS, while the adult testis, lung, adrenal gland, gallbladder, pancreatic esophagus, colon, prostate, and placenta all exhibit high expression of PRPF6. Finally, the adult colon, bladder, and platelets express PTK2, but the adult heart and gallbladder exhibit large levels of SPEG. These expression patterns highlight the various and tissue-specific roles of these genes, showing their contribution to maintaining physiological systems.

### 3.5 Protein domain and transcription factor regulation analysis of the kinases

**Figure 5:**
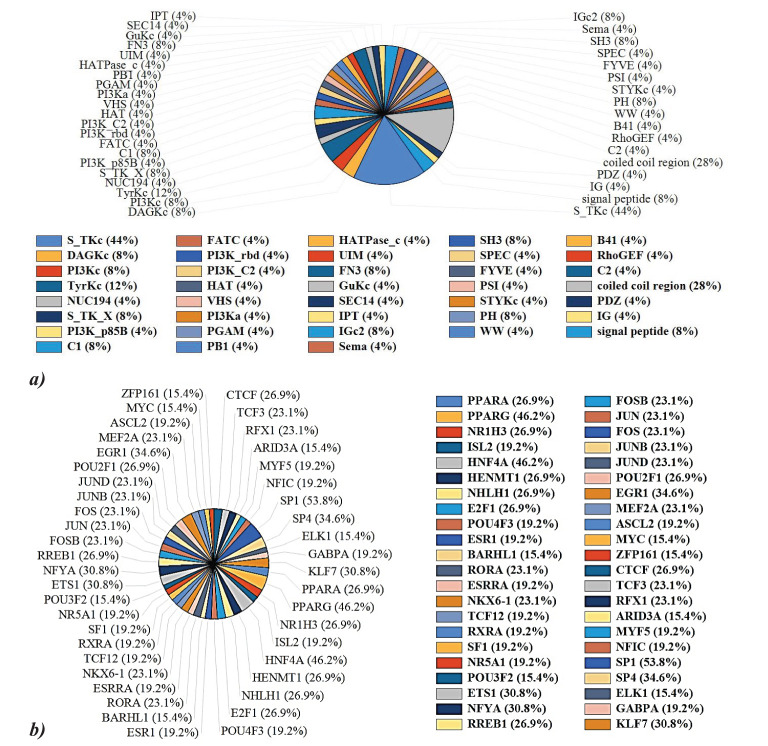
**(A)** Protein domain analysis of the key proteins reveals serine/threonine kinase domain and coiled-coil region is ubiquitously present in our query hits. Other protein domains like Tyrosine kinase C, FN3, IGc2, and SH3 domains are also observed among the kinases. **(B)** PPARG (46.2%), PPARA (26.9%), SP1 (53.8%), and HNF4A (46.2%) are predominant transcription factors regulating PPRE kinases.

FunRich was used to analyze the protein domain distribution among the 29 kinases which revealed serine/threonine kinase domain and coiled-coil region are ubiquitously present in our query hits. A significant distribution of C1, Tyrosine kinase C, FN3, IGc2, and SH3 domains is also observed among the kinases (**Figure 5A**). Coiled-coil regions (28%) facilitate structural stability and protein-protein interactions, while immunoglobulin domains (IG 4% and IGc2 8%) are crucial for immune functions. Serine/threonine protein kinases (S_TKc, 44%) demonstrate their role in signal transduction through phosphorylation. Diacylglycerol kinases (DAGk_c, 8%) and tyrosine kinases (TyrKc, 12%) highlight their importance in lipid signaling and growth factor pathways, respectively. Additional domains like SH3 (8%), PH (8%), and RhoGEF (4%) further indicate involvement in signaling, structural organization, and immune responses. Further ToppGene analysis of the hits revealed that 13 kinases from hits (BRSK2, CDK8, PRKCH, MAP3K15, PRKCZ, SIK3, KALRN, STK35, MAP3K6, CLK2, STK24, SPEG, MYLK2) possess serine/threonine kinase domain, reinforcing their central role in various signaling cascades and cellular processes.

TFs constitute a class of proteins, pivotal in the orchestration of gene expression regulation. These molecules exhibit a binding affinity towards distinct DNA sequences located within gene promoters and it has the capability to either enhance or inhibit the transcriptional activity of the associated gene. FunRich analysis of the TF regulation of the query hits revealed that 46.2% and 26.9% of the hits are reported to be regulated via PPARγ and PPARα respectively. NFYA, ETS1, EGR1, SP1, KLF7, SP4, and HNF4A are other major regulator TF of the hits (**Figure 5B**).

### 3.6 Pathway analysis, functional enrichment of the selected hits

**Figure 6:**
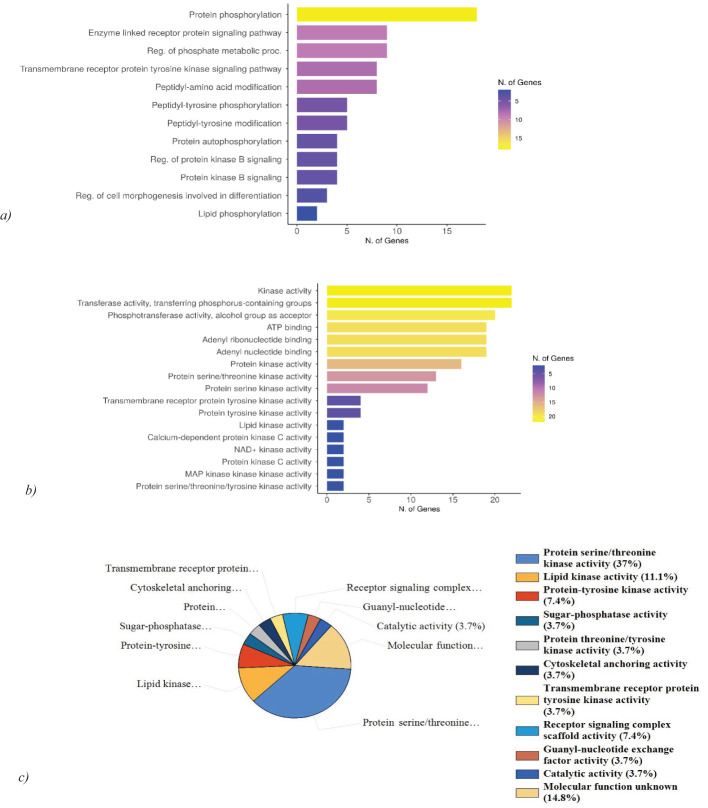
Gene ontology analysis and functional enrichment of the hits. **(A)** GO: BP analysis of hits reveals the high association of the genes with protein phosphorylation and tyrosine kinase signaling pathway. **(B)** GO: MF analysis of the hits exhibits ATP binding activity, adenyl ribonucleotide binding activity, and phosphotransferase activity. **(C)** Molecular function analysis using FunRich reveals 37% of the genes are associated with protein serine/threonine kinase activity and 11.1% possess lipid kinase activity.

The gene ontology (GO) analysis of ts revealed the relevant BP and MF, which was performed using ShinyGO 0.77. Protein hits are highly associated with protein phosphorylation and tyrosine kinase signaling pathways. The kinases predominantly exhibited ATP binding activity, and adenyl ribonucleotide binding activity (**Figures 6A, B**). This indicates that the query genes are crucial in regulating various cellular processes through phosphorylation events and signaling pathways involving tyrosine kinases. The kinases identified show notable ATP binding activity and adenyl ribonucleotide binding activity, which are essential for their role in transferring phosphate groups during phosphorylation. Further FunRich analysis of MF of the hits revealed that 37% of the query genes are involved in protein serine/threonine kinase activity, emphasizing their role in modulating cellular signaling and functions through phosphorylation of serine and threonine residues and 11.1% are involved in lipid kinase activity, highlighting their role in lipid metabolism and signaling. Other than that, the kinases show a significant association in mediating receptor signaling complex scaffold activity, and transmembrane receptor protein tyrosine kinase activity (**Figure 6C**). Gene ontology analysis was further validated using DAVID and g: Profiler (**Supplementary Figures 1, 2**). ToppGene analysis revealed BRSK2, CDK8, MET, TNK2, CERK, PRKCH, MAP3K15, PRKCZ, PRKDC, PFKFB4, SIK3, KALRN, STK35, MAP3K6, PDK2, CLK2, STK24, PIK3CD, SPEG, PTK2, MYLK2 to exhibit ATP binding activity, adenine and purine ribonucleotide binding activity. 25 kinases among the query 29 exhibit transferase activity (transferring phosphorus-containing groups), which aligns with their role in phosphorylation. PRKCH and PRKCZ exhibit calcium-dependent protein kinase C activity, important for various cellular responses, and MAGI2 beta-1 adrenergic receptor binding activity, relevant for signaling. PRKDC possesses U3 snoRNA binding activity suggesting a role in RNA processing.

Numerous biological processes are regulated by these kinases. ToppGene analysis reveals while MAGI2, PRKCZ, and KALRN are involved in the regulation of neurotransmitter receptor localization to postsynaptic specialization membrane, BRSK2, MAGI2, MET, KALRN, STK24, PIK3CD, PTK2 are involved in the regulation of neuron projection development. BRSK2, CERKL, PRKDC, PDK2, and STK24 are also involved in mediating apoptosis and oxidative stress-mediated cell death.

**Figure 7:**
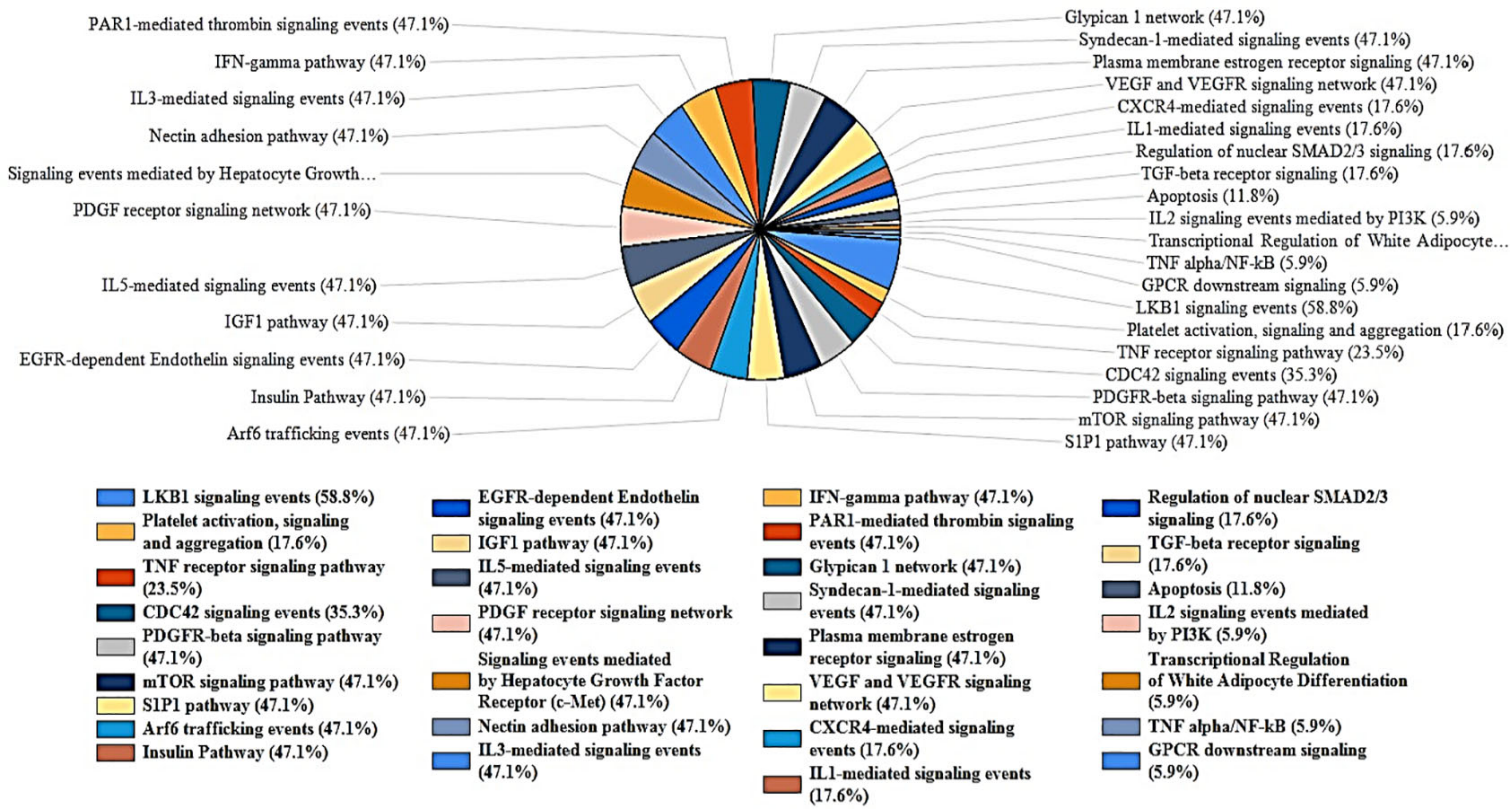
FunRich analysis of biological pathways of the key Proteins. The kinases are highly significant in mediating VEGFR, IL-3, PDGF, GMCSF, IL-5, IFN-γ, and TGF-β pathways. The hits are also associated with apoptosis, platelet activation, CDC42 signaling pathway.

Further analysis of biological pathways using FunRich revealed the hits to be predominantly involved in IL-3, IL-5, PDGF, TGF-β, IFN- γ, CDC42, TNF, and apoptotic signaling pathways (**Figure 7**). 47.1% of the genes are linked to the signaling pathways of IL-3, IL-5, IFN-γ, and PDGF. The signaling pathway for the TGF-β receptor accounts for 17.6% of the genes. Furthermore, the CDC42 signaling pathway encompasses 35.3% of the genes, while the TNF receptor signaling pathway comprises 23.5% of the genes. Finally, 11.8% of the genes are associated with apoptotic signaling pathways. ToppGene pathway analysis of the hits further revealed MET, PTK2, and HGS to be associated with the TGF-β signaling pathway. STK24 and PTK2 are predominantly involved in the apoptotic cleavage of cellular proteins. The kinases also show associations with EGFR signaling pathway (TNK2, PRKCZ, PTK2, HGS), focal adhesion (MET, TNK2, PIK3CD, PTK2, MYLK2), CXCR4 pathway (PRKCZ, PIK3CD, PTK2, HGS), and insulin signaling pathway (PRKCH, PRKCZ, MAP3K6, PIK3CD).

### 3.7 Disease-gene association of the key proteins

**Figure 8:**
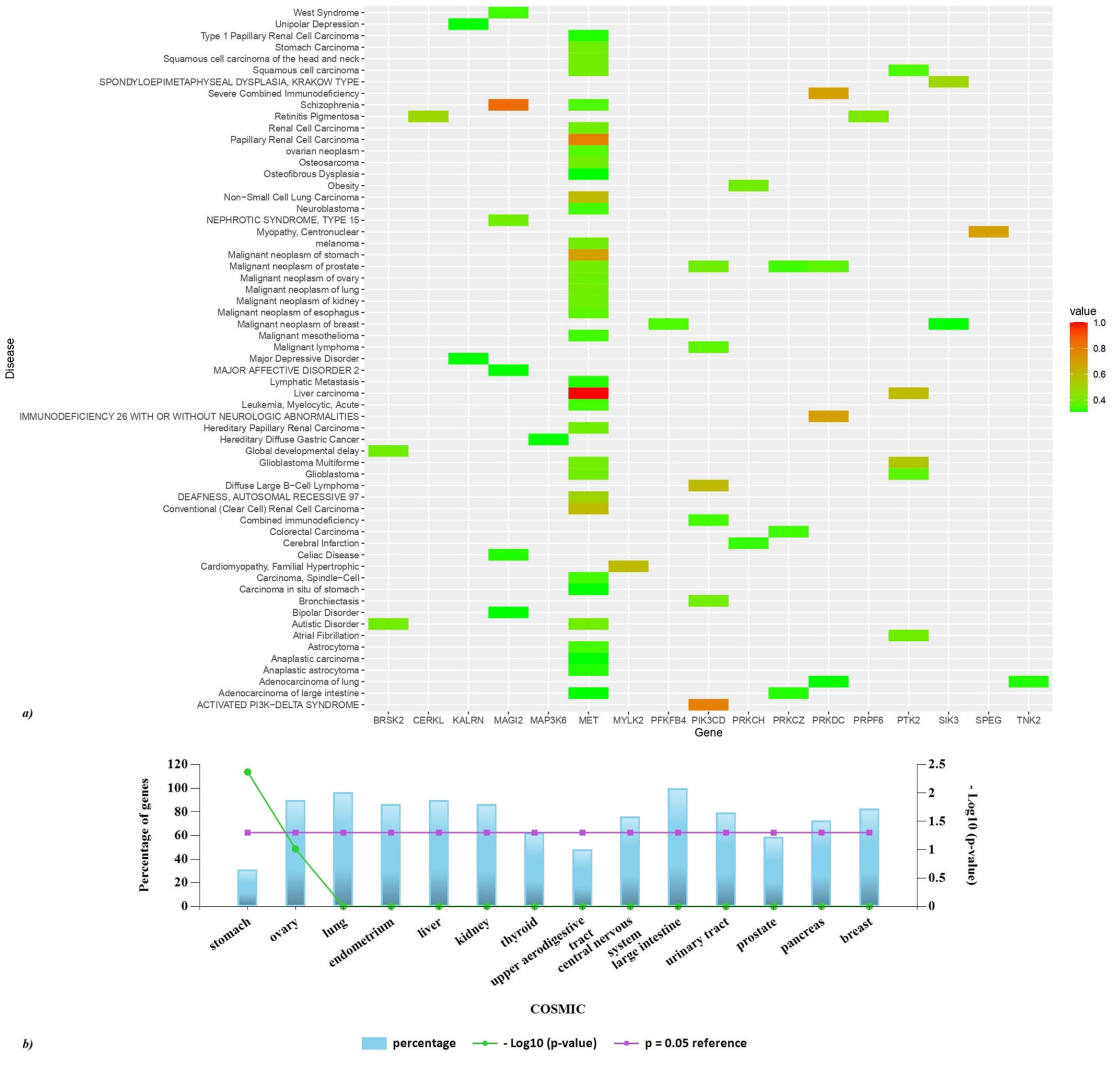
Disease association of the hits. **(A)** A heatmap of top GDA>0.3 was created using R studio. Based on GDA score a significant association of MET with liver and papillary liver cell carcinoma is observed. **(B)** FunRich analysis of the COSMIC dataset reveals the somatic mutations of the hits to be highly associated with cancer of the large intestine, lung, central nervous system, kidney, liver, breast, ovary, pancreas, urinary tract, and thyroid.

Disease association of the hits using the DisGeNET dataset was performed and a heatmap (GDA>0.3) was created using R studio (**Figure 8A**). MET is significantly associated with liver carcinoma and papillary renal cell carcinoma. MAGI2 exhibits a strong association with schizophrenia, it is also involved in celiac disease, bipolar disorder, nephrotic syndrome type 15, and west syndrome. PIK3CD is implicated in the activated PI3K-delta syndrome, a pathological condition that adversely affects the immune system’s functionality. Furthermore, this gene is correlated with bronchiectasis, malignant neoplasms of the gastric region, and malignant lymphoma. The PRKDC gene is associated with immunodeficiency 26, which may occur with or without neurologic impairments, as well as with severe combined immunodeficiency. Moreover, SPEG is linked to centronuclear myopathy, while PRKCZ is associated with adenocarcinoma of the large intestine. FunRich analysis of the COSMIC dataset reveals the somatic mutations of the hits to be highly associated with cancer of the large intestine, lung, central nervous system, kidney, liver, breast, and ovary (**Figure 8B**).

## 4 Significance

In this investigation, a systematic screening of PPREs was conducted across the entire human genome, encompassing both the positive and negative DNA strands facilitated by a comprehensive search using NCBI Genome Workbench. Subsequently, employing the UCSC Table Browser the list of genes associated with PPREs was extracted.

Our results affirm the widespread occurrence of PPREs across the entirety of the human genome, with a substantial proportion of the identified genes likely subject to regulation by PPARs (**Supplementary Data Sheet 1**). Recognizing the implicated role of kinases in degenerative diseases, a refined analysis was conducted by screening genes located within a genomic region spanning 10 kilobases upstream to 20 kilobases downstream of the reported PPREs. This targeted approach led to the identification of 29 candidate kinases, which hold promise as potential PPAR stress targets. Subsequent to this, an examination of the TFs associated with the identified key kinases revealed significant regulatory links between the kinases and PPARγ and PPARα, indicating their crucial involvement in metabolic processes, lipid metabolism, insulin sensitivity, and inflammation. PPARγ’s high regulation suggests that many hits are linked to adipogenesis and inflammatory responses, while PPARα’s involvement points to roles in fatty acid oxidation and glucose metabolism (6–8). Additionally, NFYA, ETS1, EGR1, SP1, KLF7, SP4, and HNF4A were identified as major regulatory TFs influencing these kinase hits. NFYA is associated with cell cycle regulation and DNA repair (26, 27); ETS1 with cell growth, vascular inflammation, and immune responses (28, 29); EGR1 with cellular stress responses, apoptosis, and development (30, 31); SP1 with cell proliferation, and metastasis influence transcription of genes associated with fatty acid metabolism (32, 33); KLF7 with neuronal development and proliferation of preadipocytes (34, 35); SP4 with neuronal differentiation (36); and HNF4A with liver function and glucose metabolism (37, 38). This diverse regulation profile suggests that the hits are involved in a complex network of metabolic, developmental, and cellular processes, reflecting their significant role in maintaining cellular and metabolic homeostasis. To further elucidate their potential biological significance, we analyzed the expression patterns of these shortlisted kinases across a spectrum of human tissues. The elevated levels of particular kinases in designated tissues, including the adult heart, kidney, and prostate, imply their participation in tissue-specific physiological activities. The pronounced expression of PRKDC throughout diverse tissues points to its possible involvement in extensive cellular functions, while the notable expression of MYLK2 is specifically detected in the adult heart and colon. Additionally, an exploration of the interactions between these kinases and the human Protein-Protein Interaction (PPI) network revealed that they interacted with nearly 5.56% of the total human PPIs. This suggests a widespread influence of these kinases on cellular protein interactions. The identification of key hub genes, such as PRKDC, PRKCZ, and MET, which are central to multiple aspects of PPI networks, further emphasizes the pivotal role these kinases may play in maintaining cellular homeostasis and responding to environmental cues. Furthermore, through functional enrichment analysis and disease association annotation, we demonstrated the significant contribution of PPAR-mediated signaling in the pathophysiology of a broad spectrum of disease categories. The correlation of these kinases with disease-related pathways, particularly their participation in oncological and immunological disorders, underscores their potential as viable therapeutic targets. The identification of noteworthy disease associations, including hepatocellular carcinoma, schizophrenia, and immunodeficiency syndromes, accentuates the clinical significance of these kinases. Pathway analysis underscores the involvement of these kinases in critical signaling pathways, such as those mediated by IL-3, IL-5, PDGF, TGF-β, and IFN-γ, which are essential for immune responses, cell growth, and apoptosis. Additionally, the engagement of these kinases in apoptosis, platelet activation, and CDC42 signaling illuminates the multifaceted role of PPAR in the regulation of cellular homeostasis and biological processes. The participation of genes in the IL-3, IL-5, and IFN-γ signaling cascades underscores their critical roles in immune functionality, especially in the modulation of cellular proliferation, differentiation, and the response to inflammatory stimuli (39, 40). The correlation with the PDGF and TGF-β receptor pathways signifies the genes’ contributions to essential processes such as cellular growth, migration, and differentiation, which are imperative in the contexts of wound healing, fibrosis, and the advancement of cancer (41–43). The involvement of the CDC42 signaling pathway accentuates the genes’ functions in preserving cellular morphology, migration, and signaling mechanisms, which are vital for the efficacy of immune cells and the metastasis of cancer (44–48). The relationship with the TNF receptor signaling pathway highlights their prominence in the realms of inflammation and apoptosis, thereby further corroborating their significance in immune responses and the regulation of cellular death (49, 50). Finally, the genes’ association with apoptotic signaling pathways emphasizes their involvement in programmed cell death, which is essential for the prevention of uncontrolled cellular proliferation and the maintenance of cellular homeostasis, factors that are crucial in cancer prevention (51, 52).

## 5 Conclusion

Cellular stress responses represent integral processes in cellular physiology. Over the course of evolution, multicellular organisms have developed an array of cellular resilience mechanisms, with the outcome of these mechanisms being dependent on the nature of the stressors, cell types involved, and prevailing environmental conditions. PPARs’ involvement across a spectrum of disorders, notably including cancer, neurodegenerative diseases, diabetes, and pulmonary fibrosis, designates them as prime targets for therapeutic interventions. In the context of this study, we have discerned 29 PPRE-associated kinases from the genes associated with PPREs, which were obtained through the UCSC Table Browser. Our –investigation has unveiled 660 genes (29 kinases) within the human chromosome potentially subject to regulation by PPARs, thereby significantly augmenting the existing knowledge regarding PPAR target genes. The analyses of functional enrichment and pathways highlight the crucial role that these kinases have in regulating cellular and metabolic functions, which has profound implications for comprehending the pathophysiology of various diseases, such as cancer, cardiovascular issues, and immune system malfunctions. The link between these kinases and key disease pathways, especially within oncological and immunological frameworks, emphasizes their potential as targets for therapeutic intervention. This expanded understanding holds the promise of novel therapeutic approaches by establishing a firm groundwork for targeting downstream molecular mechanisms.

## Data Availability

The raw data supporting the conclusions of this article will be made available by the authors, without undue reservation.
