# Supplementary material for: Identification of PPREs and PPRE associated genes in the human genome: insights into related kinases and disease implications
Source: Front Immunol. 2024 Oct 2;15:1457648. doi: 10.3389/fimmu.2024.1457648 (PMC11491715; doi:10.3389/fimmu.2024.1457648)
Supplement: Supplementary file 1 [file DataSheet1.docx]

**Supplementary Table 1: Location of PPREs and associated genes in the positive strand of human chromosome.**

| **Positive Strand** | | | |
| --- | --- | --- | --- |
| **Chromosome Number** | **PPRE Sequence** | **No. of PPRE response element** | **No. of genes present** |
| Chr 1 | AGGTCAAAGGTCA | 10 | 22 |
|  | AGGTCACAGGTCA | 5 |  |
|  | AGGTCAGAGGTCA | 15 |  |
|  | AGGTCATAGGTCA | 5 |  |
| Chr 2 | AGGTCAAAGGTCA | 7 | 17 |
|  | AGGTCACAGGTCA | 7 |  |
|  | AGGTCAGAGGTCA | 15 |  |
|  | AGGTCATAGGTCA | 1 |  |
| Chr 3 | AGGTCAAAGGTCA | 4 | 26 |
|  | AGGTCACAGGTCA | 7 |  |
|  | AGGTCAGAGGTCA | 14 |  |
|  | AGGTCATAGGTCA | 4 |  |
| Chr 4 | AGGTCAAAGGTCA | 4 | 11 |
|  | AGGTCACAGGTCA | 4 |  |
|  | AGGTCAGAGGTCA | 8 |  |
|  | AGGTCATAGGTCA | 7 |  |
| Chr 5 | AGGTCAAAGGTCA | 6 | 10 |
|  | AGGTCACAGGTCA | 2 |  |
|  | AGGTCAGAGGTCA | 17 |  |
|  | AGGTCATAGGTCA | 3 |  |
| Chr 6 | AGGTCAAAGGTCA | 11 | 10 |
|  | AGGTCACAGGTCA | 1 |  |
|  | AGGTCAGAGGTCA | 7 |  |
|  | AGGTCATAGGTCA | 2 |  |
| Chr 7 | AGGTCAAAGGTCA | 7 | 11 |
|  | AGGTCACAGGTCA | 4 |  |
|  | AGGTCAGAGGTCA | 7 |  |
|  | AGGTCATAGGTCA | 5 |  |
| Chr 8 | AGGTCAAAGGTCA | 8 | 9 |
|  | AGGTCACAGGTCA | 7 |  |
|  | AGGTCAGAGGTCA | 3 |  |
|  | AGGTCATAGGTCA | 2 |  |
| Chr 9 | AGGTCAAAGGTCA | 4 | 14 |
|  | AGGTCACAGGTCA | 4 |  |
|  | AGGTCAGAGGTCA | 9 |  |
|  | AGGTCATAGGTCA | 1 |  |
| Chr 10 | AGGTCAAAGGTCA | 6 | 18 |
|  | AGGTCACAGGTCA | 8 |  |
|  | AGGTCAGAGGTCA | 13 |  |
|  | AGGTCATAGGTCA | 6 |  |
| Chr 11 | AGGTCAAAGGTCA | 6 | 20 |
|  | AGGTCACAGGTCA | 2 |  |
|  | AGGTCAGAGGTCA | 15 |  |
|  | AGGTCATAGGTCA | 2 |  |
| Chr 12 | AGGTCAAAGGTCA | 5 | 12 |
|  | AGGTCACAGGTCA | 4 |  |
|  | AGGTCAGAGGTCA | 9 |  |
|  | AGGTCATAGGTCA | 5 |  |
| Chr 13 | AGGTCAAAGGTCA | 4 | 8 |
|  | AGGTCACAGGTCA | 3 |  |
|  | AGGTCAGAGGTCA | 5 |  |
|  | AGGTCATAGGTCA | 3 |  |
| Chr 14 | AGGTCAAAGGTCA | 5 | 12 |
|  | AGGTCACAGGTCA | 4 |  |
|  | AGGTCAGAGGTCA | 7 |  |
|  | AGGTCATAGGTCA | 2 |  |
| Chr 15 | AGGTCAAAGGTCA | 4 | 8 |
|  | AGGTCACAGGTCA | 5 |  |
|  | AGGTCAGAGGTCA | 6 |  |
|  | AGGTCATAGGTCA | 6 |  |
| Chr 16 | AGGTCAAAGGTCA | 3 | 26 |
|  | AGGTCACAGGTCA | 3 |  |
|  | AGGTCAGAGGTCA | 17 |  |
|  | AGGTCATAGGTCA | 3 |  |
| Chr 17 | AGGTCAAAGGTCA | 5 | 27 |
|  | AGGTCACAGGTCA | 4 |  |
|  | AGGTCAGAGGTCA | 12 |  |
|  | AGGTCATAGGTCA | 5 |  |
| Chr 18 | AGGTCAAAGGTCA |  | 3 |
|  | AGGTCACAGGTCA | 1 |  |
|  | AGGTCAGAGGTCA | 4 |  |
|  | AGGTCATAGGTCA | 2 |  |
| Chr 19 | AGGTCAAAGGTCA | 3 | 15 |
|  | AGGTCACAGGTCA |  |  |
|  | AGGTCAGAGGTCA | 17 |  |
|  | AGGTCATAGGTCA | 3 |  |
| Chr 20 | AGGTCAAAGGTCA | 3 | 13 |
|  | AGGTCACAGGTCA | 1 |  |
|  | AGGTCAGAGGTCA | 11 |  |
|  | AGGTCATAGGTCA | 1 |  |
| Chr 21 | AGGTCAAAGGTCA | 1 | 5 |
|  | AGGTCACAGGTCA | 2 |  |
|  | AGGTCAGAGGTCA | 3 |  |
|  | AGGTCATAGGTCA | 2 |  |
| Chr 22 | AGGTCAAAGGTCA | 3 | 5 |
|  | AGGTCACAGGTCA |  |  |
|  | AGGTCAGAGGTCA | 4 |  |
|  | AGGTCATAGGTCA | 1 |  |
| Chr X | AGGTCAAAGGTCA | 4 | 6 |
|  | AGGTCACAGGTCA | 1 |  |
|  | AGGTCAGAGGTCA | 7 |  |
|  | AGGTCATAGGTCA | 2 |  |
| Chr Y | AGGTCAAAGGTCA | 2 | 3 |
|  | AGGTCACAGGTCA | 1 |  |
|  | AGGTCAGAGGTCA | 1 |  |
|  | AGGTCATAGGTCA |  |  |

**Supplementary Table 2: Location of PPREs and associated genes in the negative strand of human chromosome.**

| **Negative Strand** | | | |
| --- | --- | --- | --- |
| **Chromosome Number** | **PPRE Sequence** | **No. of PPRE response element** | **No. of genes present** |
| Chr 1 | AGGTCAAAGGTCA | 10 | 41 |
|  | AGGTCACAGGTCA | 15 |  |
|  | AGGTCAGAGGTCA | 23 |  |
|  | AGGTCATAGGTCA | 9 |  |
| Chr 2 | AGGTCAAAGGTCA | 8 | 17 |
|  | AGGTCACAGGTCA | 8 |  |
|  | AGGTCAGAGGTCA | 13 |  |
|  | AGGTCATAGGTCA | 6 |  |
| Chr 3 | AGGTCAAAGGTCA | 8 | 28 |
|  | AGGTCACAGGTCA | 5 |  |
|  | AGGTCAGAGGTCA | 11 |  |
|  | AGGTCATAGGTCA | 4 |  |
| Chr 4 | AGGTCAAAGGTCA | 7 | 14 |
|  | AGGTCACAGGTCA | 2 |  |
|  | AGGTCAGAGGTCA | 10 |  |
|  | AGGTCATAGGTCA | 4 |  |
| Chr 5 | AGGTCAAAGGTCA | 6 | 12 |
|  | AGGTCACAGGTCA | 3 |  |
|  | AGGTCAGAGGTCA | 8 |  |
|  | AGGTCATAGGTCA | 4 |  |
| Chr 6 | AGGTCAAAGGTCA | 7 | 15 |
|  | AGGTCACAGGTCA | 4 |  |
|  | AGGTCAGAGGTCA | 5 |  |
|  | AGGTCATAGGTCA | 3 |  |
| Chr 7 | AGGTCAAAGGTCA | 4 | 7 |
|  | AGGTCACAGGTCA | 4 |  |
|  | AGGTCAGAGGTCA | 9 |  |
|  | AGGTCATAGGTCA | 2 |  |
| Chr 8 | AGGTCAAAGGTCA | 7 | 21 |
|  | AGGTCACAGGTCA | 8 |  |
|  | AGGTCAGAGGTCA | 7 |  |
|  | AGGTCATAGGTCA | 2 |  |
| Chr 9 | AGGTCAAAGGTCA | 2 | 18 |
|  | AGGTCACAGGTCA | 5 |  |
|  | AGGTCAGAGGTCA | 15 |  |
|  | AGGTCATAGGTCA | 2 |  |
| Chr 10 | AGGTCAAAGGTCA | 5 | 10 |
|  | AGGTCACAGGTCA | 10 |  |
|  | AGGTCAGAGGTCA | 9 |  |
|  | AGGTCATAGGTCA |  |  |
| Chr 11 | AGGTCAAAGGTCA | 11 | 13 |
|  | AGGTCACAGGTCA | 7 |  |
|  | AGGTCAGAGGTCA | 12 |  |
|  | AGGTCATAGGTCA | 4 |  |
| Chr 12 | AGGTCAAAGGTCA | 8 | 13 |
|  | AGGTCACAGGTCA | 6 |  |
|  | AGGTCAGAGGTCA | 4 |  |
|  | AGGTCATAGGTCA | 1 |  |
| Chr 13 | AGGTCAAAGGTCA | 8 | 9 |
|  | AGGTCACAGGTCA | 3 |  |
|  | AGGTCAGAGGTCA | 3 |  |
|  | AGGTCATAGGTCA |  |  |
| Chr 14 | AGGTCAAAGGTCA | 3 | 11 |
|  | AGGTCACAGGTCA | 4 |  |
|  | AGGTCAGAGGTCA | 8 |  |
|  | AGGTCATAGGTCA | 2 |  |
| Chr 15 | AGGTCAAAGGTCA | 2 | 9 |
|  | AGGTCACAGGTCA | 3 |  |
|  | AGGTCAGAGGTCA | 10 |  |
|  | AGGTCATAGGTCA | 2 |  |
| Chr 16 | AGGTCAAAGGTCA | 2 | 6 |
|  | AGGTCACAGGTCA | 4 |  |
|  | AGGTCAGAGGTCA | 9 |  |
|  | AGGTCATAGGTCA |  |  |
| Chr 17 | AGGTCAAAGGTCA | 5 | 32 |
|  | AGGTCACAGGTCA |  |  |
|  | AGGTCAGAGGTCA | 18 |  |
|  | AGGTCATAGGTCA | 1 |  |
| Chr 18 | AGGTCAAAGGTCA | 2 | 5 |
|  | AGGTCACAGGTCA | 2 |  |
|  | AGGTCAGAGGTCA | 4 |  |
|  | AGGTCATAGGTCA | 2 |  |
| Chr 19 | AGGTCAAAGGTCA | 8 | 30 |
|  | AGGTCACAGGTCA | 9 |  |
|  | AGGTCAGAGGTCA | 22 |  |
|  | AGGTCATAGGTCA | 3 |  |
| Chr 20 | AGGTCAAAGGTCA | 5 | 18 |
|  | AGGTCACAGGTCA | 2 |  |
|  | AGGTCAGAGGTCA | 9 |  |
|  | AGGTCATAGGTCA | 1 |  |
| Chr 21 | AGGTCAAAGGTCA |  | 2 |
|  | AGGTCACAGGTCA |  |  |
|  | AGGTCAGAGGTCA | 3 |  |
|  | AGGTCATAGGTCA |  |  |
| Chr 22 | AGGTCAAAGGTCA | 1 | 9 |
|  | AGGTCACAGGTCA | 6 |  |
|  | AGGTCAGAGGTCA | 4 |  |
|  | AGGTCATAGGTCA |  |  |
| Chr X | AGGTCAAAGGTCA | 3 | 6 |
|  | AGGTCACAGGTCA | 3 |  |
|  | AGGTCAGAGGTCA | 7 |  |
|  | AGGTCATAGGTCA | 3 |  |
| Chr Y | AGGTCAAAGGTCA | 4 | 3 |
|  | AGGTCACAGGTCA | 1 |  |
|  | AGGTCAGAGGTCA | 2 |  |
|  | AGGTCATAGGTCA |  |  |

**Supplementary Table 3: The high confidence interactions (confidence score ≥ 0.7) of 29 kinases in** **the HIPPIE- Human Scored Interactions database.**

| **Source Node** | **Target Node** | **Confidence Value** | **Interaction Detection Methods** |
| --- | --- | --- | --- |
| AAR2 | PRPF6 | 0.8 | MI:0004(affinity chromatography technology) |
| ACAD9 | PDK2 | 0.87 | MI:0004(affinity chromatography technology)\|MI:0007(anti tag coimmunoprecipitation)\|bioid |
| ADAP1 | PRKCZ | 0.89 | MI:0492(in vitro)\|Affinity Capture-Western\|Reconstituted Complex\|Biochemical Activity\|MI:0415(enzymatic study)\|MI:0004(affinity chromatography technology)\|MI:0096(pull down) |
| ADCK2 | PDK2 | 0.72 | MI:0004(affinity chromatography technology)\|MI:0007(anti tag coimmunoprecipitation) |
| ADRB2 | HGS | 0.75 | MI:0004(affinity chromatography technology)\|MI:0428(imaging technique) |
| AES | HGS | 0.87 | MI:0004(affinity chromatography technology)\|MI:0018(two hybrid)\|MI:0401(biochemical) |
| AGR2 | PRKDC | 0.72 | MI:0004(affinity chromatography technology)\|bioid |
| AKAP14 | PRKAR2A | 0.9 | MI:0492(in vitro)\|MI:0493(in vivo)\|Affinity Capture-Western\|Reconstituted Complex\|MI:0004(affinity chromatography technology)\|MI:0096(pull down)\|MI:0018(two hybrid)\|MI:0007(anti tag coimmunoprecipitation) |
| AKAP14 | PRKAR2B | 0.88 | MI:0492(in vitro)\|MI:0493(in vivo)\|MI:0004(affinity chromatography technology)\|MI:0007(anti tag coimmunoprecipitation)\|MI:0018(two hybrid) |
| AKAP14 | ARL5B | 0.82 | MI:0004(affinity chromatography technology)\|MI:0007(anti tag coimmunoprecipitation) |
| AKAP14 | EFCAB7 | 0.83 | MI:0004(affinity chromatography technology)\|MI:0007(anti tag coimmunoprecipitation) |
| AKAP14 | SYNJ2BP | 0.83 | MI:0004(affinity chromatography technology)\|MI:0007(anti tag coimmunoprecipitation) |
| AKAP14 | OCIAD1 | 0.83 | MI:0004(affinity chromatography technology)\|MI:0007(anti tag coimmunoprecipitation) |
| AKAP14 | SPA17 | 0.83 | MI:0004(affinity chromatography technology)\|MI:0007(anti tag coimmunoprecipitation) |
| AKAP14 | PRKACG | 0.83 | MI:0004(affinity chromatography technology)\|MI:0007(anti tag coimmunoprecipitation) |
| AKAP14 | PRKACB | 0.83 | MI:0004(affinity chromatography technology)\|MI:0007(anti tag coimmunoprecipitation) |
| AKT1 | PRKDC | 0.88 | MI:0492(in vitro)\|MI:0415(enzymatic study)\|MI:0004(affinity chromatography technology)\|MI:0096(pull down) |
| ANXA2 | PTK2 | 0.72 | MI:0030(cross-linking study)\|bioid |
| APOBEC3D | CLK2 | 0.72 | MI:0004(affinity chromatography technology)\|MI:0007(anti tag coimmunoprecipitation) |
| ARAF | PRKDC | 0.73 | MI:0004(affinity chromatography technology) |
| ARHGAP26 | PTK2 | 0.76 | MI:0492(in vitro)\|MI:0493(in vivo)\|Reconstituted Complex\|MI:0096(pull down) |
| ARMC8 | HGS | 0.72 | MI:0004(affinity chromatography technology)\|MI:0018(two hybrid) |
| ARRB1 | HGS | 0.82 | MI:0004(affinity chromatography technology)\|MI:0007(anti tag coimmunoprecipitation) |
| ARRDC3 | HGS | 0.83 | MI:0416(fluorescence microscopy)\|MI:0007(anti tag coimmunoprecipitation)\|MI:0004(affinity chromatography technology) |
| ASAP1 | PTK2 | 0.96 | MI:0492(in vitro)\|MI:0493(in vivo)\|MI:0018(two hybrid)\|MI:0004(affinity chromatography technology)\|MI:0114(x-ray crystallography)\|bioid |
| ATG101 | PRKDC | 0.72 | MI:0007(anti tag coimmunoprecipitation)\|MI:0004(affinity chromatography technology) |
| ATG4C | PRKDC | 0.72 | MI:0007(anti tag coimmunoprecipitation)\|MI:0004(affinity chromatography technology) |
| ATM | PRKDC | 0.86 | MI:0492(in vitro)\|Protein-peptide\|Affinity Capture-Western\|MI:0004(affinity chromatography technology) |
| ATP6V1C2 | STK24 | 0.83 | MI:0004(affinity chromatography technology)\|MI:0007(anti tag coimmunoprecipitation) |
| ATR | CLK2 | 0.73 | MI:0004(affinity chromatography technology) |
| AURKB | PRKDC | 0.78 | MI:0096(pull down)\|MI:0004(affinity chromatography technology) |
| AVPR2 | HGS | 0.75 | MI:0004(affinity chromatography technology)\|MI:0428(imaging technique) |
| BAD | PRKDC | 0.72 | MI:0007(anti tag coimmunoprecipitation)\|MI:0004(affinity chromatography technology) |
| BAG3 | TNK2 | 0.84 | MI:0096(pull down)\|MI:0004(affinity chromatography technology)\|MI:0007(anti tag coimmunoprecipitation) |
| BCAR3 | MET | 0.75 | MI:0053(fluorescence polarization spectroscopy)\|MI:0096(pull down) |
| BECN1 | PRKDC | 0.72 | MI:0007(anti tag coimmunoprecipitation)\|MI:0004(affinity chromatography technology) |
| BIN1 | PTK2 | 0.73 | MI:0492(in vitro)\|Affinity Capture-Western\|MI:0004(affinity chromatography technology) |
| BIRC5 | PRKDC | 0.73 | MI:0004(affinity chromatography technology) |
| BLK | MET | 0.75 | MI:0053(fluorescence polarization spectroscopy)\|MI:0096(pull down) |
| BRD4 | PRPF6 | 0.73 | MI:0004(affinity chromatography technology) |
| BRD4 | PRKDC | 0.73 | MI:0004(affinity chromatography technology) |
| BSG | PRPF6 | 0.83 | MI:0004(affinity chromatography technology)\|MI:0007(anti tag coimmunoprecipitation) |
| C12orf65 | MYLK2 | 0.83 | MI:0004(affinity chromatography technology)\|MI:0007(anti tag coimmunoprecipitation) |
| C1D | PRKDC | 0.79 | MI:0492(in vitro)\|MI:0018(two hybrid)\|Reconstituted Complex\|Biochemical Activity\|Affinity Capture-Western\|MI:0415(enzymatic study)\|MI:0004(affinity chromatography technology)\|MI:0096(pull down) |
| C1QBP | PRKCZ | 0.97 | MI:0492(in vitro)\|MI:0493(in vivo)\|Reconstituted Complex\|Affinity Capture-Western\|MI:0004(affinity chromatography technology)\|MI:0096(pull down)\|MI:0006(anti bait coimmunoprecipitation)\|MI:0007(anti tag coimmunoprecipitation) |
| C4orf19 | STK24 | 0.83 | MI:0004(affinity chromatography technology)\|MI:0007(anti tag coimmunoprecipitation) |
| CABCOCO1 | MOB3C | 0.73 | MI:0004(affinity chromatography technology) |
| CALM1 | MYLK2 | 0.83 | MI:0055(fluorescent resonance energy transfer)\|MI:0004(affinity chromatography technology) |
| CAMKK2 | PRKDC | 0.72 | MI:0007(anti tag coimmunoprecipitation)\|MI:0004(affinity chromatography technology) |
| CASP2 | PRKDC | 0.71 | MI:0027(cosedimentation)\|MI:0006(anti bait coimmunoprecipitation)\|MI:0096(pull down) |
| CBL | MET | 0.9 | MI:0492(in vitro)\|MI:0493(in vivo)\|MI:0018(two hybrid)\|Biochemical Activity\|Affinity Capture-Western\|Co-crystal Structure\|MI:0006(anti bait coimmunoprecipitation)\|MI:0019(coimmunoprecipitation)\|MI:0114(x-ray crystallography)\|MI:0045(experimental interaction detection)\|MI:0065(isothermal titration calorimetry)\|MI:0415(enzymatic study)\|MI:0004(affinity chromatography technology)\|MI:0096(pull down)\|MI:0107(surface plasmon resonance)\|MI:0069(mass spectrometry studies of complexes)\|MI:0047(far western blotting)\|MI:0428(imaging technique) |
| CBL | PTK2 | 0.76 | MI:0428(imaging technique)\|MI:0004(affinity chromatography technology) |
| CBLB | PTK2 | 0.73 | MI:0004(affinity chromatography technology) |
| CCNA1 | PTK2 | 0.75 | MI:0006(anti bait coimmunoprecipitation)\|MI:0428(imaging technique) |
| CCNDBP1 | CERK | 0.83 | MI:0004(affinity chromatography technology)\|MI:0007(anti tag coimmunoprecipitation) |
| CCR5 | PTK2 | 0.74 | MI:0492(in vitro)\|MI:0493(in vivo)\|Affinity Capture-Western\|MI:0004(affinity chromatography technology) |
| CD2BP2 | PRPF6 | 0.9 | Affinity Capture-MS\|MI:0006(anti bait coimmunoprecipitation)\|MI:0018(two hybrid)\|MI:0059(gst pull down)\|MI:0004(affinity chromatography technology)\|MI:0096(pull down)\|MI:0007(anti tag coimmunoprecipitation)\|MI:0676(tandem affinity purification)\|MI:0401(biochemical) |
| CD44 | MET | 0.84 | MI:0030(cross-linking study)\|MI:0055(fluorescent resonance energy transfer) |
| CD44 | PRKCZ | 0.82 | MI:0004(affinity chromatography technology)\|MI:0007(anti tag coimmunoprecipitation) |
| CD83 | CERK | 0.82 | MI:0004(affinity chromatography technology)\|MI:0007(anti tag coimmunoprecipitation) |
| CDC5L | PRKDC | 0.88 | Affinity Capture-MS\|MI:0025(copurification)\|MI:0004(affinity chromatography technology)\|MI:0006(anti bait coimmunoprecipitation)\|MI:0401(biochemical) |
| CDC5L | STK24 | 0.72 | MI:0006(anti bait coimmunoprecipitation)\|MI:0004(affinity chromatography technology) |
| CDC5L | PRPF6 | 0.86 | MI:0006(anti bait coimmunoprecipitation)\|MI:0004(affinity chromatography technology)\|bioid |
| CDC5L | KALRN | 0.82 | MI:0018(two hybrid)\|MI:0399(two hybrid fragment pooling approach) |
| CDH1 | MET | 0.72 | Affinity Capture-Western\|MI:0004(affinity chromatography technology) |
| CDK19 | CDK8 | 0.8 | MI:0226(ion exchange chromatography)\|MI:0004(affinity chromatography technology) |
| CDK2 | PRKDC | 0.73 | MI:0004(affinity chromatography technology) |
| CDK8 | MED12 | 0.9 | MI:0493(in vivo)\|Affinity Capture-MS\|Affinity Capture-Western\|MI:0004(affinity chromatography technology)\|MI:0676(tandem affinity purification)\|MI:0096(pull down)\|MI:0007(anti tag coimmunoprecipitation)\|MI:0006(anti bait coimmunoprecipitation)\|bioid\|MI:0401(biochemical) |
| CDK8 | CCNC | 1 | MI:0492(in vitro)\|MI:0493(in vivo)\|Affinity Capture-Western\|Co-fractionation\|Reconstituted Complex\|MI:0006(anti bait coimmunoprecipitation)\|MI:0096(pull down)\|MI:0424(protein kinase assay)\|MI:0004(affinity chromatography technology)\|MI:0055(fluorescent resonance energy transfer)\|MI:0114(x-ray crystallography)\|MI:0364(inferred by curator)\|MI:0676(tandem affinity purification)\|MI:0018(two hybrid)\|MI:0091(chromatography technology)\|MI:0071(molecular sieving)\|MI:0007(anti tag coimmunoprecipitation)\|MI:0012(bioluminescence resonance energy transfer)\|bioid\|MI:0401(biochemical) |
| CDK8 | CEBPB | 0.72 | Affinity Capture-Western\|MI:0004(affinity chromatography technology) |
| CDK8 | KAT2B | 0.93 | Affinity Capture-Western\|MI:0004(affinity chromatography technology)\|bioid |
| CDK8 | SMARCB1 | 0.77 | Affinity Capture-Western\|Co-fractionation\|MI:0004(affinity chromatography technology)\|MI:0401(biochemical) |
| CDK8 | MED23 | 0.9 | Affinity Capture-Western\|MI:0004(affinity chromatography technology)\|MI:0676(tandem affinity purification)\|MI:0007(anti tag coimmunoprecipitation)\|MI:0096(pull down)\|MI:0006(anti bait coimmunoprecipitation)\|bioid |
| CDK8 | MED14 | 0.9 | Affinity Capture-Western\|Affinity Capture-MS\|MI:0004(affinity chromatography technology)\|MI:0676(tandem affinity purification)\|MI:0007(anti tag coimmunoprecipitation)\|MI:0096(pull down)\|MI:0006(anti bait coimmunoprecipitation)\|bioid\|MI:0401(biochemical) |
| CDK8 | MED13 | 0.9 | Affinity Capture-MS\|Affinity Capture-Western\|MI:0004(affinity chromatography technology)\|MI:0676(tandem affinity purification)\|MI:0096(pull down)\|MI:0007(anti tag coimmunoprecipitation)\|MI:0006(anti bait coimmunoprecipitation)\|bioid |
| CDK8 | MED1 | 0.9 | Affinity Capture-MS\|Affinity Capture-Western\|MI:0004(affinity chromatography technology)\|MI:0226(ion exchange chromatography)\|MI:0006(anti bait coimmunoprecipitation)\|MI:0676(tandem affinity purification)\|MI:0007(anti tag coimmunoprecipitation)\|MI:0096(pull down)\|bioid\|MI:0401(biochemical) |
| CDK8 | THRAP3 | 0.72 | Affinity Capture-MS\|MI:0004(affinity chromatography technology) |
| CDK8 | MED24 | 0.9 | Affinity Capture-MS\|Affinity Capture-Western\|MI:0004(affinity chromatography technology)\|MI:0676(tandem affinity purification)\|MI:0007(anti tag coimmunoprecipitation)\|MI:0096(pull down)\|MI:0006(anti bait coimmunoprecipitation)\|bioid\|MI:0401(biochemical) |
| CDK8 | MED16 | 0.9 | Affinity Capture-MS\|Affinity Capture-Western\|MI:0004(affinity chromatography technology)\|MI:0676(tandem affinity purification)\|MI:0007(anti tag coimmunoprecipitation)\|MI:0096(pull down)\|MI:0006(anti bait coimmunoprecipitation)\|bioid |
| CDK8 | MED17 | 0.9 | Affinity Capture-MS\|Affinity Capture-Western\|MI:0004(affinity chromatography technology)\|MI:0676(tandem affinity purification)\|MI:0007(anti tag coimmunoprecipitation)\|MI:0096(pull down)\|MI:0006(anti bait coimmunoprecipitation)\|bioid |
| CDK8 | MED31 | 0.9 | Affinity Capture-MS\|MI:0004(affinity chromatography technology)\|MI:0676(tandem affinity purification)\|MI:0007(anti tag coimmunoprecipitation)\|MI:0096(pull down)\|bioid\|MI:0401(biochemical) |
| CDK8 | MED7 | 0.96 | Affinity Capture-Western\|MI:0004(affinity chromatography technology)\|MI:0676(tandem affinity purification)\|MI:0096(pull down)\|MI:0007(anti tag coimmunoprecipitation)\|bioid |
| CDK8 | MED20 | 0.9 | Affinity Capture-Western\|MI:0004(affinity chromatography technology)\|MI:0676(tandem affinity purification)\|MI:0007(anti tag coimmunoprecipitation)\|MI:0096(pull down)\|bioid |
| CDK8 | SMAD1 | 0.73 | MI:0007(anti tag coimmunoprecipitation)\|MI:0004(affinity chromatography technology)\|MI:0415(enzymatic study) |
| CDK8 | KAT2A | 0.91 | MI:0006(anti bait coimmunoprecipitation)\|MI:0226(ion exchange chromatography)\|bioid |
| CDK8 | MED15 | 0.9 | MI:0006(anti bait coimmunoprecipitation)\|MI:0004(affinity chromatography technology)\|MI:0226(ion exchange chromatography)\|MI:0676(tandem affinity purification)\|MI:0007(anti tag coimmunoprecipitation)\|MI:0096(pull down)\|bioid\|MI:0401(biochemical) |
| CDK8 | MED22 | 0.89 | MI:0004(affinity chromatography technology)\|MI:0676(tandem affinity purification)\|MI:0096(pull down)\|MI:0007(anti tag coimmunoprecipitation)\|bioid |
| CDK8 | CTDP1 | 0.83 | MI:0415(enzymatic study)\|MI:0004(affinity chromatography technology)\|MI:0007(anti tag coimmunoprecipitation) |
| CDK8 | CDK8 | 0.84 | MI:0415(enzymatic study)\|MI:0004(affinity chromatography technology)\|bioid |
| CDK8 | MED13L | 0.89 | MI:0676(tandem affinity purification)\|MI:0004(affinity chromatography technology)\|MI:0007(anti tag coimmunoprecipitation)\|MI:0096(pull down)\|bioid |
| CDK8 | HNRNPF | 0.72 | MI:0676(tandem affinity purification)\|MI:0004(affinity chromatography technology) |
| CDK8 | HERC2 | 0.72 | MI:0676(tandem affinity purification)\|MI:0004(affinity chromatography technology) |
| CDK8 | MED18 | 0.89 | MI:0676(tandem affinity purification)\|MI:0004(affinity chromatography technology)\|MI:0007(anti tag coimmunoprecipitation)\|MI:0096(pull down)\|bioid |
| CDK8 | MED4 | 0.89 | MI:0676(tandem affinity purification)\|MI:0004(affinity chromatography technology)\|MI:0007(anti tag coimmunoprecipitation)\|MI:0096(pull down)\|bioid |
| CDK8 | MED11 | 0.89 | MI:0676(tandem affinity purification)\|MI:0004(affinity chromatography technology)\|MI:0007(anti tag coimmunoprecipitation)\|MI:0096(pull down)\|bioid |
| CDK8 | MED27 | 0.89 | MI:0676(tandem affinity purification)\|MI:0004(affinity chromatography technology)\|MI:0007(anti tag coimmunoprecipitation)\|MI:0096(pull down)\|bioid |
| CDK8 | CDK9 | 0.78 | MI:0096(pull down)\|MI:0006(anti bait coimmunoprecipitation) |
| CDK8 | HSP90AA5P | 0.83 | MI:0004(affinity chromatography technology)\|MI:0007(anti tag coimmunoprecipitation) |
| CDK8 | MED12L | 0.88 | MI:0004(affinity chromatography technology)\|MI:0096(pull down)\|MI:0007(anti tag coimmunoprecipitation)\|bioid |
| CDK8 | PPP1R12A | 0.83 | MI:0004(affinity chromatography technology)\|MI:0007(anti tag coimmunoprecipitation) |
| CDK8 | CDK16 | 0.83 | MI:0004(affinity chromatography technology)\|MI:0007(anti tag coimmunoprecipitation) |
| CDK8 | CELF1 | 0.72 | MI:0004(affinity chromatography technology)\|MI:0007(anti tag coimmunoprecipitation) |
| CDK8 | SRGAP1 | 0.72 | MI:0004(affinity chromatography technology)\|MI:0007(anti tag coimmunoprecipitation) |
| CDK8 | MTBP | 0.87 | MI:0004(affinity chromatography technology)\|MI:0007(anti tag coimmunoprecipitation)\|bioid |
| CDK8 | GEMIN5 | 0.72 | MI:0004(affinity chromatography technology)\|MI:0007(anti tag coimmunoprecipitation) |
| CDK8 | WWP1 | 0.72 | MI:0004(affinity chromatography technology)\|MI:0007(anti tag coimmunoprecipitation) |
| CDK8 | BUD13 | 0.72 | MI:0004(affinity chromatography technology)\|MI:0007(anti tag coimmunoprecipitation) |
| CDK8 | DENND4C | 0.72 | MI:0004(affinity chromatography technology)\|MI:0007(anti tag coimmunoprecipitation) |
| CDK8 | RBM4 | 0.72 | MI:0004(affinity chromatography technology)\|MI:0007(anti tag coimmunoprecipitation) |
| CDK8 | POLR2C | 0.82 | MI:0004(affinity chromatography technology)\|MI:0007(anti tag coimmunoprecipitation) |
| CDK8 | UBL4A | 0.72 | MI:0004(affinity chromatography technology)\|MI:0007(anti tag coimmunoprecipitation) |
| CDK8 | ZNF131 | 0.86 | MI:0004(affinity chromatography technology)\|MI:0007(anti tag coimmunoprecipitation)\|bioid |
| CDK8 | BAG6 | 0.72 | MI:0004(affinity chromatography technology)\|MI:0007(anti tag coimmunoprecipitation) |
| CDK8 | TAF1 | 0.72 | MI:0004(affinity chromatography technology)\|MI:0007(anti tag coimmunoprecipitation) |
| CDK8 | TBX3 | 0.72 | MI:0004(affinity chromatography technology)\|MI:0007(anti tag coimmunoprecipitation) |
| CDK8 | GCFC2 | 0.72 | MI:0004(affinity chromatography technology)\|MI:0007(anti tag coimmunoprecipitation) |
| CDK8 | PCF11 | 0.72 | MI:0004(affinity chromatography technology)\|MI:0007(anti tag coimmunoprecipitation) |
| CDK8 | GET4 | 0.72 | MI:0004(affinity chromatography technology)\|MI:0007(anti tag coimmunoprecipitation) |
| CDK8 | SUPT7L | 0.72 | MI:0004(affinity chromatography technology)\|MI:0007(anti tag coimmunoprecipitation) |
| CDK8 | POLR1D | 0.72 | MI:0004(affinity chromatography technology)\|MI:0007(anti tag coimmunoprecipitation) |
| CDK8 | RBM45 | 0.72 | MI:0004(affinity chromatography technology)\|MI:0007(anti tag coimmunoprecipitation) |
| CDK8 | TP53BP1 | 0.73 | bioid |
| CDK8 | CDC37 | 0.84 | MI:0096(pull down)\|MI:0004(affinity chromatography technology)\|bioid |
| CDK8 | CCNT1 | 0.7 | MI:0006(anti bait coimmunoprecipitation) |
| CDK8 | KPNA1 | 0.7 | bioid |
| CDK9 | PRKDC | 0.74 | MI:0004(affinity chromatography technology) |
| CDX1 | CLK2 | 0.82 | MI:0004(affinity chromatography technology)\|MI:0007(anti tag coimmunoprecipitation) |
| CEACAM21 | MET | 0.83 | MI:0004(affinity chromatography technology)\|MI:0007(anti tag coimmunoprecipitation) |
| CENPA | PRKDC | 0.72 | MI:0004(affinity chromatography technology)\|MI:0676(tandem affinity purification) |
| CERK | NOTCH2NLA | 0.73 | MI:0018(two hybrid) |
| CERK | U2AF2 | 0.72 | MI:0004(affinity chromatography technology)\|MI:0007(anti tag coimmunoprecipitation) |
| CERK | CELF1 | 0.72 | MI:0004(affinity chromatography technology)\|MI:0007(anti tag coimmunoprecipitation) |
| CERK | TBC1D22A | 0.72 | MI:0004(affinity chromatography technology)\|MI:0007(anti tag coimmunoprecipitation) |
| CERK | CCDC174 | 0.72 | MI:0004(affinity chromatography technology)\|MI:0007(anti tag coimmunoprecipitation) |
| CERK | APOL2 | 0.72 | MI:0004(affinity chromatography technology)\|MI:0007(anti tag coimmunoprecipitation) |
| CERK | WDR43 | 0.72 | MI:0004(affinity chromatography technology)\|MI:0007(anti tag coimmunoprecipitation) |
| CERK | USP1 | 0.72 | MI:0004(affinity chromatography technology)\|MI:0007(anti tag coimmunoprecipitation) |
| CERK | BAG6 | 0.72 | MI:0004(affinity chromatography technology)\|MI:0007(anti tag coimmunoprecipitation) |
| CERK | TAF13 | 0.72 | MI:0004(affinity chromatography technology)\|MI:0007(anti tag coimmunoprecipitation) |
| CERK | UTP4 | 0.72 | MI:0004(affinity chromatography technology)\|MI:0007(anti tag coimmunoprecipitation) |
| CERK | PRRC2B | 0.72 | MI:0004(affinity chromatography technology)\|MI:0007(anti tag coimmunoprecipitation) |
| CERK | ILKAP | 0.72 | MI:0004(affinity chromatography technology)\|MI:0007(anti tag coimmunoprecipitation) |
| CERK | HLTF | 0.72 | MI:0004(affinity chromatography technology)\|MI:0007(anti tag coimmunoprecipitation) |
| CERK | SPG7 | 0.72 | MI:0004(affinity chromatography technology)\|MI:0007(anti tag coimmunoprecipitation) |
| CERK | DDX23 | 0.72 | MI:0004(affinity chromatography technology)\|MI:0007(anti tag coimmunoprecipitation) |
| CERK | PRPF4 | 0.72 | MI:0004(affinity chromatography technology)\|MI:0007(anti tag coimmunoprecipitation) |
| CERK | UBL4A | 0.72 | MI:0004(affinity chromatography technology)\|MI:0007(anti tag coimmunoprecipitation) |
| CERK | GEMIN2 | 0.72 | MI:0004(affinity chromatography technology)\|MI:0007(anti tag coimmunoprecipitation) |
| CERK | IK | 0.72 | MI:0004(affinity chromatography technology)\|MI:0007(anti tag coimmunoprecipitation) |
| CERK | PRMT1 | 0.72 | MI:0004(affinity chromatography technology)\|MI:0007(anti tag coimmunoprecipitation) |
| CERK | NR3C1 | 0.72 | MI:0004(affinity chromatography technology)\|MI:0007(anti tag coimmunoprecipitation) |
| CERK | ALDH18A1 | 0.72 | MI:0004(affinity chromatography technology)\|MI:0007(anti tag coimmunoprecipitation) |
| CERK | RAD18 | 0.72 | MI:0004(affinity chromatography technology)\|MI:0007(anti tag coimmunoprecipitation) |
| CERK | NCBP1 | 0.72 | MI:0004(affinity chromatography technology)\|MI:0007(anti tag coimmunoprecipitation) |
| CERK | BUB1B | 0.72 | MI:0004(affinity chromatography technology)\|MI:0007(anti tag coimmunoprecipitation) |
| CERK | EIF4ENIF1 | 0.72 | MI:0004(affinity chromatography technology)\|MI:0007(anti tag coimmunoprecipitation) |
| CERK | RBM26 | 0.72 | MI:0004(affinity chromatography technology)\|MI:0007(anti tag coimmunoprecipitation) |
| CERKL | MICAL3 | 0.82 | MI:0004(affinity chromatography technology)\|MI:0007(anti tag coimmunoprecipitation) |
| CERKL | PPM1G | 0.82 | MI:0004(affinity chromatography technology)\|MI:0007(anti tag coimmunoprecipitation) |
| CERKL | PPM1B | 0.82 | MI:0004(affinity chromatography technology)\|MI:0007(anti tag coimmunoprecipitation) |
| CERKL | PPM1A | 0.82 | MI:0004(affinity chromatography technology)\|MI:0007(anti tag coimmunoprecipitation) |
| CERKL | EIF3G | 0.82 | MI:0004(affinity chromatography technology)\|MI:0007(anti tag coimmunoprecipitation) |
| CERKL | EIF3I | 0.82 | MI:0004(affinity chromatography technology)\|MI:0007(anti tag coimmunoprecipitation) |
| CERKL | SIRT1 | 0.82 | MI:0004(affinity chromatography technology)\|MI:0007(anti tag coimmunoprecipitation) |
| CERKL | ANKHD1-EIF4EBP3 | 0.82 | MI:0004(affinity chromatography technology)\|MI:0007(anti tag coimmunoprecipitation) |
| CERKL | ERBIN | 0.72 | MI:0004(affinity chromatography technology)\|MI:0007(anti tag coimmunoprecipitation) |
| CFTR | PRKDC | 0.85 | MI:0006(anti bait coimmunoprecipitation)\|MI:0004(affinity chromatography technology)\|MI:0096(pull down) |
| CHD1L | PRKDC | 0.82 | MI:0004(affinity chromatography technology)\|MI:0007(anti tag coimmunoprecipitation) |
| CIB1 | PTK2 | 0.73 | MI:0493(in vivo)\|Affinity Capture-Western\|MI:0004(affinity chromatography technology) |
| CLK2 | PTPN1 | 0.82 | MI:0492(in vitro)\|MI:0493(in vivo)\|MI:0424(protein kinase assay)\|MI:0415(enzymatic study) |
| CLK2 | CLK2 | 0.85 | MI:0492(in vitro)\|MI:0004(affinity chromatography technology)\|MI:0018(two hybrid)\|MI:0415(enzymatic study) |
| CLK2 | CLK3 | 0.87 | MI:0018(two hybrid)\|MI:0004(affinity chromatography technology)\|MI:0007(anti tag coimmunoprecipitation) |
| CLK2 | SNRNP70 | 0.87 | MI:0007(anti tag coimmunoprecipitation)\|MI:0018(two hybrid)\|MI:0004(affinity chromatography technology) |
| CLK2 | FANCM | 0.73 | MI:0004(affinity chromatography technology) |
| CLK2 | SNIP1 | 0.88 | MI:0018(two hybrid)\|MI:0004(affinity chromatography technology)\|MI:0007(anti tag coimmunoprecipitation)\|MI:0096(pull down) |
| CLK2 | PNN | 0.87 | MI:0676(tandem affinity purification)\|MI:0004(affinity chromatography technology)\|MI:0007(anti tag coimmunoprecipitation) |
| CLK2 | FIP1L1 | 0.72 | MI:0676(tandem affinity purification)\|MI:0004(affinity chromatography technology) |
| CLK2 | PKP2 | 0.72 | MI:0676(tandem affinity purification)\|MI:0004(affinity chromatography technology) |
| CLK2 | CPSF4 | 0.72 | MI:0676(tandem affinity purification)\|MI:0004(affinity chromatography technology) |
| CLK2 | WDR33 | 0.87 | MI:0676(tandem affinity purification)\|MI:0004(affinity chromatography technology)\|MI:0007(anti tag coimmunoprecipitation) |
| CLK2 | SETD2 | 0.87 | MI:0676(tandem affinity purification)\|MI:0004(affinity chromatography technology)\|MI:0007(anti tag coimmunoprecipitation) |
| CLK2 | PABPC1 | 0.72 | MI:0676(tandem affinity purification)\|MI:0004(affinity chromatography technology) |
| CLK2 | PPHLN1 | 0.87 | MI:0676(tandem affinity purification)\|MI:0004(affinity chromatography technology)\|MI:0007(anti tag coimmunoprecipitation) |
| CLK2 | CPSF2 | 0.72 | MI:0676(tandem affinity purification)\|MI:0004(affinity chromatography technology) |
| CLK2 | TNPO3 | 0.84 | MI:0676(tandem affinity purification)\|MI:0004(affinity chromatography technology)\|MI:0096(pull down) |
| CLK2 | RBM7 | 0.72 | MI:0676(tandem affinity purification)\|MI:0004(affinity chromatography technology) |
| CLK2 | LUC7L | 0.89 | MI:0676(tandem affinity purification)\|MI:0004(affinity chromatography technology)\|MI:0007(anti tag coimmunoprecipitation)\|MI:0018(two hybrid) |
| CLK2 | ZNF30 | 0.72 | MI:0676(tandem affinity purification)\|MI:0004(affinity chromatography technology) |
| CLK2 | PSME3 | 0.84 | MI:0676(tandem affinity purification)\|MI:0004(affinity chromatography technology)\|MI:0096(pull down) |
| CLK2 | MTREX | 0.72 | MI:0676(tandem affinity purification)\|MI:0004(affinity chromatography technology) |
| CLK2 | RBBP6 | 0.87 | MI:0676(tandem affinity purification)\|MI:0004(affinity chromatography technology)\|MI:0007(anti tag coimmunoprecipitation) |
| CLK2 | HUWE1 | 0.72 | MI:0676(tandem affinity purification)\|MI:0004(affinity chromatography technology) |
| CLK2 | ZCCHC8 | 0.87 | MI:0676(tandem affinity purification)\|MI:0004(affinity chromatography technology)\|MI:0007(anti tag coimmunoprecipitation) |
| CLK2 | BCLAF1 | 0.88 | MI:0676(tandem affinity purification)\|MI:0004(affinity chromatography technology)\|MI:0096(pull down)\|MI:0007(anti tag coimmunoprecipitation) |
| CLK2 | RBM39 | 0.87 | MI:0676(tandem affinity purification)\|MI:0004(affinity chromatography technology)\|MI:0018(two hybrid) |
| CLK2 | CPSF1 | 0.72 | MI:0676(tandem affinity purification)\|MI:0004(affinity chromatography technology) |
| CLK2 | SON | 0.84 | MI:0676(tandem affinity purification)\|MI:0004(affinity chromatography technology)\|MI:0096(pull down) |
| CLK2 | HNRNPM | 0.72 | MI:0676(tandem affinity purification)\|MI:0004(affinity chromatography technology) |
| CLK2 | AKT1 | 0.7 | MI:0415(enzymatic study)\|MI:0004(affinity chromatography technology)\|MI:0096(pull down) |
| CLK2 | ACIN1 | 0.85 | MI:0004(affinity chromatography technology)\|MI:0096(pull down)\|MI:0007(anti tag coimmunoprecipitation) |
| CLK2 | THRAP3 | 0.85 | MI:0004(affinity chromatography technology)\|MI:0096(pull down)\|MI:0007(anti tag coimmunoprecipitation) |
| CLK2 | RNPS1 | 0.87 | MI:0018(two hybrid)\|MI:0004(affinity chromatography technology)\|MI:0007(anti tag coimmunoprecipitation) |
| CLK2 | SRRM1 | 0.87 | MI:0018(two hybrid)\|MI:0004(affinity chromatography technology)\|MI:0007(anti tag coimmunoprecipitation) |
| CLK2 | ECE1 | 0.73 | MI:0018(two hybrid) |
| CLK2 | BRCA1 | 0.72 | MI:0004(affinity chromatography technology)\|MI:0018(two hybrid) |
| CLK2 | LUZP4 | 0.73 | MI:0018(two hybrid) |
| CLK2 | SRPK2 | 0.73 | MI:0018(two hybrid) |
| CLK2 | TRIM27 | 0.78 | MI:0018(two hybrid)\|MI:0096(pull down) |
| CLK2 | PRPF38A | 0.73 | MI:0018(two hybrid) |
| CLK2 | CPSF7 | 0.73 | MI:0018(two hybrid) |
| CLK2 | LNX1 | 0.74 | MI:0018(two hybrid) |
| CLK2 | UBE2I | 0.73 | MI:0018(two hybrid) |
| CLK2 | YTHDC1 | 0.87 | MI:0018(two hybrid)\|MI:0004(affinity chromatography technology)\|MI:0007(anti tag coimmunoprecipitation) |
| CLK2 | ZNF398 | 0.73 | MI:0018(two hybrid) |
| CLK2 | ZRSR2 | 0.74 | MI:0018(two hybrid) |
| CLK2 | RSRP1 | 0.89 | MI:0018(two hybrid)\|MI:0004(affinity chromatography technology)\|MI:0090(protein complementation assay)\|MI:0007(anti tag coimmunoprecipitation) |
| CLK2 | KLHL2 | 0.73 | MI:0018(two hybrid) |
| CLK2 | SRRT | 0.84 | MI:0004(affinity chromatography technology)\|MI:0007(anti tag coimmunoprecipitation)\|MI:0096(pull down) |
| CLK2 | ZC3H14 | 0.82 | MI:0004(affinity chromatography technology)\|MI:0007(anti tag coimmunoprecipitation) |
| CLK2 | CLASRP | 0.87 | MI:0004(affinity chromatography technology)\|MI:0018(two hybrid)\|MI:0007(anti tag coimmunoprecipitation) |
| CLK2 | ZNF638 | 0.82 | MI:0004(affinity chromatography technology)\|MI:0007(anti tag coimmunoprecipitation) |
| CLK2 | REPIN1 | 0.82 | MI:0004(affinity chromatography technology)\|MI:0007(anti tag coimmunoprecipitation) |
| CLK2 | NCBP3 | 0.72 | MI:0004(affinity chromatography technology)\|MI:0007(anti tag coimmunoprecipitation) |
| CLK2 | GPALPP1 | 0.82 | MI:0004(affinity chromatography technology)\|MI:0007(anti tag coimmunoprecipitation) |
| CLK2 | NCOA5 | 0.82 | MI:0004(affinity chromatography technology)\|MI:0007(anti tag coimmunoprecipitation) |
| CLK2 | CLK1 | 0.87 | MI:0004(affinity chromatography technology)\|MI:0018(two hybrid)\|MI:0007(anti tag coimmunoprecipitation) |
| CLK2 | SRSF12 | 0.82 | MI:0004(affinity chromatography technology)\|MI:0007(anti tag coimmunoprecipitation) |
| CLK2 | NKTR | 0.82 | MI:0004(affinity chromatography technology)\|MI:0007(anti tag coimmunoprecipitation) |
| CLK2 | CASC3 | 0.82 | MI:0004(affinity chromatography technology)\|MI:0007(anti tag coimmunoprecipitation) |
| CLK2 | PRPF4B | 0.82 | MI:0004(affinity chromatography technology)\|MI:0007(anti tag coimmunoprecipitation) |
| CLK2 | RBMXL1 | 0.73 | MI:0004(affinity chromatography technology) |
| CLK2 | SRSF8 | 0.87 | MI:0004(affinity chromatography technology)\|MI:0007(anti tag coimmunoprecipitation)\|MI:0018(two hybrid) |
| CLK2 | SRSF10 | 0.82 | MI:0004(affinity chromatography technology)\|MI:0007(anti tag coimmunoprecipitation) |
| CLK2 | GPATCH8 | 0.82 | MI:0004(affinity chromatography technology)\|MI:0007(anti tag coimmunoprecipitation) |
| CLK2 | PRPF6 | 0.78 | MI:0096(pull down)\|MI:0018(two hybrid) |
| CLK2 | SRRM2 | 0.78 | MI:0096(pull down)\|MI:0004(affinity chromatography technology) |
| CLK2 | TRA2B | 0.7 | MI:0004(affinity chromatography technology) |
| CLN3 | PRKDC | 0.82 | MI:0007(anti tag coimmunoprecipitation)\|MI:0004(affinity chromatography technology) |
| CLTC | HGS | 0.96 | MI:0492(in vitro)\|MI:0018(two hybrid)\|Reconstituted Complex\|MI:0004(affinity chromatography technology)\|MI:0096(pull down) |
| COPS5 | PRKDC | 0.72 | MI:0004(affinity chromatography technology)\|MI:0676(tandem affinity purification) |
| CREBBP | CDK8 | 0.77 | Co-fractionation\|Affinity Capture-Western\|MI:0004(affinity chromatography technology)\|MI:0401(biochemical) |
| CRK | PTK2 | 0.9 | MI:0492(in vitro)\|MI:0493(in vivo)\|Affinity Capture-Western\|Reconstituted Complex\|MI:0004(affinity chromatography technology)\|MI:0096(pull down)\|MI:0006(anti bait coimmunoprecipitation)\|MI:0018(two hybrid) |
| CSNK1A1 | PRKDC | 0.73 | MI:0004(affinity chromatography technology) |
| CSTF2 | HGS | 0.73 | MI:0018(two hybrid) |
| CTNNB1 | MET | 0.83 | MI:0493(in vivo)\|Affinity Capture-Western\|MI:0004(affinity chromatography technology) |
| CTNNB1 | PRPF6 | 0.73 | MI:0004(affinity chromatography technology) |
| CTSB | PRKDC | 0.72 | MI:0004(affinity chromatography technology)\|bioid |
| CTSS | PRKDC | 0.72 | MI:0004(affinity chromatography technology)\|bioid |
| CTTNBP2NL | STK24 | 0.9 | Affinity Capture-MS\|Affinity Capture-Western\|MI:0007(anti tag coimmunoprecipitation)\|MI:0004(affinity chromatography technology)\|MI:0676(tandem affinity purification)\|MI:0096(pull down) |
| CUL3 | PRKDC | 0.86 | MI:0004(affinity chromatography technology)\|MI:0676(tandem affinity purification)\|MI:0030(cross-linking study) |
| CUL5 | PRKDC | 0.72 | MI:0004(affinity chromatography technology)\|MI:0676(tandem affinity purification) |
| CUL7 | PRPF6 | 0.73 | MI:0004(affinity chromatography technology) |
| CXCR4 | PTK2 | 0.74 | MI:0492(in vitro)\|MI:0493(in vivo)\|Affinity Capture-Western\|MI:0004(affinity chromatography technology) |
| DAPK3 | PRKCZ | 0.75 | MI:0492(in vitro)\|Reconstituted Complex\|MI:0096(pull down) |
| DCLRE1B | PRKDC | 0.72 | MI:0004(affinity chromatography technology)\|MI:0007(anti tag coimmunoprecipitation) |
| DCLRE1C | PRKDC | 0.9 | MI:0492(in vitro)\|MI:0493(in vivo)\|Reconstituted Complex\|Biochemical Activity\|Affinity Capture-Western\|MI:0415(enzymatic study)\|MI:0004(affinity chromatography technology)\|MI:0096(pull down)\|MI:0007(anti tag coimmunoprecipitation)\|MI:0006(anti bait coimmunoprecipitation) |
| DDA1 | PRKDC | 0.72 | MI:0007(anti tag coimmunoprecipitation)\|MI:0004(affinity chromatography technology) |
| DDX23 | PRPF6 | 0.89 | MI:0004(affinity chromatography technology)\|MI:0007(anti tag coimmunoprecipitation)\|bioid\|MI:0401(biochemical) |
| DEPDC1B | PRPF6 | 0.72 | MI:0007(anti tag coimmunoprecipitation)\|MI:0004(affinity chromatography technology) |
| DHX9 | PRKDC | 0.73 | MI:0415(enzymatic study)\|MI:0004(affinity chromatography technology)\|MI:0401(biochemical) |
| DLAT | PDK2 | 0.86 | MI:0492(in vitro)\|Reconstituted Complex\|MI:0096(pull down) |
| DLGAP1 | MAGI2 | 0.7 | MI:0018(two hybrid) |
| DLL1 | MAGI2 | 0.72 | MI:0492(in vitro)\|MI:0018(two hybrid) |
| DNAJC5 | HGS | 0.87 | MI:0012(bioluminescence resonance energy transfer)\|MI:0055(fluorescent resonance energy transfer)\|bioid |
| DNAJC7 | PRKDC | 0.72 | MI:0004(affinity chromatography technology)\|MI:0007(anti tag coimmunoprecipitation) |
| DNM2 | PTK2 | 0.75 | MI:0493(in vivo)\|MI:0004(affinity chromatography technology) |
| DPP4 | PRKDC | 0.72 | MI:0004(affinity chromatography technology)\|bioid |
| EAPP | PRPF6 | 0.73 | MI:0004(affinity chromatography technology) |
| EBP | PTK2 | 0.72 | MI:0030(cross-linking study)\|bioid |
| ECT2 | PRKCZ | 0.72 | Affinity Capture-Western\|MI:0004(affinity chromatography technology) |
| EGFR | MET | 0.9 | MI:0492(in vitro)\|MI:0493(in vivo)\|MI:0006(anti bait coimmunoprecipitation)\|MI:0676(tandem affinity purification)\|MI:0004(affinity chromatography technology)\|MI:0424(protein kinase assay) |
| EGFR | HGS | 0.89 | MI:0492(in vitro)\|Affinity Capture-Western\|MI:0004(affinity chromatography technology)\|MI:0096(pull down)\|MI:0006(anti bait coimmunoprecipitation) |
| EGFR | PRKDC | 0.73 | MI:0004(affinity chromatography technology) |
| EGFR | PRKCZ | 0.87 | MI:0018(two hybrid)\|MI:0112(ubiquitin reconstruction)\|MI:0090(protein complementation assay) |
| EGLN3 | SIK3 | 0.72 | MI:0004(affinity chromatography technology)\|MI:0007(anti tag coimmunoprecipitation) |
| EHD1 | PRKDC | 0.72 | MI:0004(affinity chromatography technology)\|MI:0401(biochemical) |
| ELAVL1 | PTK2 | 0.7 | MI:0004(affinity chromatography technology) |
| ELAVL2 | CLK2 | 0.82 | MI:0004(affinity chromatography technology)\|MI:0007(anti tag coimmunoprecipitation) |
| ELK1 | PRKDC | 0.72 | MI:0007(anti tag coimmunoprecipitation)\|MI:0004(affinity chromatography technology) |
| ENO3 | KALRN | 0.72 | MI:0399(two hybrid fragment pooling approach)\|MI:0018(two hybrid) |
| EP300 | PRKDC | 0.85 | MI:0006(anti bait coimmunoprecipitation)\|MI:0004(affinity chromatography technology)\|MI:0096(pull down) |
| EPB41L2 | CLK2 | 0.82 | MI:0004(affinity chromatography technology)\|MI:0007(anti tag coimmunoprecipitation) |
| EPHA1 | PRKDC | 0.82 | MI:0004(affinity chromatography technology)\|MI:0007(anti tag coimmunoprecipitation) |
| EPS15 | HGS | 0.9 | MI:0492(in vitro)\|MI:0493(in vivo)\|Reconstituted Complex\|MI:0018(two hybrid)\|Co-fractionation\|MI:0096(pull down)\|MI:0004(affinity chromatography technology)\|MI:0401(biochemical) |
| ERBB2 | MET | 0.73 | MI:0090(protein complementation assay)\|MI:0004(affinity chromatography technology)\|MI:0428(imaging technique) |
| ESR1 | CDK8 | 0.75 | Reconstituted Complex\|MI:0096(pull down) |
| ESR1 | PRKDC | 0.74 | MI:0004(affinity chromatography technology) |
| ESR1 | MYLK2 | 0.85 | MI:0096(pull down)\|MI:0004(affinity chromatography technology)\|MI:0676(tandem affinity purification) |
| ESR2 | CDK8 | 0.75 | Reconstituted Complex\|MI:0096(pull down) |
| ESR2 | MYLK2 | 0.84 | MI:0004(affinity chromatography technology)\|MI:0029(cosedimentation through density gradient)\|MI:0676(tandem affinity purification) |
| F11R | PRKCZ | 0.8 | MI:0059(gst pull down)\|MI:0096(pull down)\|MI:0428(imaging technique) |
| FADD | PRKCZ | 0.83 | MI:0493(in vivo)\|Affinity Capture-Western\|MI:0004(affinity chromatography technology) |
| FAM118A | MOB3C | 0.73 | MI:0018(two hybrid) |
| FBXO6 | MET | 0.73 | MI:0004(affinity chromatography technology) |
| FECH | PDK2 | 0.82 | MI:0004(affinity chromatography technology)\|MI:0007(anti tag coimmunoprecipitation) |
| FEZ1 | PRKCZ | 0.83 | MI:0493(in vivo)\|MI:0018(two hybrid)\|Affinity Capture-Western\|MI:0004(affinity chromatography technology) |
| FEZ2 | PRKCZ | 0.73 | MI:0493(in vivo)\|Affinity Capture-Western\|MI:0004(affinity chromatography technology) |
| FLII | PRKDC | 0.72 | MI:0004(affinity chromatography technology)\|MI:0401(biochemical) |
| FOXD4 | PDK2 | 0.82 | MI:0004(affinity chromatography technology)\|MI:0007(anti tag coimmunoprecipitation) |
| FYN | PRPF6 | 0.72 | MI:0007(anti tag coimmunoprecipitation)\|MI:0004(affinity chromatography technology) |
| FZR1 | MET | 0.75 | MI:0055(fluorescent resonance energy transfer) |
| GAB2 | MET | 0.72 | Affinity Capture-Western\|MI:0004(affinity chromatography technology) |
| GABARAP | PRKCZ | 0.84 | MI:0007(anti tag coimmunoprecipitation)\|MI:0004(affinity chromatography technology)\|MI:0096(pull down) |
| GAK | TNK2 | 0.72 | MI:0004(affinity chromatography technology)\|MI:0007(anti tag coimmunoprecipitation) |
| GALT | TNK2 | 0.73 | MI:0018(two hybrid) |
| GCFC2 | PRPF6 | 0.83 | MI:0004(affinity chromatography technology)\|MI:0007(anti tag coimmunoprecipitation) |
| GIT1 | PTK2 | 0.9 | MI:0492(in vitro)\|MI:0493(in vivo)\|Affinity Capture-Western\|Reconstituted Complex\|MI:0004(affinity chromatography technology)\|MI:0096(pull down) |
| GJA1 | HGS | 0.75 | MI:0428(imaging technique)\|bioid |
| GLIS2 | MET | 0.75 | MI:0055(fluorescent resonance energy transfer) |
| GLMN | MET | 0.79 | MI:0493(in vivo)\|MI:0018(two hybrid)\|Reconstituted Complex\|Affinity Capture-Western\|MI:0004(affinity chromatography technology)\|MI:0096(pull down) |
| GMNN | CERK | 0.82 | MI:0004(affinity chromatography technology)\|MI:0007(anti tag coimmunoprecipitation) |
| GOLT1B | HGS | 0.72 | MI:0004(affinity chromatography technology)\|MI:0007(anti tag coimmunoprecipitation) |
| GPR156 | PRPF6 | 0.82 | MI:0004(affinity chromatography technology)\|MI:0007(anti tag coimmunoprecipitation) |
| GRB14 | PRKCZ | 0.73 | MI:0493(in vivo)\|Affinity Capture-Western\|MI:0004(affinity chromatography technology) |
| GRB14 | MET | 0.75 | MI:0053(fluorescence polarization spectroscopy)\|MI:0096(pull down) |
| GRB2 | HGS | 0.72 | MI:0007(anti tag coimmunoprecipitation)\|MI:0004(affinity chromatography technology) |
| GRB7 | PTK2 | 0.96 | MI:0492(in vitro)\|MI:0493(in vivo)\|Reconstituted Complex\|Affinity Capture-Western\|MI:0004(affinity chromatography technology)\|MI:0096(pull down)\|MI:0018(two hybrid) |
| GSK3B | PTK2 | 0.83 | MI:0493(in vivo)\|Affinity Capture-Western\|MI:0004(affinity chromatography technology) |
| GSN | PTK2 | 0.72 | Affinity Capture-Western\|MI:0004(affinity chromatography technology) |
| GSPT2 | CLK2 | 0.82 | MI:0004(affinity chromatography technology)\|MI:0007(anti tag coimmunoprecipitation) |
| GSTT1 | MYLK2 | 0.82 | MI:0004(affinity chromatography technology)\|MI:0007(anti tag coimmunoprecipitation) |
| GTF2A1 | MYLK2 | 0.72 | Affinity Capture-MS\|MI:0004(affinity chromatography technology) |
| GTF2F1 | CDK8 | 0.82 | MI:0004(affinity chromatography technology)\|bioid |
| GTSE1 | TNK2 | 0.72 | MI:0004(affinity chromatography technology)\|MI:0007(anti tag coimmunoprecipitation) |
| H2AFX | PRKDC | 0.86 | MI:0006(anti bait coimmunoprecipitation)\|MI:0415(enzymatic study)\|MI:0004(affinity chromatography technology)\|MI:0096(pull down) |
| HAVCR2 | CERK | 0.82 | MI:0004(affinity chromatography technology)\|MI:0007(anti tag coimmunoprecipitation) |
| HCK | PTK2 | 0.72 | MI:0004(affinity chromatography technology)\|MI:0018(two hybrid) |
| HDAC11 | PRKDC | 0.72 | MI:0007(anti tag coimmunoprecipitation)\|MI:0004(affinity chromatography technology) |
| HDAC11 | PRPF6 | 0.72 | MI:0007(anti tag coimmunoprecipitation)\|MI:0004(affinity chromatography technology) |
| HDGF | PRKDC | 0.75 | MI:0004(affinity chromatography technology)\|MI:0096(pull down)\|MI:0676(tandem affinity purification) |
| HDLBP | PRKDC | 0.72 | Affinity Capture-Western\|MI:0004(affinity chromatography technology) |
| HEPACAM2 | CERK | 0.83 | MI:0004(affinity chromatography technology)\|MI:0007(anti tag coimmunoprecipitation) |
| HGH1 | HGS | 0.72 | MI:0004(affinity chromatography technology)\|MI:0401(biochemical) |
| HGS | SNX1 | 0.89 | MI:0493(in vivo)\|Reconstituted Complex\|MI:0096(pull down)\|MI:0004(affinity chromatography technology) |
| HGS | TSG101 | 0.97 | MI:0493(in vivo)\|Affinity Capture-Western\|MI:0007(anti tag coimmunoprecipitation)\|MI:0416(fluorescence microscopy)\|MI:0018(two hybrid)\|MI:0004(affinity chromatography technology)\|MI:0096(pull down)\|MI:0428(imaging technique)\|MI:0114(x-ray crystallography)\|MI:0065(isothermal titration calorimetry) |
| HGS | STAM | 0.97 | MI:0493(in vivo)\|Affinity Capture-Western\|Co-fractionation\|MI:0018(two hybrid)\|MI:0004(affinity chromatography technology)\|MI:0096(pull down)\|MI:0428(imaging technique)\|MI:0071(molecular sieving)\|MI:0114(x-ray crystallography)\|MI:0038(dynamic light scattering)\|MI:0028(cosedimentation in solution)\|MI:0067(light scattering)\|MI:0007(anti tag coimmunoprecipitation)\|MI:0401(biochemical) |
| HGS | NEDD4 | 0.88 | MI:0492(in vitro)\|MI:0493(in vivo)\|Biochemical Activity\|MI:0007(anti tag coimmunoprecipitation)\|MI:0415(enzymatic study)\|MI:0004(affinity chromatography technology) |
| HGS | PAK1 | 0.85 | MI:0493(in vivo)\|Reconstituted Complex\|MI:0096(pull down) |
| HGS | MAP3K7 | 0.86 | MI:0492(in vitro)\|MI:0493(in vivo)\|Reconstituted Complex\|MI:0096(pull down) |
| HGS | DLG4 | 0.86 | MI:0493(in vivo)\|MI:0018(two hybrid)\|Affinity Capture-Western\|MI:0004(affinity chromatography technology) |
| HGS | MED7 | 0.74 | MI:0018(two hybrid) |
| HGS | UBQLN1 | 0.83 | MI:0018(two hybrid)\|MI:0004(affinity chromatography technology) |
| HGS | STAM2 | 0.96 | Co-fractionation\|Affinity Capture-Western\|MI:0018(two hybrid)\|MI:0004(affinity chromatography technology)\|MI:0428(imaging technique)\|MI:0401(biochemical) |
| HGS | BEGAIN | 0.73 | MI:0018(two hybrid) |
| HGS | EHMT2 | 0.73 | MI:0018(two hybrid) |
| HGS | DAZAP2 | 0.74 | MI:0018(two hybrid) |
| HGS | UBE2I | 0.74 | MI:0018(two hybrid) |
| HGS | EXOC8 | 0.73 | MI:0018(two hybrid) |
| HGS | CCDC33 | 0.74 | MI:0018(two hybrid) |
| HGS | MIF4GD | 0.74 | MI:0018(two hybrid) |
| HGS | EXOC7 | 0.74 | MI:0018(two hybrid) |
| HGS | GKAP1 | 0.73 | MI:0018(two hybrid) |
| HGS | USHBP1 | 0.74 | MI:0018(two hybrid) |
| HGS | HAP1 | 0.9 | MI:0492(in vitro)\|MI:0493(in vivo)\|MI:0018(two hybrid)\|Reconstituted Complex\|Affinity Capture-Western\|MI:0004(affinity chromatography technology)\|MI:0096(pull down) |
| HGS | MET | 0.76 | MI:0493(in vivo)\|MI:0004(affinity chromatography technology) |
| HGS | SCAMP3 | 0.72 | Affinity Capture-Western\|MI:0004(affinity chromatography technology) |
| HGS | APP | 0.85 | Reconstituted Complex\|MI:0096(pull down) |
| HGS | APLP2 | 0.75 | Reconstituted Complex\|MI:0096(pull down) |
| HGS | ATP2A2 | 0.75 | Reconstituted Complex\|MI:0096(pull down) |
| HGS | ARL6IP1 | 0.75 | Reconstituted Complex\|MI:0096(pull down) |
| HGS | BSG | 0.75 | Reconstituted Complex\|MI:0096(pull down) |
| HGS | BRINP3 | 0.75 | Reconstituted Complex\|MI:0096(pull down) |
| HGS | AHCYL1 | 0.75 | Reconstituted Complex\|MI:0096(pull down) |
| HGS | CASK | 0.75 | Reconstituted Complex\|MI:0096(pull down) |
| HGS | TJP2 | 0.75 | Reconstituted Complex\|MI:0096(pull down) |
| HGS | HSPA8 | 0.87 | Reconstituted Complex\|MI:0096(pull down)\|MI:0004(affinity chromatography technology) |
| HGS | MAP3K10 | 0.75 | Reconstituted Complex\|MI:0096(pull down) |
| HGS | STXBP1 | 0.75 | Reconstituted Complex\|MI:0096(pull down) |
| HGS | SCRN1 | 0.75 | Reconstituted Complex\|MI:0096(pull down) |
| HGS | CRMP1 | 0.75 | Reconstituted Complex\|MI:0096(pull down) |
| HGS | DCTN2 | 0.75 | Reconstituted Complex\|MI:0096(pull down) |
| HGS | GFAP | 0.75 | Reconstituted Complex\|MI:0096(pull down) |
| HGS | MARK4 | 0.75 | Reconstituted Complex\|MI:0096(pull down) |
| HGS | PPP1R16A | 0.75 | Reconstituted Complex\|MI:0096(pull down) |
| HGS | RHOBTB3 | 0.75 | Reconstituted Complex\|MI:0096(pull down) |
| HGS | TUBB2A | 0.75 | Reconstituted Complex\|MI:0096(pull down) |
| HGS | ESRRG | 0.75 | Reconstituted Complex\|MI:0096(pull down) |
| HGS | ILKAP | 0.75 | Reconstituted Complex\|MI:0096(pull down) |
| HGS | PPP1R7 | 0.75 | Reconstituted Complex\|MI:0096(pull down) |
| HGS | RSU1 | 0.75 | Reconstituted Complex\|MI:0096(pull down) |
| HGS | UBA1 | 0.84 | Reconstituted Complex\|MI:0096(pull down) |
| HGS | ACLY | 0.75 | Reconstituted Complex\|MI:0096(pull down) |
| HGS | CBS | 0.75 | Reconstituted Complex\|MI:0096(pull down) |
| HGS | DECR1 | 0.75 | Reconstituted Complex\|MI:0096(pull down) |
| HGS | MAT2A | 0.75 | Reconstituted Complex\|MI:0096(pull down) |
| HGS | MTHFD1L | 0.75 | Reconstituted Complex\|MI:0096(pull down) |
| HGS | OSBPL5 | 0.75 | Reconstituted Complex\|MI:0096(pull down) |
| HGS | PLD3 | 0.75 | Reconstituted Complex\|MI:0096(pull down) |
| HGS | PFKM | 0.75 | Reconstituted Complex\|MI:0096(pull down) |
| HGS | HNRNPDL | 0.75 | Reconstituted Complex\|MI:0096(pull down) |
| HGS | RPS3A | 0.75 | Reconstituted Complex\|MI:0096(pull down) |
| HGS | SF3B3 | 0.75 | Reconstituted Complex\|MI:0096(pull down) |
| HGS | LINC00265 | 0.75 | Reconstituted Complex\|MI:0096(pull down) |
| HGS | PTCD3 | 0.75 | Reconstituted Complex\|MI:0096(pull down) |
| HGS | ZNF302 | 0.75 | Reconstituted Complex\|MI:0096(pull down) |
| HGS | MEST | 0.75 | Reconstituted Complex\|MI:0096(pull down) |
| HGS | TMCC2 | 0.75 | Reconstituted Complex\|MI:0096(pull down) |
| HGS | ATP1A1 | 0.75 | Reconstituted Complex\|MI:0096(pull down) |
| HGS | TRAP1 | 0.75 | Reconstituted Complex\|MI:0096(pull down) |
| HGS | GGA2 | 0.87 | Reconstituted Complex\|MI:0018(two hybrid)\|MI:0096(pull down) |
| HGS | TUBB | 0.75 | Reconstituted Complex\|MI:0096(pull down) |
| HGS | CEP55 | 0.74 | MI:0018(two hybrid) |
| HGS | ITCH | 0.73 | MI:0004(affinity chromatography technology) |
| HGS | LURAP1 | 0.73 | MI:0018(two hybrid) |
| HGS | TMEM189 | 0.73 | MI:0004(affinity chromatography technology)\|MI:0055(fluorescent resonance energy transfer) |
| HGS | ACTN4 | 0.88 | MI:0004(affinity chromatography technology)\|MI:0018(two hybrid)\|MI:0428(imaging technique)\|MI:0401(biochemical) |
| HGS | ARRB2 | 0.82 | MI:0007(anti tag coimmunoprecipitation)\|MI:0004(affinity chromatography technology) |
| HGS | WDR77 | 0.72 | MI:0004(affinity chromatography technology)\|MI:0401(biochemical) |
| HGS | REPIN1 | 0.72 | MI:0004(affinity chromatography technology)\|MI:0401(biochemical) |
| HGS | ABI2 | 0.73 | MI:0018(two hybrid) |
| HGS | NDC80 | 0.73 | MI:0018(two hybrid) |
| HGS | ING5 | 0.73 | MI:0018(two hybrid) |
| HGS | POGZ | 0.73 | MI:0018(two hybrid) |
| HGS | MRFAP1L1 | 0.73 | MI:0018(two hybrid) |
| HGS | INTS4 | 0.73 | MI:0018(two hybrid) |
| HGS | KRT40 | 0.73 | MI:0018(two hybrid) |
| HGS | NUP54 | 0.73 | MI:0018(two hybrid) |
| HGS | P4HA3 | 0.73 | MI:0018(two hybrid) |
| HGS | LITAF | 0.73 | MI:0018(two hybrid) |
| HGS | CCDC114 | 0.83 | MI:0018(two hybrid)\|MI:0004(affinity chromatography technology) |
| HGS | MAPK1IP1L | 0.73 | MI:0018(two hybrid) |
| HGS | CEP63 | 0.73 | MI:0018(two hybrid) |
| HGS | CDR2 | 0.73 | MI:0018(two hybrid) |
| HGS | CRX | 0.73 | MI:0018(two hybrid) |
| HGS | TEKT1 | 0.73 | MI:0018(two hybrid) |
| HGS | KRT33B | 0.73 | MI:0018(two hybrid) |
| HGS | KRT13 | 0.73 | MI:0018(two hybrid) |
| HGS | C1orf94 | 0.73 | MI:0018(two hybrid) |
| HGS | DYDC1 | 0.73 | MI:0018(two hybrid) |
| HGS | TRIM27 | 0.73 | MI:0018(two hybrid) |
| HGS | MAGED1 | 0.73 | MI:0018(two hybrid) |
| HGS | TRAF4 | 0.73 | MI:0018(two hybrid) |
| HGS | KIAA0753 | 0.73 | MI:0018(two hybrid) |
| HGS | FAM168A | 0.73 | MI:0018(two hybrid) |
| HGS | NUP62 | 0.73 | MI:0018(two hybrid) |
| HGS | TFG | 0.73 | MI:0018(two hybrid) |
| HGS | VPS37B | 0.73 | MI:0018(two hybrid) |
| HGS | TRIM17 | 0.73 | MI:0018(two hybrid) |
| HGS | TRIM69 | 0.73 | MI:0018(two hybrid) |
| HIF1AN | SIK3 | 0.72 | MI:0004(affinity chromatography technology)\|MI:0007(anti tag coimmunoprecipitation) |
| HIST1H3A | PRKDC | 0.89 | MI:0004(affinity chromatography technology)\|MI:0428(imaging technique)\|MI:0676(tandem affinity purification)\|bioid\|MI:0401(biochemical) |
| HIST3H3 | PRKCH | 0.72 | MI:0030(cross-linking study)\|bioid |
| HIST4H4 | PRPF6 | 0.73 | MI:0004(affinity chromatography technology) |
| HIST4H4 | PRKDC | 0.73 | MI:0004(affinity chromatography technology) |
| HOXB7 | PRKDC | 0.75 | MI:0096(pull down)\|MI:0006(anti bait coimmunoprecipitation)\|MI:0004(affinity chromatography technology) |
| HSCB | STK24 | 0.72 | MI:0006(anti bait coimmunoprecipitation)\|MI:0004(affinity chromatography technology) |
| HSH2D | TNK2 | 0.88 | MI:0493(in vivo)\|MI:0018(two hybrid)\|Biochemical Activity\|Affinity Capture-Western\|MI:0415(enzymatic study)\|MI:0004(affinity chromatography technology) |
| HSP90AA1 | MYLK2 | 0.87 | MI:0676(tandem affinity purification)\|MI:0004(affinity chromatography technology)\|MI:0007(anti tag coimmunoprecipitation) |
| HSP90AA1 | CLK2 | 0.78 | MI:0004(affinity chromatography technology)\|MI:0096(pull down) |
| HSP90AB1 | PRKDC | 0.72 | MI:0006(anti bait coimmunoprecipitation)\|MI:0004(affinity chromatography technology) |
| HSP90AB1 | MYLK2 | 0.82 | MI:0007(anti tag coimmunoprecipitation)\|MI:0004(affinity chromatography technology) |
| HSP90AB1 | PRKCZ | 0.82 | MI:0007(anti tag coimmunoprecipitation)\|MI:0004(affinity chromatography technology) |
| HSP90AB2P | TNK2 | 0.85 | MI:0492(in vitro)\|MI:0493(in vivo)\|MI:0004(affinity chromatography technology)\|MI:0007(anti tag coimmunoprecipitation) |
| HSPA5 | PRKDC | 0.72 | MI:0004(affinity chromatography technology)\|MI:0007(anti tag coimmunoprecipitation) |
| HSPA8 | PRKDC | 0.82 | MI:0004(affinity chromatography technology)\|MI:0401(biochemical) |
| HSPBP1 | HGS | 0.82 | MI:0004(affinity chromatography technology)\|MI:0007(anti tag coimmunoprecipitation) |
| HTT | PDK2 | 0.73 | MI:0018(two hybrid) |
| HTT | HGS | 0.72 | MI:0007(anti tag coimmunoprecipitation)\|MI:0004(affinity chromatography technology) |
| IGF1R | PTK2 | 0.72 | Affinity Capture-Western\|MI:0004(affinity chromatography technology) |
| IKBKG | PRKDC | 0.86 | MI:0676(tandem affinity purification)\|MI:0007(anti tag coimmunoprecipitation)\|MI:0004(affinity chromatography technology)\|MI:0415(enzymatic study) |
| IL20RA | CERK | 0.82 | MI:0004(affinity chromatography technology)\|MI:0007(anti tag coimmunoprecipitation) |
| IL2RB | HGS | 0.88 | Affinity Capture-Western\|Reconstituted Complex\|MI:0004(affinity chromatography technology)\|MI:0096(pull down) |
| ILF2 | PRKDC | 0.89 | MI:0492(in vitro)\|MI:0493(in vivo)\|MI:0025(copurification)\|Affinity Capture-Western\|MI:0004(affinity chromatography technology)\|MI:0401(biochemical) |
| ILK | PRKDC | 0.88 | MI:0676(tandem affinity purification)\|MI:0004(affinity chromatography technology)\|MI:0007(anti tag coimmunoprecipitation)\|MI:0096(pull down) |
| INPPL1 | MET | 0.89 | MI:0492(in vitro)\|MI:0493(in vivo)\|MI:0018(two hybrid)\|MI:0004(affinity chromatography technology)\|MI:0053(fluorescence polarization spectroscopy)\|MI:0096(pull down) |
| IQCB1 | PRKDC | 0.72 | MI:0007(anti tag coimmunoprecipitation)\|MI:0004(affinity chromatography technology) |
| IRAK1 | PRKCZ | 0.73 | MI:0493(in vivo)\|Affinity Capture-Western\|MI:0004(affinity chromatography technology) |
| ISG15 | PRKDC | 0.78 | MI:0096(pull down)\|MI:0004(affinity chromatography technology) |
| ITGB4 | PTK2 | 0.83 | MI:0492(in vitro)\|MI:0007(anti tag coimmunoprecipitation)\|MI:0006(anti bait coimmunoprecipitation) |
| ITGB5 | PTK2 | 0.95 | Reconstituted Complex\|Affinity Capture-Western\|MI:0004(affinity chromatography technology)\|MI:0096(pull down) |
| JAK1 | PRKCZ | 0.77 | MI:0492(in vitro)\|MI:0493(in vivo)\|MI:0004(affinity chromatography technology) |
| JPH4 | CLK2 | 0.72 | MI:0004(affinity chromatography technology)\|MI:0007(anti tag coimmunoprecipitation) |
| KALRN | PAM | 0.79 | MI:0018(two hybrid) |
| KALRN | DISC1 | 0.83 | MI:0018(two hybrid)\|MI:0399(two hybrid fragment pooling approach) |
| KALRN | NDEL1 | 0.83 | MI:0018(two hybrid)\|MI:0399(two hybrid fragment pooling approach) |
| KBTBD7 | BRSK2 | 0.72 | MI:0007(anti tag coimmunoprecipitation)\|MI:0004(affinity chromatography technology) |
| KCTD17 | CERK | 0.83 | MI:0004(affinity chromatography technology)\|MI:0007(anti tag coimmunoprecipitation) |
| KDELR2 | MET | 0.75 | MI:0055(fluorescent resonance energy transfer) |
| KIAA1143 | PRPF6 | 0.82 | MI:0004(affinity chromatography technology)\|MI:0007(anti tag coimmunoprecipitation) |
| KIF21B | PRPF6 | 0.72 | MI:0004(affinity chromatography technology)\|MI:0007(anti tag coimmunoprecipitation) |
| KRT15 | HGS | 0.73 | MI:0018(two hybrid) |
| KRT18 | HGS | 0.74 | MI:0018(two hybrid) |
| KRT19 | HGS | 0.83 | MI:0018(two hybrid)\|bioid |
| KRT31 | HGS | 0.83 | MI:0018(two hybrid)\|MI:0004(affinity chromatography technology) |
| KRT38 | HGS | 0.74 | MI:0018(two hybrid) |
| KRT6A | HGS | 0.73 | MI:0018(two hybrid) |
| LCK | PTK2 | 0.77 | MI:0492(in vitro)\|MI:0493(in vivo)\|MI:0018(two hybrid) |
| LCK | MET | 0.75 | MI:0053(fluorescence polarization spectroscopy)\|MI:0096(pull down) |
| LDOC1 | HGS | 0.74 | MI:0018(two hybrid) |
| LGALS9 | MET | 0.83 | MI:0004(affinity chromatography technology)\|MI:0007(anti tag coimmunoprecipitation) |
| LLGL1 | PRKCZ | 0.92 | MI:0007(anti tag coimmunoprecipitation)\|MI:0004(affinity chromatography technology)\|MI:0096(pull down) |
| LMNA | PRKDC | 0.72 | MI:0004(affinity chromatography technology)\|MI:0007(anti tag coimmunoprecipitation) |
| LMNA | PRPF6 | 0.82 | MI:0004(affinity chromatography technology)\|MI:0018(two hybrid) |
| LPAR4 | PRKDC | 0.72 | MI:0004(affinity chromatography technology)\|MI:0007(anti tag coimmunoprecipitation) |
| LPXN | PTK2 | 0.88 | MI:0493(in vivo)\|MI:0018(two hybrid)\|MI:0004(affinity chromatography technology)\|MI:0007(anti tag coimmunoprecipitation) |
| LRIG1 | MET | 0.86 | Affinity Capture-Western\|MI:0004(affinity chromatography technology)\|MI:0428(imaging technique)\|MI:0006(anti bait coimmunoprecipitation) |
| LRRFIP1 | PRKDC | 0.72 | MI:0004(affinity chromatography technology)\|MI:0401(biochemical) |
| LRRK2 | PRKDC | 0.83 | MI:0004(affinity chromatography technology)\|MI:0007(anti tag coimmunoprecipitation) |
| LRRK2 | PRPF6 | 0.82 | MI:0676(tandem affinity purification)\|MI:0007(anti tag coimmunoprecipitation) |
| LSM1 | PRPF6 | 0.7 | bioid |
| LSM2 | PRPF6 | 0.7 | bioid |
| LSM3 | PRPF6 | 0.7 | MI:0004(affinity chromatography technology) |
| LSM4 | PRPF6 | 0.82 | MI:0004(affinity chromatography technology)\|bioid |
| LSM5 | PRPF6 | 0.7 | MI:0004(affinity chromatography technology) |
| LSM6 | PRPF6 | 0.7 | MI:0004(affinity chromatography technology) |
| LSM7 | PRPF6 | 0.7 | MI:0004(affinity chromatography technology) |
| LTBR | HGS | 0.83 | MI:0004(affinity chromatography technology)\|MI:0007(anti tag coimmunoprecipitation) |
| LUC7L2 | CLK2 | 0.83 | MI:0004(affinity chromatography technology)\|MI:0007(anti tag coimmunoprecipitation) |
| LYN | MET | 0.87 | MI:0053(fluorescence polarization spectroscopy)\|MI:0096(pull down)\|bioid |
| LYST | HGS | 0.87 | MI:0492(in vitro)\|MI:0018(two hybrid)\|Reconstituted Complex\|MI:0096(pull down) |
| LZIC | CDK8 | 0.72 | MI:0004(affinity chromatography technology)\|MI:0007(anti tag coimmunoprecipitation) |
| MAGI2 | ADRB1 | 0.9 | MI:0492(in vitro)\|MI:0493(in vivo)\|MI:0018(two hybrid)\|MI:0081(peptide array)\|MI:0007(anti tag coimmunoprecipitation)\|MI:0096(pull down)\|MI:0022(colocalization by immunostaining)\|MI:0045(experimental interaction detection)\|MI:0428(imaging technique)\|MI:0004(affinity chromatography technology)\|MI:0047(far western blotting) |
| MAGI2 | PTEN | 0.88 | MI:0493(in vivo)\|MI:0007(anti tag coimmunoprecipitation)\|MI:0018(two hybrid)\|MI:0096(pull down)\|MI:0021(colocalization by fluorescent probes cloning)\|MI:0004(affinity chromatography technology) |
| MAGI2 | ATN1 | 0.78 | MI:0492(in vitro)\|MI:0018(two hybrid)\|Reconstituted Complex\|MI:0096(pull down) |
| MAGI2 | ACVR2A | 0.75 | MI:0492(in vitro)\|MI:0018(two hybrid) |
| MAGI2 | CTNNB1 | 0.82 | MI:0493(in vivo)\|MI:0004(affinity chromatography technology) |
| MAGI2 | MAGI2 | 0.82 | MI:0493(in vivo)\|MI:0018(two hybrid) |
| MAGI2 | DLGAP2 | 0.7 | MI:0018(two hybrid) |
| MAGIX | STK24 | 0.83 | MI:0004(affinity chromatography technology)\|MI:0007(anti tag coimmunoprecipitation) |
| MAP2K3 | MET | 0.75 | MI:0055(fluorescent resonance energy transfer) |
| MAP2K3 | MAP3K6 | 0.72 | MI:0007(anti tag coimmunoprecipitation)\|MI:0004(affinity chromatography technology) |
| MAP2K5 | PRKCZ | 0.88 | MI:0493(in vivo)\|Affinity Capture-Western\|Reconstituted Complex\|MI:0004(affinity chromatography technology)\|MI:0096(pull down) |
| MAP2K5 | MET | 0.75 | MI:0055(fluorescent resonance energy transfer) |
| MAP3K1 | MYLK2 | 0.72 | MI:0676(tandem affinity purification)\|MI:0007(anti tag coimmunoprecipitation) |
| MAP3K3 | PRKDC | 0.72 | MI:0676(tandem affinity purification)\|MI:0007(anti tag coimmunoprecipitation) |
| MAP3K3 | MYLK2 | 0.72 | MI:0676(tandem affinity purification)\|MI:0007(anti tag coimmunoprecipitation) |
| MAP3K6 | MAP3K5 | 0.89 | MI:0492(in vitro)\|MI:0493(in vivo)\|Affinity Capture-Western\|MI:0018(two hybrid)\|MI:0004(affinity chromatography technology)\|MI:0007(anti tag coimmunoprecipitation) |
| MAP3K6 | YWHAG | 0.87 | MI:0019(coimmunoprecipitation)\|MI:0007(anti tag coimmunoprecipitation)\|MI:0004(affinity chromatography technology) |
| MAP3K6 | PCDHA4 | 0.82 | MI:0004(affinity chromatography technology)\|MI:0007(anti tag coimmunoprecipitation) |
| MAP3K6 | FKBP5 | 0.82 | MI:0004(affinity chromatography technology)\|MI:0007(anti tag coimmunoprecipitation) |
| MAP3K6 | HSP90AA4P | 0.82 | MI:0004(affinity chromatography technology)\|MI:0007(anti tag coimmunoprecipitation) |
| MAP3K6 | HSP90AB4P | 0.82 | MI:0004(affinity chromatography technology)\|MI:0007(anti tag coimmunoprecipitation) |
| MAP3K6 | ASPM | 0.72 | MI:0004(affinity chromatography technology)\|MI:0007(anti tag coimmunoprecipitation) |
| MAP3K6 | HSP90AA5P | 0.82 | MI:0004(affinity chromatography technology)\|MI:0007(anti tag coimmunoprecipitation) |
| MAP3K6 | USP4 | 0.82 | MI:0004(affinity chromatography technology)\|MI:0007(anti tag coimmunoprecipitation) |
| MAP3K6 | LIMD1 | 0.82 | MI:0004(affinity chromatography technology)\|MI:0007(anti tag coimmunoprecipitation) |
| MAP3K6 | RNF123 | 0.82 | MI:0004(affinity chromatography technology)\|MI:0007(anti tag coimmunoprecipitation) |
| MAP3K6 | UBAC1 | 0.82 | MI:0004(affinity chromatography technology)\|MI:0007(anti tag coimmunoprecipitation) |
| MAP3K6 | CDC37 | 0.72 | MI:0004(affinity chromatography technology)\|MI:0007(anti tag coimmunoprecipitation) |
| MAP3K6 | ERGIC2 | 0.72 | MI:0004(affinity chromatography technology)\|MI:0007(anti tag coimmunoprecipitation) |
| MAPK7 | PRKCZ | 0.73 | MI:0415(enzymatic study)\|MI:0018(two hybrid)\|MI:0004(affinity chromatography technology) |
| MAPK8IP3 | PTK2 | 0.86 | MI:0492(in vitro)\|MI:0493(in vivo)\|Affinity Capture-Western\|MI:0004(affinity chromatography technology)\|MI:0096(pull down) |
| MAU2 | MOB3C | 0.73 | MI:0004(affinity chromatography technology) |
| MCC | STK24 | 0.76 | Affinity Capture-MS\|MI:0004(affinity chromatography technology)\|MI:0006(anti bait coimmunoprecipitation) |
| MDC1 | PRKDC | 0.7 | MI:0004(affinity chromatography technology)\|MI:0096(pull down)\|MI:0428(imaging technique) |
| MDK | CLK2 | 0.82 | MI:0004(affinity chromatography technology)\|MI:0007(anti tag coimmunoprecipitation) |
| MDM2 | PRPF6 | 0.73 | MI:0004(affinity chromatography technology) |
| MED10 | CDK8 | 0.97 | Affinity Capture-MS\|Affinity Capture-Western\|MI:0007(anti tag coimmunoprecipitation)\|MI:0004(affinity chromatography technology)\|MI:0676(tandem affinity purification)\|MI:0096(pull down)\|bioid\|MI:0401(biochemical) |
| MED19 | CDK8 | 0.89 | Affinity Capture-MS\|MI:0007(anti tag coimmunoprecipitation)\|MI:0004(affinity chromatography technology)\|MI:0096(pull down)\|bioid |
| MED21 | CDK8 | 0.9 | MI:0493(in vivo)\|Co-fractionation\|Affinity Capture-Western\|Affinity Capture-MS\|MI:0004(affinity chromatography technology)\|MI:0676(tandem affinity purification)\|MI:0007(anti tag coimmunoprecipitation)\|MI:0096(pull down)\|bioid\|MI:0401(biochemical) |
| MED25 | CDK8 | 0.9 | MI:0493(in vivo)\|Affinity Capture-MS\|MI:0004(affinity chromatography technology)\|MI:0007(anti tag coimmunoprecipitation)\|MI:0096(pull down)\|MI:0006(anti bait coimmunoprecipitation)\|bioid |
| MED26 | CDK8 | 0.9 | MI:0492(in vitro)\|Affinity Capture-MS\|Affinity Capture-Western\|MI:0007(anti tag coimmunoprecipitation)\|MI:0004(affinity chromatography technology)\|MI:0096(pull down)\|MI:0006(anti bait coimmunoprecipitation) |
| MED28 | CDK8 | 0.9 | Affinity Capture-MS\|MI:0004(affinity chromatography technology)\|MI:0676(tandem affinity purification)\|MI:0007(anti tag coimmunoprecipitation)\|MI:0096(pull down)\|bioid |
| MED29 | CDK8 | 0.9 | Affinity Capture-MS\|MI:0007(anti tag coimmunoprecipitation)\|MI:0004(affinity chromatography technology)\|MI:0676(tandem affinity purification)\|MI:0096(pull down)\|bioid |
| MED30 | CDK8 | 0.89 | MI:0004(affinity chromatography technology)\|MI:0676(tandem affinity purification)\|MI:0007(anti tag coimmunoprecipitation)\|MI:0096(pull down)\|bioid |
| MED6 | CDK8 | 0.97 | Affinity Capture-MS\|Affinity Capture-Western\|MI:0004(affinity chromatography technology)\|MI:0676(tandem affinity purification)\|MI:0007(anti tag coimmunoprecipitation)\|MI:0096(pull down)\|bioid\|MI:0401(biochemical) |
| MED8 | CDK8 | 0.9 | MI:0492(in vitro)\|MI:0676(tandem affinity purification)\|MI:0004(affinity chromatography technology)\|MI:0007(anti tag coimmunoprecipitation)\|MI:0096(pull down)\|bioid\|MI:0401(biochemical) |
| MED9 | CDK8 | 0.9 | MI:0492(in vitro)\|Affinity Capture-MS\|MI:0007(anti tag coimmunoprecipitation)\|MI:0004(affinity chromatography technology)\|MI:0676(tandem affinity purification)\|MI:0096(pull down)\|bioid |
| MEF2C | MYLK2 | 0.72 | MI:0424(protein kinase assay)\|MI:0096(pull down) |
| MEPCE | PRPF6 | 0.72 | Affinity Capture-MS\|MI:0004(affinity chromatography technology) |
| MET | DNAJA3 | 0.85 | MI:0018(two hybrid)\|MI:0006(anti bait coimmunoprecipitation)\|MI:0096(pull down)\|MI:0663(confocal microscopy) |
| MET | GRB2 | 0.9 | MI:0492(in vitro)\|MI:0493(in vivo)\|MI:0018(two hybrid)\|Reconstituted Complex\|Affinity Capture-Western\|MI:0004(affinity chromatography technology)\|MI:0096(pull down)\|MI:0114(x-ray crystallography) |
| MET | STAT3 | 0.73 | MI:0493(in vivo)\|Affinity Capture-Western\|MI:0004(affinity chromatography technology) |
| MET | MET | 0.8 | MI:0492(in vitro)\|MI:0493(in vivo)\|MI:0415(enzymatic study)\|MI:0030(cross-linking study) |
| MET | PIK3R1 | 0.85 | MI:0492(in vitro)\|MI:0053(fluorescence polarization spectroscopy)\|MI:0096(pull down) |
| MET | PLCG1 | 0.86 | MI:0492(in vitro)\|MI:0053(fluorescence polarization spectroscopy)\|MI:0096(pull down) |
| MET | HGF | 0.9 | MI:0492(in vitro)\|MI:0493(in vivo)\|Reconstituted Complex\|Biochemical Activity\|Affinity Capture-Western\|MI:0114(x-ray crystallography)\|MI:0411(enzyme linked immunosorbent assay)\|MI:0055(fluorescent resonance energy transfer)\|MI:0415(enzymatic study)\|MI:0004(affinity chromatography technology)\|MI:0096(pull down)\|MI:0030(cross-linking study)\|MI:0440(saturation binding)\|MI:0826(x ray scattering) |
| MET | GAB1 | 0.95 | MI:0492(in vitro)\|MI:0493(in vivo)\|Affinity Capture-Western\|MI:0007(anti tag coimmunoprecipitation)\|MI:0004(affinity chromatography technology) |
| MET | SOS1 | 0.72 | Affinity Capture-Western\|MI:0004(affinity chromatography technology) |
| MET | PTPN1 | 0.88 | MI:0006(anti bait coimmunoprecipitation)\|MI:0434(phosphatase assay)\|MI:0030(cross-linking study) |
| MET | PLXNB3 | 0.72 | MI:0007(anti tag coimmunoprecipitation)\|MI:0006(anti bait coimmunoprecipitation) |
| MET | HSP90AA1 | 0.73 | MI:0004(affinity chromatography technology) |
| MET | SH2B1 | 0.75 | MI:0053(fluorescence polarization spectroscopy)\|MI:0096(pull down) |
| MET | SHD | 0.75 | MI:0053(fluorescence polarization spectroscopy)\|MI:0096(pull down) |
| MET | CRK | 0.87 | MI:0053(fluorescence polarization spectroscopy)\|MI:0004(affinity chromatography technology)\|MI:0096(pull down) |
| MET | FGR | 0.75 | MI:0053(fluorescence polarization spectroscopy)\|MI:0096(pull down) |
| MET | SH3BP2 | 0.75 | MI:0053(fluorescence polarization spectroscopy)\|MI:0096(pull down) |
| MET | PLCG2 | 0.75 | MI:0053(fluorescence polarization spectroscopy)\|MI:0096(pull down) |
| MET | TNS2 | 0.75 | MI:0053(fluorescence polarization spectroscopy)\|MI:0096(pull down) |
| MET | TNS1 | 0.75 | MI:0053(fluorescence polarization spectroscopy)\|MI:0096(pull down) |
| MET | BTK | 0.75 | MI:0053(fluorescence polarization spectroscopy)\|MI:0096(pull down) |
| MET | SH2D3C | 0.75 | MI:0053(fluorescence polarization spectroscopy)\|MI:0096(pull down) |
| MET | HCK | 0.75 | MI:0053(fluorescence polarization spectroscopy)\|MI:0096(pull down) |
| MET | VAV2 | 0.75 | MI:0053(fluorescence polarization spectroscopy)\|MI:0096(pull down) |
| MET | TNS3 | 0.75 | MI:0053(fluorescence polarization spectroscopy)\|MI:0096(pull down) |
| MET | HSH2D | 0.75 | MI:0053(fluorescence polarization spectroscopy)\|MI:0096(pull down) |
| MET | PIK3R3 | 0.75 | MI:0053(fluorescence polarization spectroscopy)\|MI:0096(pull down) |
| MET | GRB7 | 0.75 | MI:0053(fluorescence polarization spectroscopy)\|MI:0096(pull down) |
| MET | SHC3 | 0.75 | MI:0053(fluorescence polarization spectroscopy)\|MI:0096(pull down) |
| MET | STAP1 | 0.75 | MI:0053(fluorescence polarization spectroscopy)\|MI:0096(pull down) |
| MET | YES1 | 0.75 | MI:0053(fluorescence polarization spectroscopy)\|MI:0096(pull down) |
| MET | FES | 0.75 | MI:0053(fluorescence polarization spectroscopy)\|MI:0096(pull down) |
| MET | SOCS5 | 0.75 | MI:0053(fluorescence polarization spectroscopy)\|MI:0096(pull down) |
| MET | SYK | 0.75 | MI:0053(fluorescence polarization spectroscopy)\|MI:0096(pull down) |
| MET | ZAP70 | 0.75 | MI:0053(fluorescence polarization spectroscopy)\|MI:0096(pull down) |
| MET | SOCS2 | 0.75 | MI:0053(fluorescence polarization spectroscopy)\|MI:0096(pull down) |
| MET | ABL2 | 0.75 | MI:0053(fluorescence polarization spectroscopy)\|MI:0096(pull down) |
| MET | MATK | 0.75 | MI:0053(fluorescence polarization spectroscopy)\|MI:0096(pull down) |
| MET | ITK | 0.75 | MI:0053(fluorescence polarization spectroscopy)\|MI:0096(pull down) |
| MET | RASA1 | 0.75 | MI:0053(fluorescence polarization spectroscopy)\|MI:0096(pull down) |
| MET | TERT | 0.75 | MI:0055(fluorescent resonance energy transfer) |
| MET | STK11 | 0.75 | MI:0055(fluorescent resonance energy transfer) |
| MET | CCND2 | 0.75 | MI:0055(fluorescent resonance energy transfer) |
| MET | EPHA2 | 0.75 | MI:0055(fluorescent resonance energy transfer) |
| MET | CDKN2B | 0.75 | MI:0055(fluorescent resonance energy transfer) |
| MET | CDK6 | 0.75 | MI:0055(fluorescent resonance energy transfer) |
| MET | CDK4 | 0.75 | MI:0055(fluorescent resonance energy transfer) |
| MET | FGFR4 | 0.75 | MI:0055(fluorescent resonance energy transfer) |
| MET | ERBB3 | 0.82 | MI:0004(affinity chromatography technology)\|MI:0006(anti bait coimmunoprecipitation) |
| MET | LATS2 | 0.75 | MI:0055(fluorescent resonance energy transfer) |
| METTL1 | PRKDC | 0.72 | MI:0004(affinity chromatography technology)\|MI:0401(biochemical) |
| METTL14 | PRKDC | 0.72 | MI:0007(anti tag coimmunoprecipitation)\|MI:0004(affinity chromatography technology) |
| METTL3 | PRKDC | 0.72 | MI:0007(anti tag coimmunoprecipitation)\|MI:0004(affinity chromatography technology) |
| MLH1 | PRKDC | 0.72 | Affinity Capture-MS\|MI:0004(affinity chromatography technology) |
| MOB3C | ZBTB10 | 0.73 | MI:0018(two hybrid) |
| MRAP2 | CERK | 0.82 | MI:0004(affinity chromatography technology)\|MI:0007(anti tag coimmunoprecipitation) |
| MYC | PRKDC | 0.82 | MI:0676(tandem affinity purification)\|MI:0004(affinity chromatography technology) |
| MYC | PRPF6 | 0.87 | MI:0676(tandem affinity purification)\|MI:0006(anti bait coimmunoprecipitation)\|MI:0004(affinity chromatography technology) |
| MYC | SPEG | 0.72 | MI:0676(tandem affinity purification)\|MI:0004(affinity chromatography technology) |
| MYC | MET | 0.75 | MI:0055(fluorescent resonance energy transfer) |
| MYLK2 | FBXO11 | 0.85 | MI:0004(affinity chromatography technology)\|MI:0096(pull down)\|MI:0007(anti tag coimmunoprecipitation) |
| MYLK2 | RPS6KA4 | 0.85 | MI:0004(affinity chromatography technology)\|MI:0096(pull down)\|MI:0007(anti tag coimmunoprecipitation) |
| MYLK2 | SMARCA5 | 0.72 | MI:0007(anti tag coimmunoprecipitation)\|MI:0004(affinity chromatography technology) |
| MYLK2 | H3F3C | 0.72 | MI:0007(anti tag coimmunoprecipitation)\|MI:0004(affinity chromatography technology) |
| MYLK2 | NAP1L4 | 0.72 | MI:0007(anti tag coimmunoprecipitation)\|MI:0004(affinity chromatography technology) |
| MYLK2 | EBNA1BP2 | 0.72 | MI:0007(anti tag coimmunoprecipitation)\|MI:0004(affinity chromatography technology) |
| MYLK2 | CDC5L | 0.72 | MI:0007(anti tag coimmunoprecipitation)\|MI:0004(affinity chromatography technology) |
| MYLK2 | SLC25A1 | 0.72 | MI:0007(anti tag coimmunoprecipitation)\|MI:0004(affinity chromatography technology) |
| MYLK2 | DNAJA2 | 0.72 | MI:0007(anti tag coimmunoprecipitation)\|MI:0004(affinity chromatography technology) |
| MYLK2 | HNRNPCL1 | 0.72 | MI:0007(anti tag coimmunoprecipitation)\|MI:0004(affinity chromatography technology) |
| MYLK2 | HIST2H3C | 0.72 | MI:0007(anti tag coimmunoprecipitation)\|MI:0004(affinity chromatography technology) |
| MYLK2 | HIST1H3A | 0.72 | MI:0007(anti tag coimmunoprecipitation)\|MI:0004(affinity chromatography technology) |
| MYLK2 | H2AFZ | 0.72 | MI:0007(anti tag coimmunoprecipitation)\|MI:0004(affinity chromatography technology) |
| MYLK2 | H2AFV | 0.72 | MI:0007(anti tag coimmunoprecipitation)\|MI:0004(affinity chromatography technology) |
| MYLK2 | HNRNPA1L2 | 0.72 | MI:0007(anti tag coimmunoprecipitation)\|MI:0004(affinity chromatography technology) |
| MYLK2 | ADAR | 0.72 | MI:0007(anti tag coimmunoprecipitation)\|MI:0004(affinity chromatography technology) |
| MYLK2 | NAP1L1 | 0.72 | MI:0007(anti tag coimmunoprecipitation)\|MI:0004(affinity chromatography technology) |
| MYLK2 | AHSG | 0.72 | MI:0007(anti tag coimmunoprecipitation)\|MI:0004(affinity chromatography technology) |
| MYLK2 | FTSJ3 | 0.72 | MI:0007(anti tag coimmunoprecipitation)\|MI:0004(affinity chromatography technology) |
| MYLK2 | HSPB1 | 0.72 | MI:0007(anti tag coimmunoprecipitation)\|MI:0004(affinity chromatography technology) |
| MYLK2 | TECR | 0.72 | MI:0007(anti tag coimmunoprecipitation)\|MI:0004(affinity chromatography technology) |
| MYLK2 | HIST1H2AE | 0.72 | MI:0007(anti tag coimmunoprecipitation)\|MI:0004(affinity chromatography technology) |
| MYLK2 | DDX18 | 0.72 | MI:0007(anti tag coimmunoprecipitation)\|MI:0004(affinity chromatography technology) |
| MYLK2 | RCC1 | 0.72 | MI:0007(anti tag coimmunoprecipitation)\|MI:0004(affinity chromatography technology) |
| MYLK2 | SLC25A5 | 0.72 | MI:0007(anti tag coimmunoprecipitation)\|MI:0004(affinity chromatography technology) |
| MYLK2 | H3F3A | 0.72 | MI:0007(anti tag coimmunoprecipitation)\|MI:0004(affinity chromatography technology) |
| MYLK2 | DNAJA1 | 0.72 | MI:0007(anti tag coimmunoprecipitation)\|MI:0004(affinity chromatography technology) |
| MYLK2 | CCAR2 | 0.72 | MI:0007(anti tag coimmunoprecipitation)\|MI:0004(affinity chromatography technology) |
| MYLK2 | NOP56 | 0.72 | MI:0007(anti tag coimmunoprecipitation)\|MI:0004(affinity chromatography technology) |
| MYLK2 | PES1 | 0.72 | MI:0007(anti tag coimmunoprecipitation)\|MI:0004(affinity chromatography technology) |
| MYLK2 | HNRNPC | 0.72 | MI:0007(anti tag coimmunoprecipitation)\|MI:0004(affinity chromatography technology) |
| MYLK2 | UQCRH | 0.72 | MI:0007(anti tag coimmunoprecipitation)\|MI:0004(affinity chromatography technology) |
| MYLK2 | HNRNPA1 | 0.72 | MI:0007(anti tag coimmunoprecipitation)\|MI:0004(affinity chromatography technology) |
| MYLK2 | HSPA4 | 0.72 | MI:0007(anti tag coimmunoprecipitation)\|MI:0004(affinity chromatography technology) |
| MYLK2 | BAG2 | 0.72 | MI:0007(anti tag coimmunoprecipitation)\|MI:0004(affinity chromatography technology) |
| MYLK2 | TRIM54 | 0.72 | MI:0007(anti tag coimmunoprecipitation)\|MI:0018(two hybrid) |
| MYLK2 | HIST3H2A | 0.72 | MI:0007(anti tag coimmunoprecipitation)\|MI:0004(affinity chromatography technology) |
| MYLK2 | PSMC2 | 0.72 | MI:0007(anti tag coimmunoprecipitation)\|MI:0004(affinity chromatography technology) |
| MYLK2 | PSMD3 | 0.72 | MI:0007(anti tag coimmunoprecipitation)\|MI:0004(affinity chromatography technology) |
| MYLK2 | RALY | 0.72 | MI:0007(anti tag coimmunoprecipitation)\|MI:0004(affinity chromatography technology) |
| MYLK2 | UQCR10 | 0.72 | MI:0007(anti tag coimmunoprecipitation)\|MI:0004(affinity chromatography technology) |
| MYLK2 | WDR43 | 0.72 | MI:0007(anti tag coimmunoprecipitation)\|MI:0004(affinity chromatography technology) |
| MYLK2 | RRS1 | 0.72 | MI:0007(anti tag coimmunoprecipitation)\|MI:0004(affinity chromatography technology) |
| MYLK2 | YWHAG | 0.72 | MI:0007(anti tag coimmunoprecipitation)\|MI:0004(affinity chromatography technology) |
| MYLK2 | HIST1H2AC | 0.72 | MI:0007(anti tag coimmunoprecipitation)\|MI:0004(affinity chromatography technology) |
| MYLK2 | PSMC1 | 0.72 | MI:0007(anti tag coimmunoprecipitation)\|MI:0004(affinity chromatography technology) |
| MYLK2 | MYLK | 0.72 | MI:0007(anti tag coimmunoprecipitation)\|MI:0004(affinity chromatography technology) |
| MYLK2 | SAFB | 0.72 | MI:0007(anti tag coimmunoprecipitation)\|MI:0004(affinity chromatography technology) |
| MYLK2 | HIST4H4 | 0.72 | MI:0007(anti tag coimmunoprecipitation)\|MI:0004(affinity chromatography technology) |
| MYLK2 | CDC37 | 0.72 | MI:0007(anti tag coimmunoprecipitation)\|MI:0004(affinity chromatography technology) |
| MYLK2 | HIST3H3 | 0.72 | MI:0007(anti tag coimmunoprecipitation)\|MI:0004(affinity chromatography technology) |
| MYLK2 | NOP58 | 0.72 | MI:0007(anti tag coimmunoprecipitation)\|MI:0004(affinity chromatography technology) |
| MYLK2 | BRIX1 | 0.72 | MI:0007(anti tag coimmunoprecipitation)\|MI:0004(affinity chromatography technology) |
| MYLK2 | LMNB2 | 0.72 | MI:0007(anti tag coimmunoprecipitation)\|MI:0004(affinity chromatography technology) |
| MYLK2 | HSPA8 | 0.72 | MI:0007(anti tag coimmunoprecipitation)\|MI:0004(affinity chromatography technology) |
| MYLK2 | NAT10 | 0.72 | MI:0007(anti tag coimmunoprecipitation)\|MI:0004(affinity chromatography technology) |
| MYLK2 | TOP1 | 0.72 | MI:0007(anti tag coimmunoprecipitation)\|MI:0004(affinity chromatography technology) |
| MYLK2 | HNRNPA3 | 0.72 | MI:0007(anti tag coimmunoprecipitation)\|MI:0004(affinity chromatography technology) |
| MYLK2 | HSP90AB3P | 0.72 | MI:0007(anti tag coimmunoprecipitation)\|MI:0004(affinity chromatography technology) |
| NBN | PRKDC | 0.71 | MI:0492(in vitro)\|MI:0493(in vivo)\|MI:0096(pull down)\|MI:0004(affinity chromatography technology) |
| NCK1 | PTK2 | 0.88 | Reconstituted Complex\|Affinity Capture-Western\|MI:0004(affinity chromatography technology)\|MI:0096(pull down) |
| NCK1 | MET | 0.75 | MI:0053(fluorescence polarization spectroscopy)\|MI:0096(pull down) |
| NCK2 | PTK2 | 0.89 | MI:0493(in vivo)\|Reconstituted Complex\|Affinity Capture-Western\|MI:0004(affinity chromatography technology)\|MI:0096(pull down) |
| NCK2 | MET | 0.75 | MI:0053(fluorescence polarization spectroscopy)\|MI:0096(pull down) |
| NCK2 | TNK2 | 0.89 | MI:0004(affinity chromatography technology)\|MI:0007(anti tag coimmunoprecipitation) |
| NCOA6 | PRKDC | 0.87 | MI:0492(in vitro)\|Affinity Capture-MS\|Biochemical Activity\|MI:0415(enzymatic study)\|MI:0004(affinity chromatography technology) |
| NEDD4L | HGS | 0.75 | MI:0415(enzymatic study)\|MI:0004(affinity chromatography technology) |
| NEDD9 | PTK2 | 0.88 | MI:0492(in vitro)\|MI:0493(in vivo)\|MI:0018(two hybrid)\|Affinity Capture-Western\|MI:0004(affinity chromatography technology) |
| NEK4 | PRKDC | 0.72 | MI:0007(anti tag coimmunoprecipitation)\|MI:0004(affinity chromatography technology) |
| NEK4 | STK24 | 0.72 | MI:0007(anti tag coimmunoprecipitation)\|MI:0004(affinity chromatography technology) |
| NF2 | HGS | 0.9 | MI:0492(in vitro)\|MI:0493(in vivo)\|MI:0018(two hybrid)\|Reconstituted Complex\|Affinity Capture-Western\|MI:0004(affinity chromatography technology)\|MI:0096(pull down)\|MI:0012(bioluminescence resonance energy transfer)\|MI:0663(confocal microscopy)\|MI:0006(anti bait coimmunoprecipitation)\|MI:0090(protein complementation assay)\|MI:0055(fluorescent resonance energy transfer) |
| NF2 | MET | 0.75 | MI:0055(fluorescent resonance energy transfer) |
| NFATC2 | PRKCZ | 0.77 | MI:0493(in vivo)\|Affinity Capture-Western\|Biochemical Activity\|MI:0415(enzymatic study)\|MI:0004(affinity chromatography technology) |
| NFKB1 | PRKDC | 0.82 | MI:0007(anti tag coimmunoprecipitation)\|MI:0676(tandem affinity purification) |
| NFKB2 | MYLK2 | 0.72 | MI:0676(tandem affinity purification)\|MI:0007(anti tag coimmunoprecipitation) |
| NFKB2 | PRKDC | 0.72 | MI:0676(tandem affinity purification)\|MI:0007(anti tag coimmunoprecipitation) |
| NHEJ1 | PRKDC | 0.75 | MI:0007(anti tag coimmunoprecipitation)\|MI:0415(enzymatic study) |
| NIPSNAP2 | PRKDC | 0.72 | MI:0007(anti tag coimmunoprecipitation)\|MI:0004(affinity chromatography technology) |
| NKAPD1 | CLK2 | 0.82 | MI:0004(affinity chromatography technology)\|MI:0007(anti tag coimmunoprecipitation) |
| NMI | HGS | 0.74 | MI:0018(two hybrid) |
| NOP16 | CERK | 0.72 | MI:0030(cross-linking study)\|bioid |
| NOTCH1 | PRKDC | 0.76 | MI:0004(affinity chromatography technology)\|MI:0676(tandem affinity purification)\|MI:0007(anti tag coimmunoprecipitation) |
| NOTCH1 | PRPF6 | 0.72 | MI:0004(affinity chromatography technology)\|MI:0676(tandem affinity purification) |
| NPHP1 | TNK2 | 0.84 | MI:0004(affinity chromatography technology)\|MI:0018(two hybrid)\|MI:0096(pull down) |
| NR1H4 | PRKDC | 0.75 | MI:0004(affinity chromatography technology)\|MI:0096(pull down)\|MI:0007(anti tag coimmunoprecipitation) |
| NUCB1 | PRKDC | 0.72 | MI:0004(affinity chromatography technology)\|MI:0401(biochemical) |
| OCIAD2 | SIK3 | 0.72 | MI:0030(cross-linking study)\|bioid |
| PARD3 | PRKCZ | 0.95 | Affinity Capture-Western\|MI:0004(affinity chromatography technology)\|MI:0007(anti tag coimmunoprecipitation) |
| PARD6A | PRKCZ | 0.99 | MI:0492(in vitro)\|MI:0493(in vivo)\|Reconstituted Complex\|MI:0007(anti tag coimmunoprecipitation)\|MI:0022(colocalization by immunostaining)\|MI:0018(two hybrid)\|MI:0004(affinity chromatography technology)\|MI:0428(imaging technique)\|MI:0096(pull down) |
| PARD6B | PRKCZ | 0.9 | MI:0492(in vitro)\|MI:0493(in vivo)\|Reconstituted Complex\|MI:0018(two hybrid)\|MI:0096(pull down)\|MI:0019(coimmunoprecipitation)\|MI:0007(anti tag coimmunoprecipitation)\|MI:0004(affinity chromatography technology) |
| PARD6G | PRKCZ | 0.95 | MI:0492(in vitro)\|MI:0018(two hybrid)\|MI:0007(anti tag coimmunoprecipitation)\|MI:0004(affinity chromatography technology)\|MI:0096(pull down) |
| PARP1 | CDK8 | 0.78 | MI:0004(affinity chromatography technology)\|MI:0096(pull down) |
| PAWR | PRKCZ | 0.89 | MI:0492(in vitro)\|MI:0493(in vivo)\|MI:0018(two hybrid)\|Reconstituted Complex\|Affinity Capture-Western\|MI:0004(affinity chromatography technology)\|MI:0096(pull down) |
| PCBP1 | MET | 0.83 | MI:0004(affinity chromatography technology)\|MI:0007(anti tag coimmunoprecipitation) |
| PDCD10 | STK24 | 0.9 | Affinity Capture-MS\|MI:0007(anti tag coimmunoprecipitation)\|MI:0018(two hybrid)\|MI:0004(affinity chromatography technology)\|MI:0006(anti bait coimmunoprecipitation)\|MI:0676(tandem affinity purification)\|MI:0096(pull down)\|MI:0401(biochemical) |
| PDHB | PDK2 | 0.83 | MI:0004(affinity chromatography technology)\|MI:0007(anti tag coimmunoprecipitation) |
| PDK2 | PDHX | 0.86 | MI:0492(in vitro)\|Reconstituted Complex\|MI:0096(pull down) |
| PDK2 | PDHA1 | 0.86 | MI:0492(in vitro)\|MI:0424(protein kinase assay)\|bioid |
| PDK2 | PDK1 | 0.9 | MI:0492(in vitro)\|Reconstituted Complex\|MI:0096(pull down)\|MI:0004(affinity chromatography technology)\|MI:0007(anti tag coimmunoprecipitation)\|bioid |
| PDK2 | PDK2 | 0.7 | MI:0492(in vitro) |
| PDK3 | PDK2 | 0.85 | MI:0004(affinity chromatography technology)\|MI:0096(pull down)\|MI:0007(anti tag coimmunoprecipitation) |
| PDK4 | PDK2 | 0.82 | MI:0004(affinity chromatography technology)\|MI:0007(anti tag coimmunoprecipitation) |
| PDPK1 | PRKCZ | 0.9 | MI:0492(in vitro)\|MI:0493(in vivo)\|MI:0018(two hybrid)\|Affinity Capture-Western\|Biochemical Activity\|Reconstituted Complex\|MI:0415(enzymatic study)\|MI:0004(affinity chromatography technology)\|MI:0096(pull down) |
| PELP1 | HGS | 0.76 | MI:0492(in vitro)\|MI:0493(in vivo)\|MI:0018(two hybrid)\|MI:0004(affinity chromatography technology)\|MI:0096(pull down) |
| PFKFB3 | PFKFB4 | 0.82 | MI:0004(affinity chromatography technology)\|MI:0007(anti tag coimmunoprecipitation) |
| PFKFB4 | PFKFB1 | 0.82 | MI:0018(two hybrid)\|MI:0004(affinity chromatography technology) |
| PHKG2 | PRKDC | 0.72 | MI:0676(tandem affinity purification)\|MI:0004(affinity chromatography technology) |
| PIAS1 | PTK2 | 0.79 | MI:0492(in vitro)\|MI:0493(in vivo)\|MI:0018(two hybrid)\|MI:0428(imaging technique) |
| PIK3CD | PIK3R1 | 0.97 | MI:0493(in vivo)\|Reconstituted Complex\|Affinity Capture-Western\|MI:0004(affinity chromatography technology)\|MI:0096(pull down)\|MI:0006(anti bait coimmunoprecipitation)\|MI:0663(confocal microscopy)\|MI:0071(molecular sieving)\|MI:0424(protein kinase assay)\|MI:0007(anti tag coimmunoprecipitation)\|MI:0018(two hybrid) |
| PIK3CD | HRAS | 0.91 | MI:0493(in vivo)\|MI:0018(two hybrid)\|MI:0006(anti bait coimmunoprecipitation) |
| PIK3CD | RALY | 0.73 | MI:0018(two hybrid) |
| PIK3CD | PIK3CG | 0.78 | Reconstituted Complex\|Affinity Capture-Western\|MI:0004(affinity chromatography technology)\|MI:0096(pull down) |
| PIK3CD | PIK3R3 | 0.87 | MI:0004(affinity chromatography technology)\|MI:0007(anti tag coimmunoprecipitation)\|MI:0018(two hybrid) |
| PIK3R2 | PIK3CD | 0.88 | MI:0493(in vivo)\|Reconstituted Complex\|Affinity Capture-Western\|MI:0004(affinity chromatography technology)\|MI:0096(pull down) |
| PIK3R2 | MET | 0.75 | MI:0053(fluorescence polarization spectroscopy)\|MI:0096(pull down) |
| PINK1 | PRKDC | 0.73 | MI:0004(affinity chromatography technology) |
| PKD1 | PTK2 | 0.77 | MI:0492(in vitro)\|MI:0493(in vivo)\|MI:0004(affinity chromatography technology) |
| PLEC | PRKCH | 0.72 | MI:0030(cross-linking study)\|bioid |
| PLEKHO2 | MAP3K6 | 0.83 | MI:0004(affinity chromatography technology)\|MI:0007(anti tag coimmunoprecipitation) |
| PLK1 | PRKDC | 0.78 | MI:0096(pull down)\|MI:0004(affinity chromatography technology) |
| PLXNB1 | MET | 0.92 | MI:0492(in vitro)\|MI:0493(in vivo)\|MI:0007(anti tag coimmunoprecipitation)\|MI:0006(anti bait coimmunoprecipitation) |
| PLXNB2 | MET | 0.72 | MI:0007(anti tag coimmunoprecipitation)\|MI:0006(anti bait coimmunoprecipitation) |
| PMEPA1 | HGS | 0.75 | MI:0004(affinity chromatography technology)\|MI:0096(pull down)\|MI:0018(two hybrid) |
| PMS2 | PRKDC | 0.72 | Affinity Capture-MS\|MI:0004(affinity chromatography technology) |
| POLR2A | CDK8 | 0.98 | MI:0492(in vitro)\|MI:0493(in vivo)\|Co-fractionation\|Affinity Capture-Western\|MI:0004(affinity chromatography technology)\|MI:0415(enzymatic study)\|MI:0401(biochemical) |
| POT1 | PRKDC | 0.72 | MI:0004(affinity chromatography technology)\|MI:0007(anti tag coimmunoprecipitation) |
| PPP1CB | PTK2 | 0.92 | MI:0492(in vitro)\|MI:0493(in vivo)\|Affinity Capture-Western\|MI:0004(affinity chromatography technology) |
| PPP3CA | SIK3 | 0.83 | MI:0004(affinity chromatography technology)\|MI:0007(anti tag coimmunoprecipitation) |
| PPP3CC | SIK3 | 0.83 | MI:0004(affinity chromatography technology)\|MI:0007(anti tag coimmunoprecipitation) |
| PRCP | STK24 | 0.72 | MI:0004(affinity chromatography technology)\|MI:0401(biochemical) |
| PRKAA2 | MAP3K6 | 0.72 | MI:0007(anti tag coimmunoprecipitation)\|MI:0004(affinity chromatography technology) |
| PRKAA2 | PRKDC | 0.78 | MI:0096(pull down)\|MI:0004(affinity chromatography technology) |
| PRKAB2 | MAP3K6 | 0.72 | MI:0007(anti tag coimmunoprecipitation)\|MI:0004(affinity chromatography technology) |
| PRKAB2 | MAP3K15 | 0.72 | MI:0007(anti tag coimmunoprecipitation)\|MI:0004(affinity chromatography technology) |
| PRKAB2 | PRKDC | 0.72 | MI:0007(anti tag coimmunoprecipitation)\|MI:0004(affinity chromatography technology) |
| PRKACA | AKAP14 | 0.85 | MI:0492(in vitro)\|MI:0493(in vivo)\|MI:0004(affinity chromatography technology)\|MI:0007(anti tag coimmunoprecipitation) |
| PRKACA | SIK3 | 0.83 | MI:0018(two hybrid)\|MI:0004(affinity chromatography technology) |
| PRKCA | PRKCH | 0.82 | MI:0004(affinity chromatography technology)\|MI:0007(anti tag coimmunoprecipitation) |
| PRKCH | CDK2 | 0.89 | MI:0493(in vivo)\|Affinity Capture-Western\|MI:0004(affinity chromatography technology) |
| PRKCH | OCLN | 0.72 | MI:0096(pull down)\|MI:0424(protein kinase assay) |
| PRKCZ | PRKCA | 0.78 | MI:0492(in vitro)\|MI:0493(in vivo)\|MI:0004(affinity chromatography technology) |
| PRKCZ | NCF1 | 0.83 | MI:0492(in vitro)\|MI:0415(enzymatic study)\|MI:0096(pull down)\|MI:0006(anti bait coimmunoprecipitation)\|MI:0416(fluorescence microscopy) |
| PRKCZ | YWHAB | 0.89 | MI:0492(in vitro)\|MI:0493(in vivo)\|Biochemical Activity\|Affinity Capture-Western\|MI:0415(enzymatic study)\|MI:0004(affinity chromatography technology)\|MI:0018(two hybrid) |
| PRKCZ | AKT3 | 0.89 | MI:0492(in vitro)\|MI:0493(in vivo)\|MI:0018(two hybrid)\|Affinity Capture-Western\|Biochemical Activity\|MI:0415(enzymatic study)\|MI:0004(affinity chromatography technology)\|MI:0428(imaging technique) |
| PRKCZ | PEBP1 | 0.73 | MI:0492(in vitro)\|MI:0493(in vivo)\|MI:0096(pull down) |
| PRKCZ | CSNK2B | 0.88 | MI:0492(in vitro)\|MI:0493(in vivo)\|Affinity Capture-Western\|MI:0004(affinity chromatography technology)\|MI:0007(anti tag coimmunoprecipitation) |
| PRKCZ | PRKCZ | 0.78 | MI:0493(in vivo)\|MI:0415(enzymatic study)\|MI:0004(affinity chromatography technology) |
| PRKCZ | NUMB | 0.85 | MI:0492(in vitro)\|MI:0007(anti tag coimmunoprecipitation)\|MI:0059(gst pull down)\|MI:0424(protein kinase assay)\|MI:0096(pull down)\|MI:0415(enzymatic study) |
| PRKCZ | KRT10 | 0.73 | MI:0493(in vivo)\|Affinity Capture-Western\|MI:0004(affinity chromatography technology) |
| PRKCZ | RELA | 0.89 | MI:0492(in vitro)\|MI:0493(in vivo)\|Biochemical Activity\|Affinity Capture-Western\|MI:0045(experimental interaction detection)\|MI:0415(enzymatic study)\|MI:0004(affinity chromatography technology) |
| PRKCZ | NCL | 0.79 | MI:0492(in vitro)\|MI:0493(in vivo)\|Biochemical Activity\|MI:0415(enzymatic study) |
| PRKCZ | AKT1 | 0.9 | MI:0492(in vitro)\|MI:0493(in vivo)\|Affinity Capture-Western\|MI:0006(anti bait coimmunoprecipitation)\|MI:0424(protein kinase assay)\|MI:0004(affinity chromatography technology)\|MI:0415(enzymatic study) |
| PRKCZ | RAF1 | 0.88 | MI:0493(in vivo)\|Affinity Capture-Western\|Reconstituted Complex\|MI:0004(affinity chromatography technology)\|MI:0096(pull down) |
| PRKCZ | RPS6KB1 | 0.72 | Affinity Capture-Western\|MI:0004(affinity chromatography technology) |
| PRKCZ | YWHAG | 0.72 | Affinity Capture-Western\|MI:0004(affinity chromatography technology) |
| PRKCZ | YWHAQ | 0.79 | Affinity Capture-Western\|Reconstituted Complex\|Biochemical Activity\|MI:0415(enzymatic study)\|MI:0004(affinity chromatography technology)\|MI:0096(pull down) |
| PRKCZ | YWHAH | 0.72 | Affinity Capture-Western\|MI:0004(affinity chromatography technology) |
| PRKCZ | PRKCI | 0.89 | MI:0019(coimmunoprecipitation)\|MI:0004(affinity chromatography technology)\|MI:0007(anti tag coimmunoprecipitation)\|MI:0006(anti bait coimmunoprecipitation)\|MI:0096(pull down) |
| PRKCZ | PRKCQ | 0.77 | MI:0006(anti bait coimmunoprecipitation)\|MI:0018(two hybrid)\|MI:0424(protein kinase assay) |
| PRKCZ | HSP90AA1 | 0.83 | MI:0018(two hybrid)\|MI:0004(affinity chromatography technology) |
| PRKCZ | PIAS4 | 0.83 | MI:0018(two hybrid)\|MI:0004(affinity chromatography technology)\|MI:0415(enzymatic study) |
| PRKCZ | CFL1 | 0.73 | MI:0004(affinity chromatography technology) |
| PRKCZ | NPM1 | 0.83 | MI:0004(affinity chromatography technology)\|MI:0007(anti tag coimmunoprecipitation) |
| PRKCZ | LLGL2 | 0.85 | MI:0004(affinity chromatography technology)\|MI:0007(anti tag coimmunoprecipitation)\|MI:0096(pull down) |
| PRKCZ | DYNLL1 | 0.72 | MI:0004(affinity chromatography technology)\|MI:0007(anti tag coimmunoprecipitation) |
| PRKCZ | NIPSNAP1 | 0.82 | MI:0004(affinity chromatography technology)\|MI:0007(anti tag coimmunoprecipitation) |
| PRKCZ | NPM3 | 0.82 | MI:0004(affinity chromatography technology)\|MI:0007(anti tag coimmunoprecipitation) |
| PRKCZ | KEAP1 | 0.82 | MI:0004(affinity chromatography technology)\|MI:0007(anti tag coimmunoprecipitation) |
| PRKCZ | PRDX4 | 0.78 | MI:0004(affinity chromatography technology)\|MI:0096(pull down) |
| PRKCZ | CDC42BPB | 0.82 | MI:0004(affinity chromatography technology)\|MI:0007(anti tag coimmunoprecipitation) |
| PRKCZ | BAG2 | 0.82 | MI:0004(affinity chromatography technology)\|MI:0007(anti tag coimmunoprecipitation) |
| PRKCZ | BLVRA | 0.7 | MI:0004(affinity chromatography technology)\|MI:0096(pull down)\|MI:0415(enzymatic study) |
| PRKCZ | SLC25A5 | 0.82 | MI:0004(affinity chromatography technology)\|MI:0007(anti tag coimmunoprecipitation) |
| PRKCZ | MRPL53 | 0.82 | MI:0004(affinity chromatography technology)\|MI:0007(anti tag coimmunoprecipitation) |
| PRKCZ | CDC37 | 0.82 | MI:0004(affinity chromatography technology)\|MI:0007(anti tag coimmunoprecipitation) |
| PRKCZ | WDR26 | 0.82 | MI:0004(affinity chromatography technology)\|MI:0007(anti tag coimmunoprecipitation) |
| PRKCZ | EIF5A | 0.78 | MI:0004(affinity chromatography technology)\|MI:0096(pull down) |
| PRKCZ | HSPB1 | 0.82 | MI:0004(affinity chromatography technology)\|MI:0007(anti tag coimmunoprecipitation) |
| PRKCZ | NIPSNAP2 | 0.85 | MI:0004(affinity chromatography technology)\|MI:0096(pull down)\|MI:0007(anti tag coimmunoprecipitation) |
| PRKCZ | NAP1L1 | 0.82 | MI:0004(affinity chromatography technology)\|MI:0007(anti tag coimmunoprecipitation) |
| PRKCZ | TUBB6 | 0.82 | MI:0004(affinity chromatography technology)\|MI:0007(anti tag coimmunoprecipitation) |
| PRKCZ | SLC25A3 | 0.82 | MI:0004(affinity chromatography technology)\|MI:0007(anti tag coimmunoprecipitation) |
| PRKCZ | MRPL12 | 0.82 | MI:0004(affinity chromatography technology)\|MI:0007(anti tag coimmunoprecipitation) |
| PRKCZ | AGER | 0.72 | MI:0007(anti tag coimmunoprecipitation)\|MI:0006(anti bait coimmunoprecipitation) |
| PRKCZ | GLG1 | 0.72 | MI:0007(anti tag coimmunoprecipitation)\|MI:0004(affinity chromatography technology) |
| PRKCZ | AP3S1 | 0.72 | MI:0007(anti tag coimmunoprecipitation)\|MI:0004(affinity chromatography technology) |
| PRKCZ | FAT1 | 0.72 | MI:0007(anti tag coimmunoprecipitation)\|MI:0004(affinity chromatography technology) |
| PRKCZ | RCN2 | 0.72 | MI:0007(anti tag coimmunoprecipitation)\|MI:0004(affinity chromatography technology) |
| PRKCZ | FBL | 0.72 | MI:0007(anti tag coimmunoprecipitation)\|MI:0004(affinity chromatography technology) |
| PRKCZ | CALU | 0.84 | MI:0096(pull down)\|MI:0007(anti tag coimmunoprecipitation)\|MI:0004(affinity chromatography technology) |
| PRKCZ | PSME3 | 0.84 | MI:0096(pull down)\|MI:0007(anti tag coimmunoprecipitation)\|MI:0004(affinity chromatography technology) |
| PRKCZ | UBE2O | 0.72 | MI:0007(anti tag coimmunoprecipitation)\|MI:0004(affinity chromatography technology) |
| PRKCZ | TRIM28 | 0.72 | MI:0007(anti tag coimmunoprecipitation)\|MI:0004(affinity chromatography technology) |
| PRKCZ | DDA1 | 0.72 | MI:0007(anti tag coimmunoprecipitation)\|MI:0004(affinity chromatography technology) |
| PRKCZ | AIFM1 | 0.72 | MI:0007(anti tag coimmunoprecipitation)\|MI:0004(affinity chromatography technology) |
| PRKCZ | LOC100290337 | 0.72 | MI:0007(anti tag coimmunoprecipitation)\|MI:0004(affinity chromatography technology) |
| PRKCZ | PRKACB | 0.72 | MI:0007(anti tag coimmunoprecipitation)\|MI:0004(affinity chromatography technology) |
| PRKCZ | NEDD8 | 0.72 | MI:0007(anti tag coimmunoprecipitation)\|MI:0004(affinity chromatography technology) |
| PRKCZ | RPUSD4 | 0.72 | MI:0007(anti tag coimmunoprecipitation)\|MI:0004(affinity chromatography technology) |
| PRKCZ | AP2M1 | 0.72 | MI:0007(anti tag coimmunoprecipitation)\|MI:0004(affinity chromatography technology) |
| PRKCZ | EPB41L4B | 0.72 | MI:0007(anti tag coimmunoprecipitation)\|MI:0004(affinity chromatography technology) |
| PRKCZ | SLC25A6 | 0.72 | MI:0007(anti tag coimmunoprecipitation)\|MI:0004(affinity chromatography technology) |
| PRKCZ | SLC25A4 | 0.72 | MI:0007(anti tag coimmunoprecipitation)\|MI:0004(affinity chromatography technology) |
| PRKCZ | PRKAR1A | 0.72 | MI:0007(anti tag coimmunoprecipitation)\|MI:0004(affinity chromatography technology) |
| PRKCZ | MRPS2 | 0.72 | MI:0007(anti tag coimmunoprecipitation)\|MI:0004(affinity chromatography technology) |
| PRKCZ | DCAF1 | 0.72 | MI:0007(anti tag coimmunoprecipitation)\|MI:0004(affinity chromatography technology) |
| PRKCZ | NOC2L | 0.72 | MI:0007(anti tag coimmunoprecipitation)\|MI:0004(affinity chromatography technology) |
| PRKCZ | GTF3C5 | 0.72 | MI:0007(anti tag coimmunoprecipitation)\|MI:0004(affinity chromatography technology) |
| PRKCZ | HSPA8 | 0.72 | MI:0007(anti tag coimmunoprecipitation)\|MI:0004(affinity chromatography technology) |
| PRKCZ | CPT1A | 0.72 | MI:0007(anti tag coimmunoprecipitation)\|MI:0004(affinity chromatography technology) |
| PRKCZ | DNAJA2 | 0.72 | MI:0007(anti tag coimmunoprecipitation)\|MI:0004(affinity chromatography technology) |
| PRKCZ | POLD1 | 0.72 | MI:0007(anti tag coimmunoprecipitation)\|MI:0004(affinity chromatography technology) |
| PRKCZ | NAP1L4 | 0.72 | MI:0007(anti tag coimmunoprecipitation)\|MI:0004(affinity chromatography technology) |
| PRKCZ | ADH1C | 0.72 | MI:0007(anti tag coimmunoprecipitation)\|MI:0004(affinity chromatography technology) |
| PRKCZ | CAD | 0.72 | MI:0007(anti tag coimmunoprecipitation)\|MI:0004(affinity chromatography technology) |
| PRKCZ | HADHB | 0.84 | MI:0096(pull down)\|MI:0007(anti tag coimmunoprecipitation)\|MI:0004(affinity chromatography technology) |
| PRKCZ | PPFIBP1 | 0.72 | MI:0007(anti tag coimmunoprecipitation)\|MI:0004(affinity chromatography technology) |
| PRKCZ | TUBB4B | 0.72 | MI:0007(anti tag coimmunoprecipitation)\|MI:0004(affinity chromatography technology) |
| PRKCZ | CSNK2A1 | 0.72 | MI:0007(anti tag coimmunoprecipitation)\|MI:0004(affinity chromatography technology) |
| PRKCZ | HADHA | 0.84 | MI:0096(pull down)\|MI:0007(anti tag coimmunoprecipitation)\|MI:0004(affinity chromatography technology) |
| PRKCZ | ITSN2 | 0.72 | MI:0007(anti tag coimmunoprecipitation)\|MI:0004(affinity chromatography technology) |
| PRKCZ | HSPA6 | 0.72 | MI:0007(anti tag coimmunoprecipitation)\|MI:0004(affinity chromatography technology) |
| PRKCZ | HUWE1 | 0.72 | MI:0007(anti tag coimmunoprecipitation)\|MI:0004(affinity chromatography technology) |
| PRKCZ | DNAJA1 | 0.72 | MI:0007(anti tag coimmunoprecipitation)\|MI:0004(affinity chromatography technology) |
| PRKCZ | CAMSAP3 | 0.72 | MI:0007(anti tag coimmunoprecipitation)\|MI:0004(affinity chromatography technology) |
| PRKCZ | TIMM50 | 0.72 | MI:0007(anti tag coimmunoprecipitation)\|MI:0004(affinity chromatography technology) |
| PRKCZ | AP2A2 | 0.72 | MI:0007(anti tag coimmunoprecipitation)\|MI:0004(affinity chromatography technology) |
| PRKCZ | ASPH | 0.72 | MI:0007(anti tag coimmunoprecipitation)\|MI:0004(affinity chromatography technology) |
| PRKCZ | TRIM26 | 0.72 | MI:0007(anti tag coimmunoprecipitation)\|MI:0004(affinity chromatography technology) |
| PRKCZ | FARP2 | 0.72 | MI:0007(anti tag coimmunoprecipitation)\|MI:0004(affinity chromatography technology) |
| PRKCZ | HSPA7 | 0.72 | MI:0007(anti tag coimmunoprecipitation)\|MI:0004(affinity chromatography technology) |
| PRKCZ | POLRMT | 0.72 | MI:0007(anti tag coimmunoprecipitation)\|MI:0004(affinity chromatography technology) |
| PRKCZ | IPO5 | 0.84 | MI:0096(pull down)\|MI:0007(anti tag coimmunoprecipitation)\|MI:0004(affinity chromatography technology) |
| PRKCZ | TUBB | 0.72 | MI:0007(anti tag coimmunoprecipitation)\|MI:0004(affinity chromatography technology) |
| PRKCZ | MAP1LC3B | 0.7 | MI:0004(affinity chromatography technology)\|MI:0096(pull down)\|MI:0415(enzymatic study) |
| PRKDC | LYN | 0.75 | MI:0492(in vitro)\|Reconstituted Complex\|MI:0096(pull down) |
| PRKDC | JUN | 0.77 | MI:0492(in vitro)\|MI:0415(enzymatic study)\|MI:0006(anti bait coimmunoprecipitation) |
| PRKDC | PARP1 | 0.84 | MI:0493(in vivo)\|MI:0006(anti bait coimmunoprecipitation)\|MI:0004(affinity chromatography technology) |
| PRKDC | PCNA | 0.73 | MI:0492(in vitro)\|Affinity Capture-MS\|MI:0004(affinity chromatography technology) |
| PRKDC | PRKCD | 0.75 | MI:0492(in vitro)\|MI:0007(anti tag coimmunoprecipitation) |
| PRKDC | RPA1 | 0.89 | MI:0492(in vitro)\|Reconstituted Complex\|MI:0019(coimmunoprecipitation)\|MI:0096(pull down)\|MI:0004(affinity chromatography technology) |
| PRKDC | RPA2 | 0.9 | MI:0492(in vitro)\|Affinity Capture-Western\|Reconstituted Complex\|MI:0019(coimmunoprecipitation)\|MI:0004(affinity chromatography technology)\|MI:0096(pull down)\|MI:0415(enzymatic study) |
| PRKDC | ABL1 | 0.75 | MI:0492(in vitro)\|Reconstituted Complex\|MI:0096(pull down) |
| PRKDC | TP53 | 0.88 | MI:0492(in vitro)\|MI:0493(in vivo)\|Protein-peptide\|Biochemical Activity\|MI:0415(enzymatic study)\|MI:0004(affinity chromatography technology) |
| PRKDC | SRF | 0.79 | MI:0492(in vitro)\|MI:0493(in vivo)\|MI:0415(enzymatic study)\|MI:0007(anti tag coimmunoprecipitation) |
| PRKDC | CHUK | 0.78 | MI:0493(in vivo)\|Biochemical Activity\|Reconstituted Complex\|MI:0415(enzymatic study)\|MI:0096(pull down) |
| PRKDC | LIG4 | 0.88 | MI:0492(in vitro)\|Protein-peptide\|MI:0415(enzymatic study)\|MI:0004(affinity chromatography technology)\|MI:0006(anti bait coimmunoprecipitation) |
| PRKDC | XPA | 0.77 | MI:0492(in vitro)\|MI:0415(enzymatic study)\|MI:0007(anti tag coimmunoprecipitation) |
| PRKDC | CIB1 | 0.88 | MI:0492(in vitro)\|MI:0493(in vivo)\|MI:0018(two hybrid)\|Reconstituted Complex\|MI:0096(pull down)\|MI:0004(affinity chromatography technology) |
| PRKDC | HSP90AA1 | 0.75 | MI:0492(in vitro)\|MI:0004(affinity chromatography technology) |
| PRKDC | HSF1 | 0.75 | MI:0492(in vitro)\|Reconstituted Complex\|MI:0096(pull down) |
| PRKDC | AR | 0.86 | Affinity Capture-MS\|MI:0004(affinity chromatography technology)\|MI:0006(anti bait coimmunoprecipitation) |
| PRKDC | BRCA1 | 0.84 | Protein-peptide\|MI:0004(affinity chromatography technology)\|MI:0096(pull down) |
| PRKDC | CHEK1 | 0.87 | Protein-peptide\|Reconstituted Complex\|MI:0096(pull down) |
| PRKDC | MRE11 | 0.82 | Protein-peptide\|MI:0004(affinity chromatography technology) |
| PRKDC | AKT2 | 0.75 | Biochemical Activity\|MI:0415(enzymatic study) |
| PRKDC | ILF3 | 0.88 | MI:0025(copurification)\|Affinity Capture-Western\|MI:0004(affinity chromatography technology)\|MI:0401(biochemical) |
| PRKDC | MAPK9 | 0.83 | Biochemical Activity\|MI:0415(enzymatic study)\|MI:0004(affinity chromatography technology) |
| PRKDC | PIDD1 | 0.87 | MI:0006(anti bait coimmunoprecipitation)\|MI:0007(anti tag coimmunoprecipitation)\|MI:0096(pull down)\|MI:0004(affinity chromatography technology) |
| PRKDC | AIRE | 0.72 | MI:0006(anti bait coimmunoprecipitation)\|MI:0004(affinity chromatography technology) |
| PRKDC | TOP2A | 0.72 | MI:0006(anti bait coimmunoprecipitation)\|MI:0007(anti tag coimmunoprecipitation) |
| PRKDC | ERG | 0.88 | MI:0096(pull down)\|MI:0402(chromatin immunoprecipitation assay)\|MI:0006(anti bait coimmunoprecipitation)\|MI:0007(anti tag coimmunoprecipitation)\|MI:0004(affinity chromatography technology) |
| PRKDC | PPP6C | 0.85 | MI:0004(affinity chromatography technology)\|MI:0096(pull down)\|MI:0007(anti tag coimmunoprecipitation) |
| PRKDC | PPP6R2 | 0.85 | MI:0004(affinity chromatography technology)\|MI:0096(pull down)\|MI:0007(anti tag coimmunoprecipitation) |
| PRKDC | RBBP8 | 0.73 | MI:0004(affinity chromatography technology) |
| PRKDC | RUVBL2 | 0.73 | MI:0004(affinity chromatography technology) |
| PRKDC | MCM2 | 0.73 | MI:0004(affinity chromatography technology) |
| PRKDC | PRDX1 | 0.72 | MI:0004(affinity chromatography technology)\|MI:0401(biochemical) |
| PRKDC | PRPF8 | 0.82 | MI:0004(affinity chromatography technology)\|MI:0401(biochemical) |
| PRKDC | CTPS2 | 0.72 | MI:0004(affinity chromatography technology)\|MI:0401(biochemical) |
| PRKDC | EFTUD2 | 0.82 | MI:0004(affinity chromatography technology)\|MI:0401(biochemical) |
| PRKDC | GTF2I | 0.72 | MI:0004(affinity chromatography technology)\|MI:0401(biochemical) |
| PRKDC | HIF1A | 0.73 | MI:0004(affinity chromatography technology) |
| PRKDC | XRCC1 | 0.73 | MI:0004(affinity chromatography technology) |
| PRKDC | NRXN1 | 0.72 | MI:0030(cross-linking study)\|bioid |
| PRKDC | GTPBP4 | 0.72 | MI:0030(cross-linking study)\|bioid |
| PRKDC | RNF8 | 0.72 | MI:0030(cross-linking study)\|bioid |
| PRKDC | TBC1D28 | 0.72 | MI:0030(cross-linking study)\|bioid |
| PRKDC | ZNF840P | 0.72 | MI:0030(cross-linking study)\|bioid |
| PRKDC | ANXA2 | 0.72 | MI:0030(cross-linking study)\|bioid |
| PRKDC | KIAA1217 | 0.72 | MI:0030(cross-linking study)\|bioid |
| PRKDC | LAMC3 | 0.72 | MI:0030(cross-linking study)\|bioid |
| PRKDC | CPN1 | 0.72 | MI:0030(cross-linking study)\|bioid |
| PRKDC | SYNE2 | 0.72 | MI:0030(cross-linking study)\|bioid |
| PRKDC | FAM161A | 0.72 | MI:0030(cross-linking study)\|bioid |
| PRKDC | SYNJ2 | 0.72 | MI:0030(cross-linking study)\|bioid |
| PRKDC | PWWP2B | 0.72 | MI:0030(cross-linking study)\|bioid |
| PRKG1 | PTK2 | 0.82 | MI:0004(affinity chromatography technology)\|MI:0007(anti tag coimmunoprecipitation) |
| PRMT1 | SPEG | 0.73 | MI:0018(two hybrid) |
| PRMT5 | FAM47E | 0.78 | MI:0006(anti bait coimmunoprecipitation)\|MI:0096(pull down)\|MI:0007(anti tag coimmunoprecipitation)\|MI:0018(two hybrid)\|MI:0004(affinity chromatography technology) |
| PRPF19 | PRPF6 | 0.73 | MI:0401(biochemical) |
| PRPF4 | PRPF6 | 0.98 | Affinity Capture-MS\|MI:0007(anti tag coimmunoprecipitation)\|MI:0004(affinity chromatography technology)\|MI:0401(biochemical) |
| PRPF4B | PRPF6 | 0.72 | MI:0676(tandem affinity purification)\|MI:0004(affinity chromatography technology) |
| PRPF6 | ARAF | 0.75 | MI:0492(in vitro)\|MI:0018(two hybrid) |
| PRPF6 | AR | 0.88 | MI:0493(in vivo)\|Affinity Capture-Western\|Reconstituted Complex\|MI:0004(affinity chromatography technology)\|MI:0096(pull down) |
| PRPF6 | PRPF6 | 0.81 | MI:0493(in vivo)\|MI:0018(two hybrid)\|MI:0096(pull down) |
| PRPF6 | ESR1 | 0.75 | MI:0493(in vivo)\|MI:0004(affinity chromatography technology) |
| PRPF6 | PRPF31 | 0.99 | MI:0018(two hybrid)\|MI:0007(anti tag coimmunoprecipitation)\|MI:0004(affinity chromatography technology)\|MI:0096(pull down)\|MI:0401(biochemical) |
| PRPF6 | IK | 0.76 | MI:0018(two hybrid)\|MI:0007(anti tag coimmunoprecipitation)\|MI:0004(affinity chromatography technology) |
| PRPF6 | SNRNP200 | 0.99 | MI:0004(affinity chromatography technology)\|MI:0018(two hybrid)\|MI:0676(tandem affinity purification)\|MI:0096(pull down)\|MI:0401(biochemical) |
| PRPF6 | EFTUD2 | 0.89 | MI:0018(two hybrid)\|MI:0004(affinity chromatography technology)\|MI:0676(tandem affinity purification)\|MI:0096(pull down)\|MI:0401(biochemical) |
| PRPF6 | PRPF3 | 0.88 | MI:0004(affinity chromatography technology)\|MI:0096(pull down)\|MI:0007(anti tag coimmunoprecipitation)\|MI:0401(biochemical) |
| PRPF6 | PRPF8 | 0.99 | MI:0004(affinity chromatography technology)\|MI:0018(two hybrid)\|MI:0676(tandem affinity purification)\|MI:0096(pull down)\|bioid\|MI:0401(biochemical) |
| PRPF6 | SF3B3 | 0.7 | MI:0401(biochemical) |
| PRPF6 | RPS24 | 0.82 | bioid\|MI:0401(biochemical) |
| PRPF6 | SNRPA1 | 0.7 | MI:0401(biochemical) |
| PRPF6 | SNRPD1 | 0.82 | MI:0401(biochemical) |
| PRPF6 | SNRPD3 | 0.88 | MI:0004(affinity chromatography technology)\|MI:0401(biochemical) |
| PRPF6 | SNRPD2 | 0.79 | MI:0401(biochemical) |
| PRPF6 | SF3A3 | 0.7 | MI:0401(biochemical) |
| PRPF6 | SMU1 | 0.72 | MI:0004(affinity chromatography technology)\|MI:0676(tandem affinity purification) |
| PRPF6 | RIOK1 | 0.72 | MI:0004(affinity chromatography technology)\|MI:0676(tandem affinity purification) |
| PTAFR | PTK2 | 0.72 | MI:0004(affinity chromatography technology)\|MI:0006(anti bait coimmunoprecipitation) |
| PTEN | PTK2 | 0.87 | Affinity Capture-Western\|MI:0004(affinity chromatography technology)\|MI:0096(pull down)\|MI:0415(enzymatic study)\|MI:0428(imaging technique) |
| PTK2 | FGR | 0.73 | MI:0493(in vivo)\|Affinity Capture-Western\|MI:0004(affinity chromatography technology) |
| PTK2 | LYN | 0.89 | MI:0493(in vivo)\|Affinity Capture-Western\|MI:0019(coimmunoprecipitation)\|MI:0004(affinity chromatography technology)\|MI:0018(two hybrid) |
| PTK2 | PIK3R1 | 0.88 | MI:0493(in vivo)\|Affinity Capture-Western\|MI:0047(far western blotting)\|MI:0004(affinity chromatography technology) |
| PTK2 | PLCG1 | 0.92 | MI:0492(in vitro)\|MI:0493(in vivo)\|Affinity Capture-Western\|MI:0004(affinity chromatography technology)\|MI:0428(imaging technique) |
| PTK2 | ITGB3 | 0.9 | MI:0492(in vitro)\|MI:0493(in vivo)\|Reconstituted Complex\|Affinity Capture-Western\|MI:0004(affinity chromatography technology)\|MI:0096(pull down) |
| PTK2 | PTPN11 | 0.88 | MI:0492(in vitro)\|MI:0493(in vivo)\|Affinity Capture-Western\|MI:0004(affinity chromatography technology)\|MI:0006(anti bait coimmunoprecipitation) |
| PTK2 | EPHA2 | 0.77 | MI:0493(in vivo)\|Affinity Capture-Western\|MI:0004(affinity chromatography technology)\|MI:0416(fluorescence microscopy)\|MI:0006(anti bait coimmunoprecipitation) |
| PTK2 | TLN1 | 0.89 | MI:0492(in vitro)\|MI:0493(in vivo)\|Affinity Capture-Western\|Reconstituted Complex\|MI:0004(affinity chromatography technology)\|MI:0096(pull down) |
| PTK2 | GRB2 | 0.97 | MI:0492(in vitro)\|MI:0493(in vivo)\|Affinity Capture-Western\|Reconstituted Complex\|MI:0004(affinity chromatography technology)\|MI:0018(two hybrid)\|MI:0096(pull down)\|MI:0006(anti bait coimmunoprecipitation) |
| PTK2 | ITGAV | 0.78 | MI:0492(in vitro)\|MI:0004(affinity chromatography technology)\|MI:0428(imaging technique) |
| PTK2 | BMX | 0.88 | MI:0492(in vitro)\|MI:0493(in vivo)\|Affinity Capture-Western\|Reconstituted Complex\|MI:0004(affinity chromatography technology)\|MI:0096(pull down) |
| PTK2 | SYK | 0.84 | MI:0493(in vivo)\|Affinity Capture-Western\|MI:0004(affinity chromatography technology) |
| PTK2 | STAT1 | 0.9 | MI:0492(in vitro)\|MI:0493(in vivo)\|Affinity Capture-Western\|Reconstituted Complex\|MI:0004(affinity chromatography technology)\|MI:0096(pull down)\|MI:0428(imaging technique)\|MI:0006(anti bait coimmunoprecipitation) |
| PTK2 | SHC1 | 0.92 | MI:0492(in vitro)\|MI:0493(in vivo)\|Affinity Capture-Western\|MI:0004(affinity chromatography technology) |
| PTK2 | PTK2 | 0.96 | MI:0492(in vitro)\|MI:0493(in vivo)\|Co-crystal Structure\|MI:0114(x-ray crystallography)\|MI:0096(pull down)\|MI:0415(enzymatic study) |
| PTK2 | PXN | 0.97 | MI:0492(in vitro)\|MI:0493(in vivo)\|Affinity Capture-Western\|Reconstituted Complex\|MI:0096(pull down)\|MI:0007(anti tag coimmunoprecipitation)\|MI:0004(affinity chromatography technology)\|MI:0663(confocal microscopy)\|MI:0006(anti bait coimmunoprecipitation)\|MI:0047(far western blotting)\|MI:0416(fluorescence microscopy)\|bioid |
| PTK2 | BCAR1 | 0.97 | MI:0492(in vitro)\|MI:0493(in vivo)\|MI:0018(two hybrid)\|Affinity Capture-Western\|Reconstituted Complex\|MI:0049(filter binding)\|MI:0004(affinity chromatography technology)\|MI:0019(coimmunoprecipitation)\|MI:0096(pull down) |
| PTK2 | EZR | 0.86 | MI:0492(in vitro)\|MI:0493(in vivo)\|Reconstituted Complex\|MI:0096(pull down) |
| PTK2 | TP53 | 0.9 | MI:0492(in vitro)\|MI:0493(in vivo)\|Affinity Capture-Western\|Reconstituted Complex\|MI:0006(anti bait coimmunoprecipitation)\|MI:0663(confocal microscopy)\|MI:0096(pull down)\|MI:0004(affinity chromatography technology)\|MI:0428(imaging technique)\|MI:0401(biochemical) |
| PTK2 | CSK | 0.83 | MI:0492(in vitro)\|MI:0493(in vivo)\|Affinity Capture-Western\|MI:0004(affinity chromatography technology) |
| PTK2 | DCC | 0.96 | MI:0492(in vitro)\|MI:0493(in vivo)\|Reconstituted Complex\|Affinity Capture-Western\|MI:0018(two hybrid)\|MI:0004(affinity chromatography technology)\|MI:0006(anti bait coimmunoprecipitation)\|MI:0096(pull down) |
| PTK2 | NEO1 | 0.83 | MI:0492(in vitro)\|Affinity Capture-Western\|MI:0018(two hybrid)\|MI:0004(affinity chromatography technology) |
| PTK2 | SELE | 0.75 | MI:0492(in vitro)\|Affinity Capture-Western\|MI:0004(affinity chromatography technology)\|MI:0096(pull down) |
| PTK2 | EGFR | 0.9 | MI:0492(in vitro)\|MI:0493(in vivo)\|Affinity Capture-Western\|MI:0006(anti bait coimmunoprecipitation)\|MI:0004(affinity chromatography technology)\|MI:0018(two hybrid)\|MI:0112(ubiquitin reconstruction)\|MI:0090(protein complementation assay) |
| PTK2 | ITGB1 | 0.78 | MI:0492(in vitro)\|MI:0004(affinity chromatography technology)\|MI:0428(imaging technique) |
| PTK2 | FYN | 0.97 | MI:0492(in vitro)\|Affinity Capture-Western\|Co-crystal Structure\|MI:0004(affinity chromatography technology)\|MI:0114(x-ray crystallography)\|MI:0096(pull down)\|MI:0399(two hybrid fragment pooling approach) |
| PTK2 | IRS1 | 0.76 | MI:0018(two hybrid)\|Affinity Capture-Western\|MI:0004(affinity chromatography technology) |
| PTK2 | JAK2 | 0.83 | MI:0493(in vivo)\|Affinity Capture-Western\|MI:0004(affinity chromatography technology) |
| PTK2 | ERBB2 | 0.85 | MI:0493(in vivo)\|MI:0019(coimmunoprecipitation)\|MI:0428(imaging technique)\|MI:0004(affinity chromatography technology) |
| PTK2 | DOCK1 | 0.72 | Affinity Capture-Western\|MI:0004(affinity chromatography technology) |
| PTK2 | PCSK1 | 0.72 | Affinity Capture-Western\|MI:0004(affinity chromatography technology) |
| PTK2 | TSC1 | 0.72 | Affinity Capture-Western\|MI:0004(affinity chromatography technology) |
| PTK2 | ITGA4 | 0.72 | Affinity Capture-Western\|MI:0004(affinity chromatography technology) |
| PTK2 | AMPH | 0.72 | Affinity Capture-Western\|MI:0004(affinity chromatography technology) |
| PTK2 | YES1 | 0.88 | Affinity Capture-Western\|MI:0004(affinity chromatography technology)\|MI:0018(two hybrid)\|MI:0428(imaging technique) |
| PTK2 | STAT3 | 0.83 | Affinity Capture-Western\|MI:0004(affinity chromatography technology)\|MI:0428(imaging technique) |
| PTK2 | MDM2 | 0.75 | Reconstituted Complex\|MI:0096(pull down) |
| PTK2 | SOCS3 | 0.88 | MI:0004(affinity chromatography technology)\|MI:0018(two hybrid)\|MI:0096(pull down)\|MI:0047(far western blotting) |
| PTK2 | PTPN23 | 0.79 | MI:0434(phosphatase assay)\|MI:0051(fluorescence technology)\|MI:0424(protein kinase assay)\|MI:0096(pull down)\|MI:0006(anti bait coimmunoprecipitation) |
| PTK2 | PIK3R3 | 0.87 | MI:0006(anti bait coimmunoprecipitation)\|MI:0004(affinity chromatography technology)\|MI:0018(two hybrid) |
| PTK2 | PIK3CA | 0.82 | MI:0006(anti bait coimmunoprecipitation)\|MI:0004(affinity chromatography technology) |
| PTK2 | SRPK1 | 0.83 | MI:0424(protein kinase assay)\|MI:0415(enzymatic study)\|MI:0096(pull down) |
| PTK2 | APC | 0.75 | MI:0428(imaging technique)\|bioid |
| PTK2 | DEF6 | 0.73 | MI:0018(two hybrid) |
| PTK2 | TRIM72 | 0.73 | MI:0004(affinity chromatography technology) |
| PTK2 | SH2D2A | 0.72 | MI:0004(affinity chromatography technology)\|MI:0018(two hybrid) |
| PTK2 | EPB41L5 | 0.78 | MI:0096(pull down)\|bioid |
| PTK2 | SWAP70 | 0.82 | MI:0004(affinity chromatography technology)\|bioid |
| PTK6 | MET | 0.75 | MI:0053(fluorescence polarization spectroscopy)\|MI:0096(pull down) |
| PTPN1 | PTK2 | 0.75 | MI:0434(phosphatase assay) |
| PTPN11 | MET | 0.88 | MI:0493(in vivo)\|MI:0006(anti bait coimmunoprecipitation)\|MI:0053(fluorescence polarization spectroscopy)\|MI:0096(pull down) |
| PTPN12 | PTK2 | 0.78 | MI:0492(in vitro)\|MI:0493(in vivo)\|bioid |
| PTPN12 | PRKDC | 0.72 | MI:0007(anti tag coimmunoprecipitation)\|MI:0004(affinity chromatography technology) |
| PTPN23 | HGS | 0.73 | MI:0004(affinity chromatography technology) |
| PTPN6 | PTK2 | 0.72 | MI:0004(affinity chromatography technology)\|MI:0018(two hybrid) |
| PTPRB | MET | 0.7 | MI:0492(in vitro)\|MI:0434(phosphatase assay) |
| PTPRJ | MET | 0.89 | MI:0492(in vitro)\|MI:0493(in vivo)\|MI:0434(phosphatase assay)\|MI:0004(affinity chromatography technology)\|MI:0019(coimmunoprecipitation)\|MI:0096(pull down) |
| PTPRO | MET | 0.86 | MI:0434(phosphatase assay)\|MI:0004(affinity chromatography technology)\|MI:0096(pull down) |
| PTRH2 | PTK2 | 0.72 | MI:0006(anti bait coimmunoprecipitation)\|MI:0004(affinity chromatography technology) |
| RAB4B | PRPF6 | 0.72 | MI:0004(affinity chromatography technology)\|MI:0007(anti tag coimmunoprecipitation) |
| RAB5A | PRKDC | 0.72 | MI:0007(anti tag coimmunoprecipitation)\|MI:0004(affinity chromatography technology) |
| RAB5A | PTK2 | 0.72 | MI:0007(anti tag coimmunoprecipitation)\|MI:0004(affinity chromatography technology) |
| RAC1 | KALRN | 0.87 | Reconstituted Complex\|MI:0096(pull down)\|MI:0004(affinity chromatography technology) |
| RAD21 | PRKDC | 0.82 | MI:0004(affinity chromatography technology)\|MI:0401(biochemical) |
| RAF1 | MET | 0.75 | MI:0055(fluorescent resonance energy transfer) |
| RANBP10 | MET | 0.79 | MI:0492(in vitro)\|MI:0493(in vivo)\|Affinity Capture-Western\|Reconstituted Complex\|MI:0004(affinity chromatography technology)\|MI:0096(pull down) |
| RANBP9 | MET | 0.79 | MI:0492(in vitro)\|MI:0493(in vivo)\|Reconstituted Complex\|Affinity Capture-Western\|MI:0018(two hybrid)\|MI:0019(coimmunoprecipitation)\|MI:0096(pull down)\|MI:0004(affinity chromatography technology) |
| RAPGEF2 | MAGI2 | 0.74 | MI:0492(in vitro)\|MI:0493(in vivo)\|MI:0007(anti tag coimmunoprecipitation)\|MI:0049(filter binding) |
| RASSF1 | MET | 0.75 | MI:0055(fluorescent resonance energy transfer) |
| RB1CC1 | PTK2 | 0.79 | MI:0492(in vitro)\|MI:0493(in vivo)\|MI:0018(two hybrid)\|Reconstituted Complex\|Affinity Capture-Western\|MI:0004(affinity chromatography technology)\|MI:0096(pull down) |
| RBM42 | PRPF6 | 0.72 | MI:0004(affinity chromatography technology)\|MI:0007(anti tag coimmunoprecipitation) |
| RECQL5 | PRKDC | 0.72 | Affinity Capture-MS\|MI:0004(affinity chromatography technology) |
| RELA | PRKDC | 0.86 | MI:0676(tandem affinity purification)\|MI:0007(anti tag coimmunoprecipitation)\|MI:0004(affinity chromatography technology) |
| RELB | PRKDC | 0.72 | MI:0676(tandem affinity purification)\|MI:0007(anti tag coimmunoprecipitation) |
| RFC2 | PRKDC | 0.72 | MI:0004(affinity chromatography technology)\|MI:0401(biochemical) |
| RHBDL1 | MAGI2 | 0.82 | MI:0004(affinity chromatography technology)\|MI:0007(anti tag coimmunoprecipitation) |
| RHOA | PRKDC | 0.72 | MI:0007(anti tag coimmunoprecipitation)\|MI:0004(affinity chromatography technology) |
| RIPK3 | PRKDC | 0.72 | MI:0676(tandem affinity purification)\|MI:0007(anti tag coimmunoprecipitation) |
| RIPK4 | PRKCH | 0.72 | MI:0004(affinity chromatography technology)\|MI:0007(anti tag coimmunoprecipitation) |
| RIPK4 | PRKDC | 0.72 | MI:0004(affinity chromatography technology)\|MI:0007(anti tag coimmunoprecipitation) |
| RNF144A | PRKDC | 0.81 | MI:0415(enzymatic study)\|MI:0004(affinity chromatography technology)\|MI:0096(pull down) |
| RNF144B | PRKDC | 0.7 | MI:0415(enzymatic study)\|MI:0004(affinity chromatography technology)\|MI:0096(pull down) |
| RNF4 | PRKDC | 0.73 | MI:0004(affinity chromatography technology) |
| RNPS1 | PRPF6 | 0.88 | Affinity Capture-MS\|MI:0006(anti bait coimmunoprecipitation)\|MI:0004(affinity chromatography technology)\|MI:0007(anti tag coimmunoprecipitation) |
| RNU11 | PRPF6 | 0.72 | Affinity Capture-MS\|MI:0004(affinity chromatography technology) |
| RNU12-2P | PRPF6 | 0.72 | Affinity Capture-MS\|MI:0004(affinity chromatography technology) |
| RPS14 | CERK | 0.72 | MI:0030(cross-linking study)\|bioid |
| RSBN1L | CLK2 | 0.82 | MI:0004(affinity chromatography technology)\|MI:0007(anti tag coimmunoprecipitation) |
| RTCB | PRKDC | 0.72 | MI:0004(affinity chromatography technology)\|MI:0401(biochemical) |
| RTRAF | PRKDC | 0.72 | MI:0004(affinity chromatography technology)\|MI:0401(biochemical) |
| RUVBL1 | PRKDC | 0.78 | MI:0096(pull down)\|MI:0004(affinity chromatography technology) |
| SART1 | PRPF6 | 0.89 | MI:0004(affinity chromatography technology)\|MI:0018(two hybrid)\|MI:0096(pull down)\|MI:0006(anti bait coimmunoprecipitation)\|MI:0401(biochemical) |
| SCGB1D1 | MET | 0.82 | MI:0004(affinity chromatography technology)\|MI:0007(anti tag coimmunoprecipitation) |
| SDHA | MAGI2 | 0.72 | MI:0030(cross-linking study)\|bioid |
| SERPINB2 | PTK2 | 0.82 | MI:0004(affinity chromatography technology)\|MI:0007(anti tag coimmunoprecipitation) |
| SF3A3 | HGS | 0.72 | MI:0004(affinity chromatography technology)\|MI:0401(biochemical) |
| SH2B2 | MET | 0.75 | MI:0053(fluorescence polarization spectroscopy)\|MI:0096(pull down) |
| SH2B3 | MET | 0.75 | MI:0053(fluorescence polarization spectroscopy)\|MI:0096(pull down) |
| SH2D1A | MET | 0.75 | MI:0053(fluorescence polarization spectroscopy)\|MI:0096(pull down) |
| SH2D1A | TNK2 | 0.73 | MI:0018(two hybrid) |
| SH2D1B | MET | 0.75 | MI:0053(fluorescence polarization spectroscopy)\|MI:0096(pull down) |
| SH2D2A | MET | 0.75 | MI:0053(fluorescence polarization spectroscopy)\|MI:0096(pull down) |
| SH3KBP1 | MET | 0.72 | Affinity Capture-Western\|MI:0004(affinity chromatography technology) |
| SHB | MET | 0.75 | MI:0053(fluorescence polarization spectroscopy)\|MI:0096(pull down) |
| SHC1 | MET | 0.89 | MI:0493(in vivo)\|Affinity Capture-Western\|MI:0004(affinity chromatography technology)\|MI:0053(fluorescence polarization spectroscopy)\|MI:0096(pull down) |
| SHC1 | PRKDC | 0.7 | MI:0004(affinity chromatography technology) |
| SHC2 | MET | 0.75 | MI:0053(fluorescence polarization spectroscopy)\|MI:0096(pull down) |
| SHC4 | MET | 0.75 | MI:0053(fluorescence polarization spectroscopy)\|MI:0096(pull down) |
| SIK1 | SIK3 | 0.82 | MI:0004(affinity chromatography technology)\|MI:0007(anti tag coimmunoprecipitation) |
| SIK3 | CRTC2 | 0.71 | MI:0492(in vitro)\|MI:0493(in vivo)\|MI:0424(protein kinase assay) |
| SIK3 | YWHAE | 0.82 | MI:0676(tandem affinity purification)\|MI:0004(affinity chromatography technology) |
| SIK3 | TRIP6 | 0.73 | MI:0018(two hybrid) |
| SIK3 | YWHAH | 0.83 | MI:0004(affinity chromatography technology)\|MI:0007(anti tag coimmunoprecipitation) |
| SIKE1 | STK24 | 0.89 | Affinity Capture-MS\|MI:0007(anti tag coimmunoprecipitation)\|MI:0004(affinity chromatography technology)\|MI:0676(tandem affinity purification)\|MI:0096(pull down) |
| SIRT6 | PRKDC | 0.75 | MI:0004(affinity chromatography technology)\|MI:0428(imaging technique) |
| SLA2 | MET | 0.75 | MI:0053(fluorescence polarization spectroscopy)\|MI:0096(pull down) |
| SLK | HGS | 0.72 | MI:0004(affinity chromatography technology)\|MI:0401(biochemical) |
| SMAD2 | SIK3 | 0.72 | MI:0676(tandem affinity purification)\|MI:0004(affinity chromatography technology) |
| SMAD3 | SIK3 | 0.72 | MI:0676(tandem affinity purification)\|MI:0004(affinity chromatography technology) |
| SMAD4 | HGS | 0.73 | MI:0004(affinity chromatography technology) |
| SMAD5 | HGS | 0.72 | MI:0018(two hybrid)\|MI:0004(affinity chromatography technology) |
| SMARCA2 | PRPF6 | 0.77 | MI:0493(in vivo)\|MI:0006(anti bait coimmunoprecipitation)\|MI:0019(coimmunoprecipitation)\|MI:0004(affinity chromatography technology) |
| SMDT1 | MYLK2 | 0.83 | MI:0004(affinity chromatography technology)\|MI:0007(anti tag coimmunoprecipitation) |
| SNAP25 | HGS | 0.88 | MI:0018(two hybrid)\|MI:0004(affinity chromatography technology) |
| SNCAIP | KALRN | 0.77 | MI:0007(anti tag coimmunoprecipitation)\|MI:0663(confocal microscopy)\|MI:0018(two hybrid)\|MI:0004(affinity chromatography technology) |
| SNRNP40 | PRPF6 | 0.89 | Affinity Capture-MS\|MI:0006(anti bait coimmunoprecipitation)\|MI:0004(affinity chromatography technology)\|MI:0007(anti tag coimmunoprecipitation)\|MI:0096(pull down) |
| SNRNP70 | PRPF6 | 0.82 | MI:0004(affinity chromatography technology)\|MI:0018(two hybrid) |
| SNRPA | PRKDC | 0.72 | MI:0004(affinity chromatography technology)\|MI:0401(biochemical) |
| SNRPA | PRPF6 | 0.82 | MI:0018(two hybrid)\|MI:0401(biochemical) |
| SNRPB | PRPF6 | 0.82 | MI:0006(anti bait coimmunoprecipitation)\|MI:0004(affinity chromatography technology) |
| SNRPE | PRPF6 | 0.91 | MI:0004(affinity chromatography technology)\|MI:0007(anti tag coimmunoprecipitation) |
| SNRPF | PRPF6 | 0.94 | MI:0004(affinity chromatography technology)\|MI:0007(anti tag coimmunoprecipitation)\|MI:0018(two hybrid) |
| SNRPG | PRPF6 | 0.79 | MI:0004(affinity chromatography technology) |
| SNRPN | PRPF6 | 0.82 | MI:0004(affinity chromatography technology)\|MI:0007(anti tag coimmunoprecipitation) |
| SNW1 | PRPF6 | 0.72 | MI:0006(anti bait coimmunoprecipitation)\|MI:0004(affinity chromatography technology) |
| SNW1 | PRKDC | 0.72 | MI:0006(anti bait coimmunoprecipitation)\|MI:0004(affinity chromatography technology) |
| SNX9 | TNK2 | 0.88 | MI:0492(in vitro)\|MI:0493(in vivo)\|MI:0081(peptide array)\|MI:0007(anti tag coimmunoprecipitation)\|MI:0004(affinity chromatography technology) |
| SOCS1 | MET | 0.87 | MI:0053(fluorescence polarization spectroscopy)\|MI:0004(affinity chromatography technology)\|MI:0096(pull down) |
| SOCS1 | PRKDC | 0.72 | MI:0007(anti tag coimmunoprecipitation)\|MI:0004(affinity chromatography technology) |
| SOCS3 | MET | 0.75 | MI:0053(fluorescence polarization spectroscopy)\|MI:0096(pull down) |
| SOCS6 | MET | 0.75 | MI:0053(fluorescence polarization spectroscopy)\|MI:0096(pull down) |
| SP1 | PRKCZ | 0.75 | MI:0415(enzymatic study)\|MI:0004(affinity chromatography technology) |
| SQSTM1 | PRKCZ | 0.97 | MI:0492(in vitro)\|MI:0493(in vivo)\|MI:0018(two hybrid)\|Affinity Capture-Western\|MI:0004(affinity chromatography technology)\|MI:0096(pull down)\|MI:0007(anti tag coimmunoprecipitation)\|MI:0428(imaging technique)\|bioid |
| SQSTM1 | HGS | 0.73 | bioid |
| SRC | PRKCZ | 0.86 | MI:0493(in vivo)\|Affinity Capture-Western\|MI:0004(affinity chromatography technology)\|MI:0096(pull down) |
| SRC | PTK2 | 0.97 | MI:0492(in vitro)\|MI:0493(in vivo)\|Affinity Capture-Western\|Reconstituted Complex\|MI:0018(two hybrid)\|MI:0006(anti bait coimmunoprecipitation)\|MI:0019(coimmunoprecipitation)\|MI:0007(anti tag coimmunoprecipitation)\|MI:0004(affinity chromatography technology)\|MI:0096(pull down)\|MI:0428(imaging technique)\|MI:0415(enzymatic study) |
| SRC | MET | 0.9 | MI:0493(in vivo)\|Affinity Capture-Western\|MI:0004(affinity chromatography technology)\|MI:0006(anti bait coimmunoprecipitation)\|MI:0053(fluorescence polarization spectroscopy)\|MI:0096(pull down) |
| SRC | TNK2 | 0.79 | MI:0096(pull down)\|MI:0004(affinity chromatography technology) |
| SREBF1 | CDK8 | 0.78 | MI:0096(pull down)\|MI:0004(affinity chromatography technology) |
| SRRM2 | PRPF6 | 0.82 | MI:0018(two hybrid)\|MI:0401(biochemical) |
| SRSF3 | CLK2 | 0.82 | MI:0018(two hybrid)\|MI:0004(affinity chromatography technology) |
| STAT5A | PDK2 | 0.82 | MI:0004(affinity chromatography technology)\|MI:0007(anti tag coimmunoprecipitation) |
| STK24 | STRN | 0.9 | Affinity Capture-MS\|Affinity Capture-Western\|MI:0007(anti tag coimmunoprecipitation)\|MI:0004(affinity chromatography technology)\|MI:0006(anti bait coimmunoprecipitation)\|MI:0676(tandem affinity purification)\|MI:0096(pull down)\|bioid |
| STK24 | STRN3 | 0.9 | Affinity Capture-MS\|MI:0007(anti tag coimmunoprecipitation)\|MI:0004(affinity chromatography technology)\|MI:0006(anti bait coimmunoprecipitation)\|MI:0676(tandem affinity purification)\|MI:0096(pull down)\|bioid |
| STK24 | PPP2R1A | 0.9 | Affinity Capture-MS\|MI:0007(anti tag coimmunoprecipitation)\|MI:0004(affinity chromatography technology)\|MI:0006(anti bait coimmunoprecipitation)\|MI:0676(tandem affinity purification)\|MI:0096(pull down) |
| STK24 | STRN4 | 0.9 | Affinity Capture-MS\|MI:0007(anti tag coimmunoprecipitation)\|MI:0004(affinity chromatography technology)\|MI:0006(anti bait coimmunoprecipitation)\|MI:0676(tandem affinity purification)\|MI:0096(pull down) |
| STK24 | STK25 | 0.9 | Affinity Capture-MS\|MI:0007(anti tag coimmunoprecipitation)\|MI:0004(affinity chromatography technology)\|MI:0006(anti bait coimmunoprecipitation)\|MI:0676(tandem affinity purification)\|MI:0096(pull down) |
| STK24 | STRIP1 | 0.88 | Affinity Capture-MS\|MI:0007(anti tag coimmunoprecipitation)\|MI:0004(affinity chromatography technology)\|MI:0096(pull down) |
| STK24 | PPP2CB | 0.89 | Affinity Capture-MS\|MI:0004(affinity chromatography technology)\|MI:0006(anti bait coimmunoprecipitation)\|MI:0676(tandem affinity purification)\|MI:0096(pull down) |
| STK24 | SLMAP | 0.9 | Affinity Capture-MS\|MI:0007(anti tag coimmunoprecipitation)\|MI:0004(affinity chromatography technology)\|MI:0006(anti bait coimmunoprecipitation)\|MI:0676(tandem affinity purification)\|MI:0096(pull down) |
| STK24 | APOD | 0.76 | Affinity Capture-MS\|MI:0004(affinity chromatography technology)\|MI:0006(anti bait coimmunoprecipitation) |
| STK24 | DDX5 | 0.76 | Affinity Capture-MS\|MI:0004(affinity chromatography technology)\|MI:0006(anti bait coimmunoprecipitation) |
| STK24 | G3BP2 | 0.76 | Affinity Capture-MS\|MI:0004(affinity chromatography technology)\|MI:0006(anti bait coimmunoprecipitation) |
| STK24 | HNRNPH3 | 0.86 | Affinity Capture-MS\|MI:0004(affinity chromatography technology)\|MI:0006(anti bait coimmunoprecipitation) |
| STK24 | PDCD6IP | 0.76 | Affinity Capture-MS\|MI:0004(affinity chromatography technology)\|MI:0006(anti bait coimmunoprecipitation) |
| STK24 | ZBTB24 | 0.72 | Affinity Capture-MS\|MI:0004(affinity chromatography technology) |
| STK24 | JPH3 | 0.76 | Affinity Capture-MS\|MI:0004(affinity chromatography technology)\|MI:0006(anti bait coimmunoprecipitation) |
| STK24 | SYNCRIP | 0.76 | Affinity Capture-MS\|MI:0004(affinity chromatography technology)\|MI:0006(anti bait coimmunoprecipitation) |
| STK24 | HNRNPA3 | 0.76 | Affinity Capture-MS\|MI:0004(affinity chromatography technology)\|MI:0006(anti bait coimmunoprecipitation) |
| STK24 | FABP5 | 0.72 | Affinity Capture-MS\|MI:0004(affinity chromatography technology) |
| STK24 | DSP | 0.76 | Affinity Capture-MS\|MI:0004(affinity chromatography technology)\|MI:0006(anti bait coimmunoprecipitation) |
| STK24 | PPP2CA | 0.89 | Affinity Capture-MS\|MI:0007(anti tag coimmunoprecipitation)\|MI:0004(affinity chromatography technology)\|MI:0676(tandem affinity purification)\|MI:0096(pull down) |
| STK24 | PPP2R1B | 0.89 | Affinity Capture-MS\|MI:0007(anti tag coimmunoprecipitation)\|MI:0004(affinity chromatography technology)\|MI:0676(tandem affinity purification)\|MI:0096(pull down) |
| STK24 | STRIP2 | 0.89 | Affinity Capture-MS\|MI:0007(anti tag coimmunoprecipitation)\|MI:0004(affinity chromatography technology)\|MI:0676(tandem affinity purification)\|MI:0096(pull down) |
| STK24 | CCT2 | 0.76 | Affinity Capture-MS\|MI:0007(anti tag coimmunoprecipitation)\|MI:0004(affinity chromatography technology) |
| STK24 | CCT8 | 0.86 | Affinity Capture-MS\|MI:0007(anti tag coimmunoprecipitation)\|MI:0004(affinity chromatography technology) |
| STK24 | CTTNBP2 | 0.89 | Affinity Capture-MS\|MI:0007(anti tag coimmunoprecipitation)\|MI:0004(affinity chromatography technology)\|MI:0676(tandem affinity purification)\|MI:0096(pull down) |
| STK24 | CCT3 | 0.76 | Affinity Capture-MS\|MI:0007(anti tag coimmunoprecipitation)\|MI:0004(affinity chromatography technology) |
| STK24 | TCP1 | 0.76 | Affinity Capture-MS\|MI:0007(anti tag coimmunoprecipitation)\|MI:0004(affinity chromatography technology) |
| STK24 | CCT5 | 0.76 | Affinity Capture-MS\|MI:0007(anti tag coimmunoprecipitation)\|MI:0004(affinity chromatography technology) |
| STK24 | FGFR1OP2 | 0.89 | Affinity Capture-MS\|MI:0007(anti tag coimmunoprecipitation)\|MI:0004(affinity chromatography technology)\|MI:0676(tandem affinity purification)\|MI:0096(pull down) |
| STK24 | CCT6A | 0.76 | Affinity Capture-MS\|MI:0007(anti tag coimmunoprecipitation)\|MI:0004(affinity chromatography technology) |
| STK24 | CCT7 | 0.76 | Affinity Capture-MS\|MI:0007(anti tag coimmunoprecipitation)\|MI:0004(affinity chromatography technology) |
| STK24 | CCT4 | 0.76 | Affinity Capture-MS\|MI:0007(anti tag coimmunoprecipitation)\|MI:0004(affinity chromatography technology) |
| STK24 | STK26 | 0.88 | MI:0007(anti tag coimmunoprecipitation)\|MI:0676(tandem affinity purification)\|MI:0004(affinity chromatography technology)\|MI:0096(pull down) |
| STK24 | EWSR1 | 0.82 | MI:0006(anti bait coimmunoprecipitation)\|MI:0004(affinity chromatography technology) |
| STK24 | MOB4 | 0.89 | MI:0007(anti tag coimmunoprecipitation)\|MI:0006(anti bait coimmunoprecipitation)\|MI:0004(affinity chromatography technology)\|MI:0676(tandem affinity purification)\|MI:0096(pull down) |
| STK24 | DYNLL1 | 0.84 | MI:0676(tandem affinity purification)\|MI:0004(affinity chromatography technology)\|MI:0096(pull down) |
| STK24 | PROSER2 | 0.87 | MI:0676(tandem affinity purification)\|MI:0004(affinity chromatography technology)\|MI:0007(anti tag coimmunoprecipitation) |
| STK24 | ALDH7A1 | 0.72 | MI:0004(affinity chromatography technology)\|MI:0401(biochemical) |
| STK24 | ASNS | 0.72 | MI:0004(affinity chromatography technology)\|MI:0401(biochemical) |
| STK24 | EIF5 | 0.72 | MI:0004(affinity chromatography technology)\|MI:0401(biochemical) |
| STK24 | TUBB | 0.82 | MI:0004(affinity chromatography technology)\|MI:0401(biochemical) |
| STK24 | ISOC1 | 0.72 | MI:0004(affinity chromatography technology)\|MI:0401(biochemical) |
| STK24 | SCPEP1 | 0.72 | MI:0004(affinity chromatography technology)\|MI:0401(biochemical) |
| STK24 | PSMA5 | 0.78 | MI:0004(affinity chromatography technology)\|MI:0096(pull down) |
| STK24 | PSMC4 | 0.7 | MI:0004(affinity chromatography technology) |
| STK24 | PSMD7 | 0.78 | MI:0004(affinity chromatography technology)\|MI:0096(pull down) |
| STK24 | PSMD13 | 0.78 | MI:0004(affinity chromatography technology)\|MI:0096(pull down) |
| STK24 | PSMD11 | 0.7 | MI:0004(affinity chromatography technology) |
| STK24 | PPP1CA | 0.72 | MI:0004(affinity chromatography technology)\|MI:0401(biochemical) |
| STK24 | MAT2B | 0.72 | MI:0004(affinity chromatography technology)\|MI:0401(biochemical) |
| STK24 | MSN | 0.72 | MI:0004(affinity chromatography technology)\|MI:0401(biochemical) |
| STK24 | XPO1 | 0.72 | MI:0004(affinity chromatography technology)\|MI:0401(biochemical) |
| STK24 | STK24 | 0.75 | MI:0004(affinity chromatography technology)\|MI:0415(enzymatic study) |
| STK24 | PDIA4 | 0.72 | MI:0004(affinity chromatography technology)\|MI:0401(biochemical) |
| STK24 | PSMD6 | 0.78 | MI:0004(affinity chromatography technology)\|MI:0096(pull down) |
| STK24 | CAB39 | 0.7 | MI:0018(two hybrid) |
| STK24 | MAP4K4 | 0.78 | MI:0096(pull down)\|MI:0004(affinity chromatography technology) |
| STK35 | PDLIM1 | 0.78 | MI:0492(in vitro)\|MI:0018(two hybrid)\|Reconstituted Complex\|MI:0096(pull down) |
| STK35 | HSP90AB4P | 0.82 | MI:0004(affinity chromatography technology)\|MI:0007(anti tag coimmunoprecipitation) |
| STK35 | FKBP5 | 0.82 | MI:0004(affinity chromatography technology)\|MI:0007(anti tag coimmunoprecipitation) |
| STK35 | HSP90AA5P | 0.82 | MI:0004(affinity chromatography technology)\|MI:0007(anti tag coimmunoprecipitation) |
| STK35 | HSP90AA1 | 0.85 | MI:0004(affinity chromatography technology)\|MI:0007(anti tag coimmunoprecipitation)\|MI:0096(pull down) |
| STK35 | HSP90AA4P | 0.82 | MI:0004(affinity chromatography technology)\|MI:0007(anti tag coimmunoprecipitation) |
| STK35 | HSP90AB1 | 0.85 | MI:0004(affinity chromatography technology)\|MI:0096(pull down)\|MI:0007(anti tag coimmunoprecipitation) |
| STK35 | HSP90AB3P | 0.82 | MI:0004(affinity chromatography technology)\|MI:0007(anti tag coimmunoprecipitation) |
| STK35 | C1orf174 | 0.85 | MI:0004(affinity chromatography technology)\|MI:0096(pull down)\|MI:0007(anti tag coimmunoprecipitation) |
| STK35 | UBP1 | 0.82 | MI:0004(affinity chromatography technology)\|MI:0007(anti tag coimmunoprecipitation) |
| STK35 | TFCP2 | 0.82 | MI:0004(affinity chromatography technology)\|MI:0007(anti tag coimmunoprecipitation) |
| STK35 | CDC37 | 0.85 | MI:0004(affinity chromatography technology)\|MI:0096(pull down)\|MI:0007(anti tag coimmunoprecipitation) |
| STK35 | CTDSPL2 | 0.85 | MI:0004(affinity chromatography technology)\|MI:0096(pull down)\|MI:0007(anti tag coimmunoprecipitation) |
| STK35 | PRR14L | 0.82 | MI:0004(affinity chromatography technology)\|MI:0007(anti tag coimmunoprecipitation) |
| STK35 | CDC37L1 | 0.82 | MI:0004(affinity chromatography technology)\|MI:0007(anti tag coimmunoprecipitation) |
| STUB1 | PRKCZ | 0.71 | MI:0415(enzymatic study)\|MI:0004(affinity chromatography technology)\|MI:0096(pull down)\|MI:0428(imaging technique) |
| SUMO2 | PRKDC | 0.79 | MI:0004(affinity chromatography technology)\|MI:0096(pull down) |
| SUMO2 | PRPF6 | 0.79 | MI:0004(affinity chromatography technology)\|MI:0096(pull down) |
| SYCE3 | MAP3K15 | 0.73 | MI:0004(affinity chromatography technology) |
| SYT12 | PDK2 | 0.82 | MI:0004(affinity chromatography technology)\|MI:0007(anti tag coimmunoprecipitation) |
| TADA2A | HGS | 0.74 | MI:0018(two hybrid) |
| TADA2A | CDK8 | 0.88 | MI:0004(affinity chromatography technology)\|bioid |
| TBCB | STK24 | 0.72 | MI:0004(affinity chromatography technology)\|MI:0401(biochemical) |
| TCEA1 | CDK8 | 0.94 | Reconstituted Complex\|MI:0096(pull down)\|MI:0401(biochemical) |
| TEC | MET | 0.75 | MI:0053(fluorescence polarization spectroscopy)\|MI:0096(pull down) |
| TELO2 | PRKDC | 0.87 | MI:0004(affinity chromatography technology)\|MI:0006(anti bait coimmunoprecipitation)\|MI:0676(tandem affinity purification) |
| TEX101 | HGS | 0.72 | MI:0006(anti bait coimmunoprecipitation)\|MI:0004(affinity chromatography technology) |
| TGFA | MAGI2 | 0.75 | MI:0492(in vitro)\|MI:0018(two hybrid)\|MI:0059(gst pull down)\|MI:0096(pull down) |
| TGFB1I1 | PTK2 | 0.97 | MI:0492(in vitro)\|MI:0493(in vivo)\|Reconstituted Complex\|Affinity Capture-Western\|MI:0006(anti bait coimmunoprecipitation)\|MI:0004(affinity chromatography technology)\|MI:0018(two hybrid)\|MI:0096(pull down) |
| THRA | CDK8 | 0.72 | Affinity Capture-Western\|MI:0004(affinity chromatography technology) |
| TIAM2 | PRKDC | 0.72 | MI:0030(cross-linking study)\|bioid |
| TINF2 | PRKDC | 0.72 | MI:0004(affinity chromatography technology)\|MI:0007(anti tag coimmunoprecipitation) |
| TMEM260 | MYLK2 | 0.83 | MI:0004(affinity chromatography technology)\|MI:0007(anti tag coimmunoprecipitation) |
| TMPRSS2 | MET | 0.72 | MI:0004(affinity chromatography technology)\|bioid |
| TMPRSS4 | MET | 0.72 | MI:0004(affinity chromatography technology)\|bioid |
| TNFRSF1A | PRKDC | 0.72 | MI:0676(tandem affinity purification)\|MI:0007(anti tag coimmunoprecipitation) |
| TNFRSF1B | PRKDC | 0.72 | MI:0676(tandem affinity purification)\|MI:0007(anti tag coimmunoprecipitation) |
| TNFRSF1B | MYLK2 | 0.72 | MI:0676(tandem affinity purification)\|MI:0007(anti tag coimmunoprecipitation) |
| TNFRSF9 | CERK | 0.83 | MI:0004(affinity chromatography technology)\|MI:0007(anti tag coimmunoprecipitation) |
| TNFSF13B | MAP3K6 | 0.83 | MI:0004(affinity chromatography technology)\|MI:0007(anti tag coimmunoprecipitation) |
| TNFSF13B | MAP3K15 | 0.83 | MI:0004(affinity chromatography technology)\|MI:0007(anti tag coimmunoprecipitation) |
| TNK2 | GRB2 | 0.9 | MI:0493(in vivo)\|Affinity Capture-Western\|MI:0007(anti tag coimmunoprecipitation)\|MI:0081(peptide array)\|MI:0004(affinity chromatography technology)\|MI:0018(two hybrid)\|MI:0096(pull down) |
| TNK2 | MCF2 | 0.74 | MI:0492(in vitro)\|MI:0493(in vivo)\|Affinity Capture-Western\|MI:0004(affinity chromatography technology) |
| TNK2 | NCK1 | 0.89 | MI:0493(in vivo)\|MI:0081(peptide array)\|MI:0096(pull down)\|MI:0004(affinity chromatography technology)\|MI:0007(anti tag coimmunoprecipitation) |
| TNK2 | CDC42 | 0.9 | MI:0492(in vitro)\|MI:0493(in vivo)\|Affinity Capture-Western\|MI:0077(nuclear magnetic resonance)\|MI:0004(affinity chromatography technology)\|MI:0018(two hybrid)\|MI:0096(pull down)\|MI:0114(x-ray crystallography)\|MI:0428(imaging technique)\|MI:0055(fluorescent resonance energy transfer) |
| TNK2 | CLTC | 0.9 | MI:0492(in vitro)\|MI:0493(in vivo)\|Reconstituted Complex\|MI:0007(anti tag coimmunoprecipitation)\|MI:0096(pull down)\|MI:0004(affinity chromatography technology) |
| TNK2 | EGFR | 0.85 | MI:0493(in vivo)\|MI:0006(anti bait coimmunoprecipitation)\|MI:0004(affinity chromatography technology)\|MI:0428(imaging technique) |
| TNK2 | FYN | 0.9 | MI:0492(in vitro)\|MI:0493(in vivo)\|Affinity Capture-Western\|Reconstituted Complex\|MI:0004(affinity chromatography technology)\|MI:0096(pull down) |
| TNK2 | BCAR1 | 0.75 | MI:0096(pull down)\|MI:0006(anti bait coimmunoprecipitation)\|MI:0004(affinity chromatography technology) |
| TNK2 | SIAH1 | 0.82 | MI:0018(two hybrid)\|MI:0004(affinity chromatography technology) |
| TNK2 | NEDD4 | 0.8 | MI:0415(enzymatic study)\|MI:0004(affinity chromatography technology)\|MI:0096(pull down) |
| TNK2 | NEDD4L | 0.8 | MI:0004(affinity chromatography technology)\|MI:0096(pull down)\|MI:0428(imaging technique) |
| TNK2 | HSP90AB1 | 0.83 | MI:0004(affinity chromatography technology)\|MI:0007(anti tag coimmunoprecipitation) |
| TNK2 | HSP90AA1 | 0.83 | MI:0004(affinity chromatography technology)\|MI:0007(anti tag coimmunoprecipitation) |
| TNK2 | TNK2 | 0.89 | MI:0415(enzymatic study)\|MI:0424(protein kinase assay)\|MI:0004(affinity chromatography technology)\|MI:0018(two hybrid) |
| TNK2 | AR | 0.84 | MI:0004(affinity chromatography technology)\|MI:0096(pull down)\|MI:0007(anti tag coimmunoprecipitation) |
| TNK2 | PDGFRB | 0.75 | MI:0004(affinity chromatography technology)\|MI:0415(enzymatic study) |
| TNK2 | AKT1 | 0.78 | MI:0004(affinity chromatography technology)\|MI:0096(pull down) |
| TNK2 | CDC37 | 0.83 | MI:0004(affinity chromatography technology)\|MI:0007(anti tag coimmunoprecipitation) |
| TNK2 | SLC25A5 | 0.82 | MI:0004(affinity chromatography technology)\|MI:0007(anti tag coimmunoprecipitation) |
| TNK2 | HSP90AB3P | 0.83 | MI:0004(affinity chromatography technology)\|MI:0007(anti tag coimmunoprecipitation) |
| TNK2 | ISG15 | 0.82 | MI:0004(affinity chromatography technology)\|MI:0007(anti tag coimmunoprecipitation) |
| TNK2 | CCT2 | 0.72 | MI:0007(anti tag coimmunoprecipitation)\|MI:0004(affinity chromatography technology) |
| TNK2 | HSPD1 | 0.72 | MI:0007(anti tag coimmunoprecipitation)\|MI:0004(affinity chromatography technology) |
| TNK2 | CCT8 | 0.72 | MI:0007(anti tag coimmunoprecipitation)\|MI:0004(affinity chromatography technology) |
| TNK2 | CCT4 | 0.72 | MI:0007(anti tag coimmunoprecipitation)\|MI:0004(affinity chromatography technology) |
| TNK2 | CCT6A | 0.72 | MI:0007(anti tag coimmunoprecipitation)\|MI:0004(affinity chromatography technology) |
| TNK2 | TCP1 | 0.72 | MI:0007(anti tag coimmunoprecipitation)\|MI:0004(affinity chromatography technology) |
| TNK2 | MRPL55 | 0.72 | MI:0007(anti tag coimmunoprecipitation)\|MI:0004(affinity chromatography technology) |
| TNK2 | CCT5 | 0.72 | MI:0007(anti tag coimmunoprecipitation)\|MI:0004(affinity chromatography technology) |
| TNK2 | TUFM | 0.72 | MI:0007(anti tag coimmunoprecipitation)\|MI:0004(affinity chromatography technology) |
| TNK2 | CCT3 | 0.72 | MI:0007(anti tag coimmunoprecipitation)\|MI:0004(affinity chromatography technology) |
| TNS3 | PTK2 | 0.85 | MI:0047(far western blotting)\|MI:0416(fluorescence microscopy)\|MI:0096(pull down)\|MI:0004(affinity chromatography technology) |
| TNS4 | MET | 0.75 | MI:0053(fluorescence polarization spectroscopy)\|MI:0096(pull down) |
| TOP1 | PRKDC | 0.85 | MI:0006(anti bait coimmunoprecipitation)\|MI:0096(pull down)\|MI:0004(affinity chromatography technology)\|MI:0415(enzymatic study) |
| TP53 | MET | 0.75 | MI:0055(fluorescent resonance energy transfer) |
| TPCN2 | CERK | 0.82 | MI:0004(affinity chromatography technology)\|MI:0007(anti tag coimmunoprecipitation) |
| TPTE | PRKDC | 0.75 | MI:0004(affinity chromatography technology)\|MI:0096(pull down)\|bioid |
| TRA2A | CLK2 | 0.89 | MI:0004(affinity chromatography technology)\|MI:0007(anti tag coimmunoprecipitation) |
| TRADD | PRKDC | 0.72 | MI:0676(tandem affinity purification)\|MI:0007(anti tag coimmunoprecipitation) |
| TRAF2 | MYLK2 | 0.72 | MI:0676(tandem affinity purification)\|MI:0007(anti tag coimmunoprecipitation) |
| TRAF3IP3 | STK24 | 0.78 | Affinity Capture-MS\|Affinity Capture-Western\|MI:0007(anti tag coimmunoprecipitation)\|MI:0004(affinity chromatography technology) |
| TRAF6 | PRKCZ | 0.83 | MI:0493(in vivo)\|Affinity Capture-Western\|MI:0004(affinity chromatography technology) |
| TRAK1 | HGS | 0.7 | MI:0004(affinity chromatography technology)\|MI:0096(pull down)\|MI:0428(imaging technique) |
| TRIM41 | PRKCZ | 0.83 | MI:0004(affinity chromatography technology)\|MI:0007(anti tag coimmunoprecipitation) |
| TRIP6 | PTK2 | 0.84 | MI:0493(in vivo)\|Affinity Capture-Western\|MI:0004(affinity chromatography technology) |
| TRRAP | CDK8 | 0.75 | MI:0006(anti bait coimmunoprecipitation)\|MI:0226(ion exchange chromatography)\|MI:0401(biochemical) |
| TSC2 | PTK2 | 0.78 | Affinity Capture-Western\|Reconstituted Complex\|MI:0004(affinity chromatography technology)\|MI:0096(pull down) |
| TSSC4 | PRPF6 | 0.83 | MI:0004(affinity chromatography technology)\|MI:0007(anti tag coimmunoprecipitation) |
| TTC4 | HGS | 0.72 | MI:0004(affinity chromatography technology)\|MI:0401(biochemical) |
| TTI1 | PRKDC | 0.85 | MI:0007(anti tag coimmunoprecipitation)\|MI:0096(pull down)\|MI:0004(affinity chromatography technology) |
| TTI2 | PRKDC | 0.72 | MI:0676(tandem affinity purification)\|MI:0004(affinity chromatography technology) |
| TXK | MET | 0.75 | MI:0053(fluorescence polarization spectroscopy)\|MI:0096(pull down) |
| TXNL4A | PRPF6 | 0.94 | MI:0004(affinity chromatography technology)\|MI:0018(two hybrid)\|MI:0007(anti tag coimmunoprecipitation) |
| TXNL4B | PRPF6 | 0.79 | MI:0492(in vitro)\|MI:0493(in vivo)\|MI:0018(two hybrid)\|Reconstituted Complex\|Affinity Capture-Western\|MI:0004(affinity chromatography technology)\|MI:0096(pull down) |
| U2AF2 | CLK2 | 0.72 | MI:0004(affinity chromatography technology)\|MI:0007(anti tag coimmunoprecipitation) |
| UBL5 | CLK2 | 0.75 | Reconstituted Complex\|MI:0096(pull down) |
| UBQLN2 | HGS | 0.82 | MI:0018(two hybrid)\|MI:0004(affinity chromatography technology) |
| UBR5 | PRKDC | 0.73 | MI:0004(affinity chromatography technology) |
| USF1 | PRKDC | 0.78 | MI:0006(anti bait coimmunoprecipitation)\|MI:0007(anti tag coimmunoprecipitation)\|MI:0004(affinity chromatography technology)\|MI:0424(protein kinase assay) |
| USP39 | PRPF6 | 0.76 | Affinity Capture-MS\|MI:0007(anti tag coimmunoprecipitation)\|MI:0004(affinity chromatography technology) |
| VAPB | SPEG | 0.73 | MI:0004(affinity chromatography technology) |
| VAV3 | MET | 0.75 | MI:0053(fluorescence polarization spectroscopy)\|MI:0096(pull down) |
| VCAM1 | PRKDC | 0.83 | MI:0030(cross-linking study)\|MI:0004(affinity chromatography technology) |
| VCL | STK24 | 0.72 | MI:0004(affinity chromatography technology)\|MI:0401(biochemical) |
| VHL | PRKCZ | 0.85 | MI:0007(anti tag coimmunoprecipitation)\|MI:0022(colocalization by immunostaining)\|MI:0428(imaging technique)\|MI:0004(affinity chromatography technology) |
| VPS16 | PTK2 | 0.72 | MI:0004(affinity chromatography technology)\|MI:0007(anti tag coimmunoprecipitation) |
| VPS37A | HGS | 0.77 | MI:0492(in vitro)\|MI:0493(in vivo)\|MI:0018(two hybrid) |
| VPS37C | HGS | 0.75 | MI:0492(in vitro)\|MI:0018(two hybrid) |
| VWCE | CERK | 0.82 | MI:0004(affinity chromatography technology)\|MI:0007(anti tag coimmunoprecipitation) |
| WASHC1 | HGS | 0.75 | MI:0428(imaging technique)\|MI:0018(two hybrid) |
| WDR76 | PRKDC | 0.82 | MI:0004(affinity chromatography technology)\|MI:0401(biochemical) |
| WDR77 | FAM47E | 0.72 | MI:0007(anti tag coimmunoprecipitation)\|MI:0004(affinity chromatography technology) |
| WRN | PRKDC | 0.88 | MI:0492(in vitro)\|MI:0493(in vivo)\|Protein-peptide\|Affinity Capture-Western\|Biochemical Activity\|MI:0415(enzymatic study)\|MI:0004(affinity chromatography technology) |
| WWC1 | PRKCZ | 0.89 | MI:0415(enzymatic study)\|MI:0018(two hybrid)\|MI:0096(pull down)\|MI:0428(imaging technique)\|MI:0004(affinity chromatography technology)\|MI:0007(anti tag coimmunoprecipitation) |
| WWOX | TNK2 | 0.8 | MI:0493(in vivo)\|MI:0096(pull down)\|MI:0004(affinity chromatography technology) |
| XRCC4 | PRKDC | 0.85 | MI:0492(in vitro)\|MI:0004(affinity chromatography technology)\|MI:0415(enzymatic study)\|MI:0401(biochemical) |
| XRCC5 | PRKDC | 0.9 | MI:0424(protein kinase assay)\|MI:0006(anti bait coimmunoprecipitation)\|MI:0004(affinity chromatography technology)\|MI:0096(pull down)\|MI:0428(imaging technique)\|MI:0415(enzymatic study)\|MI:0114(x-ray crystallography)\|MI:0007(anti tag coimmunoprecipitation)\|MI:0401(biochemical) |
| XRCC5 | MAP3K6 | 0.72 | MI:0004(affinity chromatography technology)\|MI:0007(anti tag coimmunoprecipitation) |
| XRCC6 | PRKDC | 0.9 | MI:0424(protein kinase assay)\|MI:0006(anti bait coimmunoprecipitation)\|MI:0004(affinity chromatography technology)\|MI:0415(enzymatic study)\|MI:0007(anti tag coimmunoprecipitation)\|MI:0401(biochemical) |
| YWHAB | HGS | 0.72 | MI:0007(anti tag coimmunoprecipitation)\|MI:0004(affinity chromatography technology) |
| YWHAB | CLK2 | 0.83 | MI:0007(anti tag coimmunoprecipitation)\|MI:0004(affinity chromatography technology) |
| YWHAB | PFKFB4 | 0.82 | MI:0007(anti tag coimmunoprecipitation)\|MI:0004(affinity chromatography technology) |
| YWHAE | MAP3K15 | 0.72 | MI:0007(anti tag coimmunoprecipitation)\|MI:0004(affinity chromatography technology) |
| YWHAG | CLK2 | 0.89 | MI:0493(in vivo)\|MI:0006(anti bait coimmunoprecipitation)\|MI:0019(coimmunoprecipitation)\|MI:0004(affinity chromatography technology)\|MI:0007(anti tag coimmunoprecipitation) |
| YWHAG | PFKFB4 | 0.82 | MI:0007(anti tag coimmunoprecipitation)\|MI:0004(affinity chromatography technology) |
| YWHAH | MAP3K6 | 0.82 | MI:0007(anti tag coimmunoprecipitation)\|MI:0004(affinity chromatography technology) |
| YWHAH | CLK2 | 0.82 | MI:0007(anti tag coimmunoprecipitation)\|MI:0004(affinity chromatography technology) |
| YWHAH | MAP3K15 | 0.82 | MI:0007(anti tag coimmunoprecipitation)\|MI:0004(affinity chromatography technology) |
| YWHAH | PFKFB4 | 0.82 | MI:0007(anti tag coimmunoprecipitation)\|MI:0004(affinity chromatography technology) |
| YWHAQ | PRKDC | 0.78 | MI:0004(affinity chromatography technology)\|MI:0096(pull down) |
| YWHAQ | MYLK2 | 0.82 | MI:0004(affinity chromatography technology)\|MI:0007(anti tag coimmunoprecipitation) |
| YWHAQ | SPEG | 0.73 | MI:0004(affinity chromatography technology) |
| YWHAQ | CLK2 | 0.83 | MI:0007(anti tag coimmunoprecipitation)\|MI:0004(affinity chromatography technology) |
| YWHAQ | PFKFB4 | 0.82 | MI:0007(anti tag coimmunoprecipitation)\|MI:0004(affinity chromatography technology) |
| YWHAZ | SIK3 | 0.87 | MI:0492(in vitro)\|MI:0493(in vivo)\|MI:0676(tandem affinity purification)\|MI:0096(pull down)\|MI:0004(affinity chromatography technology) |
| YWHAZ | PRKCZ | 0.89 | Reconstituted Complex\|Biochemical Activity\|MI:0415(enzymatic study)\|MI:0096(pull down)\|MI:0004(affinity chromatography technology) |
| YWHAZ | PRKDC | 0.84 | MI:0059(gst pull down)\|MI:0676(tandem affinity purification)\|MI:0096(pull down) |
| YWHAZ | MYLK2 | 0.86 | MI:0676(tandem affinity purification)\|MI:0007(anti tag coimmunoprecipitation)\|MI:0004(affinity chromatography technology) |
| YWHAZ | PFKFB4 | 0.82 | MI:0007(anti tag coimmunoprecipitation)\|MI:0004(affinity chromatography technology) |
| YY1 | PRKDC | 0.71 | MI:0029(cosedimentation through density gradient)\|MI:0096(pull down)\|MI:0401(biochemical) |
| ZC3H18 | CLK2 | 0.82 | MI:0004(affinity chromatography technology)\|MI:0007(anti tag coimmunoprecipitation) |
| ZNHIT2 | PRPF6 | 0.72 | MI:0004(affinity chromatography technology)\|bioid |

**Supplementary Table 4: Top 10 hub genes based on closeness, degree, and betweenness identified within the interacting network of 29 kinases in the HIPPIE- Human Scored Interactions database.**

| Closeness | | | Betweenness | | | Degree | | |
| --- | --- | --- | --- | --- | --- | --- | --- | --- |
| Rank | Name | Score | Rank | Name | Score | Rank | Name | Score |
| 1 | HGS | 471.4333 | 1 | HGS | 348804.6 | 1 | PRKDC | 178 |
| 2 | PRKDC | 467.8333 | 2 | PRKDC | 327053.1 | 2 | HGS | 154 |
| 3 | MET | 438.85 | 3 | PRKCZ | 243982.2 | 3 | PRKCZ | 130 |
| 4 | PRKCZ | 430.6833 | 4 | MET | 209779.1 | 4 | MET | 115 |
| 5 | HSP90AA1 | 429.7167 | 5 | CLK2 | 174258.6 | 5 | PTK2 | 96 |
| 6 | EGFR | 429.0833 | 6 | CDK8 | 151839.1 | 6 | CLK2 | 90 |
| 7 | CLK2 | 418.1167 | 7 | PRPF6 | 149606.2 | 7 | CDK8 | 83 |
| 8 | PRPF6 | 406.05 | 8 | PTK2 | 139314.7 | 8 | PRPF6 | 77 |
| 9 | HSPA8 | 403.5 | 9 | MYLK2 | 138013.1 | 8 | MYLK2 | 77 |
| 10 | MYLK2 | 397.0333 | 10 | STK24 | 123929.2 | 10 | STK24 | 72 |


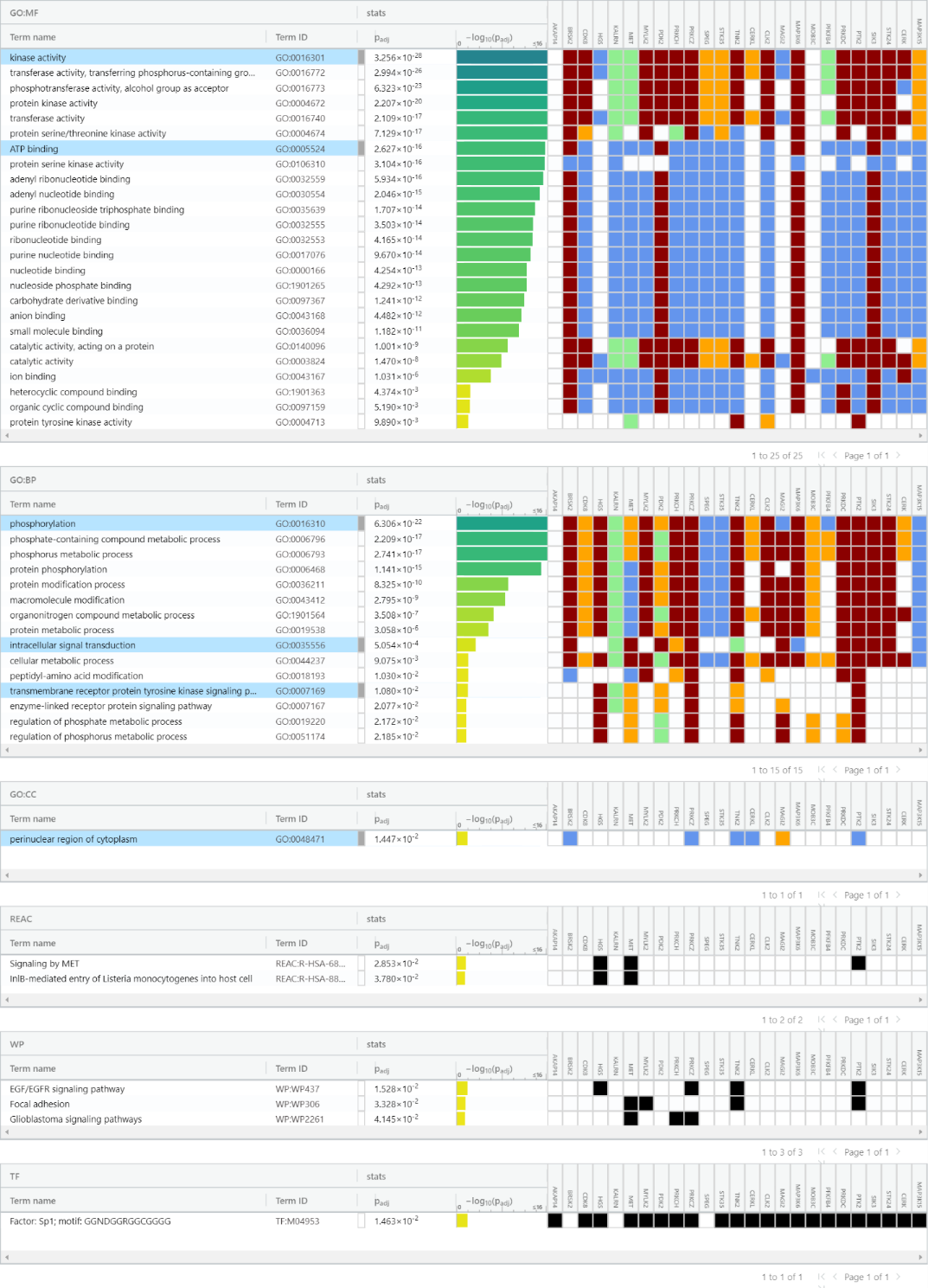


**Supplementary Figure 1:** Gene ontology verification of the hits using g: Profiler


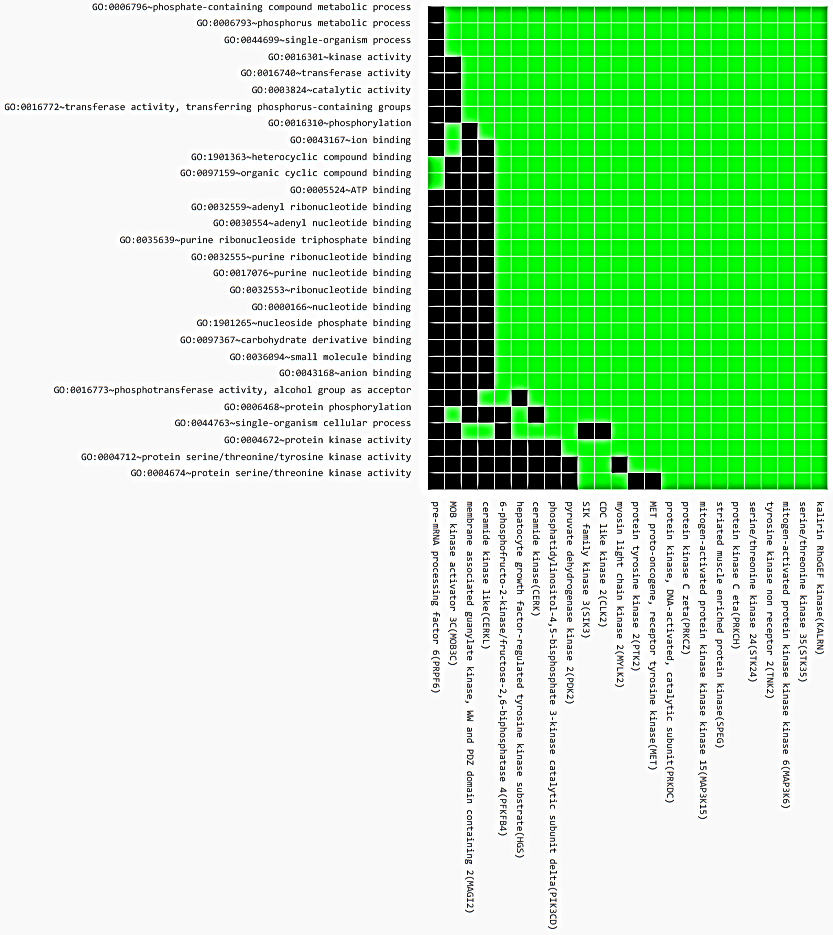


**Supplementary Figure 2:** Gene ontology verification of the hits using DAVID

**
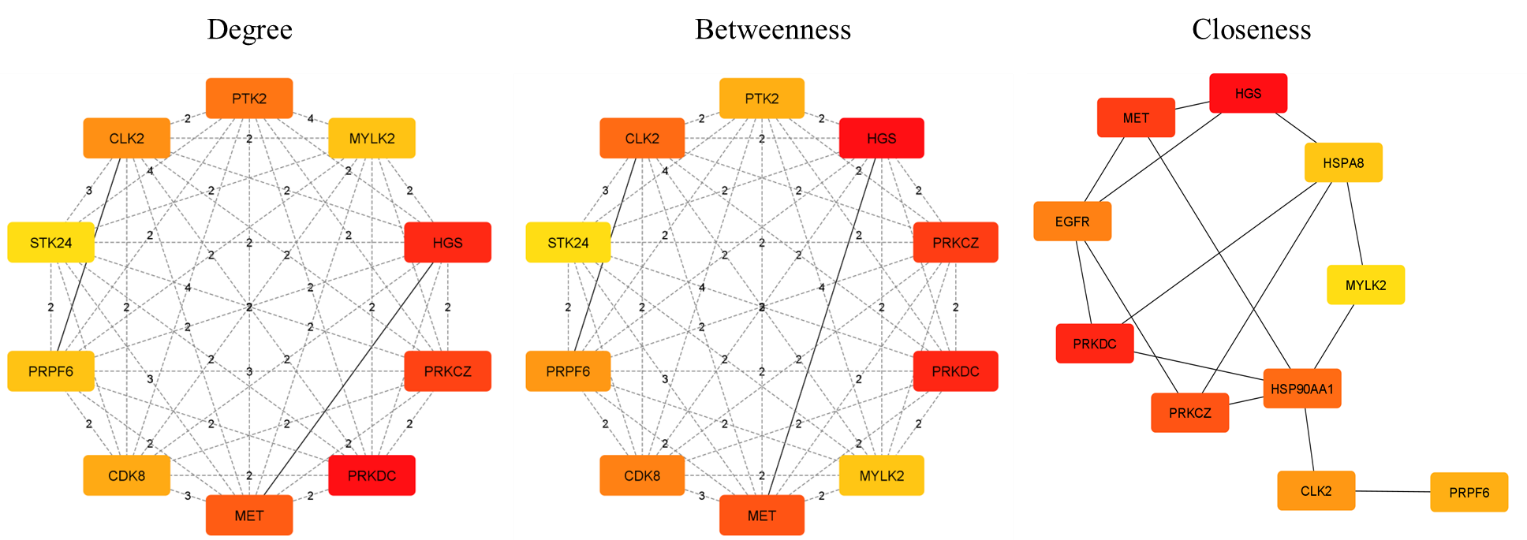
**

**Supplementary Figure 3:** Top 10 hub genes based on closeness, degree, and betweenness identified within the interacting network of 29 kinases in the HIPPIE- Human Scored Interactions database.
